# Melanoma: Genetic Abnormalities, Tumor Progression, Clonal Evolution and Tumor Initiating Cells

**DOI:** 10.3390/medsci5040028

**Published:** 2017-11-20

**Authors:** Ugo Testa, Germana Castelli, Elvira Pelosi

**Affiliations:** Department of Oncology, Istituto Superiore di Sanità, 00161 Rome, Italy; germana.castelli@iss.it (G.C.); elvira.pelosi@iss.it (E.P.)

**Keywords:** melanoma, melanocytes, melanogenesis, cancer stem cells, genomic profiling, membrane cell markers, tumor xenotransplantation assay

## Abstract

Melanoma is an aggressive neoplasia issued from the malignant transformation of melanocytes, the pigment-generating cells of the skin. It is responsible for about 75% of deaths due to skin cancers. Melanoma is a phenotypically and molecularly heterogeneous disease: cutaneous, uveal, acral, and mucosal melanomas have different clinical courses, are associated with different mutational profiles, and possess distinct risk factors. The discovery of the molecular abnormalities underlying melanomas has led to the promising improvement of therapy, and further progress is expected in the near future. The study of melanoma precursor lesions has led to the suggestion that the pathway of tumor evolution implies the progression from benign naevi, to dysplastic naevi, to melanoma in situ and then to invasive and metastatic melanoma. The gene alterations characterizing melanomas tend to accumulate in these precursor lesions in a sequential order. Studies carried out in recent years have, in part, elucidated the great tumorigenic potential of melanoma tumor cells. These findings have led to speculation that the cancer stem cell model cannot be applied to melanoma because, in this malignancy, tumor cells possess an intrinsic plasticity, conferring the capacity to initiate and maintain the neoplastic process to phenotypically different tumor cells.

## 1. Introduction

Sunlight exposure represents the most common environmental risk factor in the development of skin cancer, including melanoma. Skin, eye, and hair color is highly variable in humans, and this great variability is controlled by the amount and the ratio of two different molecular forms of the pigment melanin: the brown-black pigment eumelanin, and the yellow-red pigment pheomelanin. These two types of pigments are produced by melanocytes—cells present at the level of the basal layer of the skin epidermis, hair follicles, and the uvea of the eye. In these cells, the synthesis of eumelanin is stimulated by melanocyte stimulating hormone through a cascade of biochemical events triggered by its interaction with a specific membrane receptor. Reduced signaling at the level of this receptor leads to a decreased synthesis of eumelanin and to an increased synthesis of pheomelanin. Caucasian populations have an increased tendency to develop melanoma when compared to non-Caucasian populations. Within Caucasian populations, individuals with red hair have a particularly high tendency to develop skin melanomas compared to individuals with other hair colors. This finding is not surprising, because red hair is particularly rich in pheomelanin and is virtually deprived of eumelanin. Since eumelanin protects the skin from ultraviolet (UV)-induced DNA damages by absorbing these UV rays, it is easy to understand why red-haired people have a particularly pronounced tendency to accumulate light-induced damage. To investigate the role of pigmentation on melanoma development, Matra and coworkers have recently generated three mouse models: wild-type (black) mice, highly producing eumelanin; albino mice, not producing pigments; red mice, producing a high proportion of pheomelanin. Importantly, even before exposure to UV radiation, 50% of red mice developed melanomas after crossing with mice expressing the melanoma oncogene *BRAF^V600E^* [1]; it is important to note that this phenomenon was not observed among albino mice, thus indicating that it is the presence of pheomelanin and not the absence of eumelanin which favors melanoma development [1]. This tumor-promoting effect of pheomelanin seems to be related to the capacity of this melanin type to spontaneously induce reactive oxygen species (ROS) production, even in the absence of UV exposure [1]. Although this peculiar condition is related to melanoma development in individuals with red hair, the incidence of cutaneous melanoma is clearly associated with UV exposure of individuals genetically susceptible to sunlight. In this context, particularly childhood sun exposure represents a risk factor for melanoma development, although adult UV exposure also contributes. Epidemiological data indicate that intermittent, but not chronic, UV exposure represents a risk factor for developing cutaneous melanoma. The contribution of the different components of UV light in the induction of cutaneous melanoma remains to be carefully defined. However, a recent study suggested that the mechanisms through which UVA (320–400 nm) and UVB (280–320 nm) induce melanoma development is different: in fact, UVA induction of melanoma requires the presence of melanin pigment and is associated with DNA oxidative damage, while UVB initiates melanoma in a pigment-independent manner associated with direct UVB DNA damage [2].

## 2. Melanocyte Development

Melanocytes are pigment-producing cells that protect skin epidermis from UV damage and give color to the skin. The function of melanocytes is related to their synthesis of melanin, a pigment displaying two important biological functions, related to the capacity to act both as an oxidant scavenger and as a system absorbing UV and protecting neighboring cells from DNA damage induced by DNA irradiation. Melanocytes originate from the neural crest and migrate through the dermis and epidermis to become located in the hair follicles and in the interfollicular epidermis (in mouse, melanocytes are located only in hair follicles). The neural crest is a transient anatomical structure which develops during embryonic life and gives rise to multiple cell lineages, including neural cells, mesenchymal cells, and melanocytes. Particularly, melanocytes are either originated directly from neural crest cells migrating at the level of the skin through a dorsolateral migratory pathway, or alternatively from Schwann cell progenitors present in the peripheral nerves located at the level of the skin. The differentiation of melanocytes from neural crest cells is controlled through complex molecular mechanisms mediated by a network of transcription factors, including microphtalmia-associated transcription factor (MITF), SOX10, Pax3; the expression of these transcription factors is controlled by some extracellular signaling pathways, including Wingless-type (Wnt) (reviewed in [3]). Among these transcription factors, a key role is played by the basic helix-loop-helix-zipper transcription factor MITF, which is required for the specification of all melanocytes and drives the expression of many genes required for melanogenesis. 

The progenitor cells that generate melanocytes (melanocyte stem cells) are located at the level of the bulge of hair follicles, where are also present in cytokeratin 15^+^ epithelial stem cells. Hair follicles undergo cyclical periods of growth (anagen) and rest (telogen), driven by the coordinated proliferation and differentiation of epidermal and melanocyte stem cells. At the initiation of a new anagen phase, undifferentiated melanocyte stem cells repopulate the bulb through their differentiation into melanocyte precursors that produce melanin pigments and transfer it to adjacent epithelial cells differentiating into hair. During the telogen phase, differentiated melanocytes undergo apoptosis. The Wnt/β-catenin pathway plays an essential role in the development of hair follicles, and is essential for epithelial stem cells. Wnt/β-catenin signaling promotes hair follicle formation; furthermore, the activation of this pathway at the level of epithelial stem cells is of fundamental importance to sustain the proliferation of these cells and to permit hair follicle regeneration during anagen. NOTCH and transforming growth factor (TGF)-β signaling are essential for mesenchymal stem cells’ development and maintenance. Particularly, TGF-β is essential for the induction of melanocyte stem cell quiescence at the level of stem cell niches present in the hair bulge [4]. TGF-β signaling activated through TGF-β type II receptor present on melanocyte stem cells maintains the immaturity and quiescence of melanocyte stem cells [4]. 

In addition to these two pathways, Wnt/β-catenin signaling plays a key role in the induction and maintenance of melanocyte stem cells. Thus, it was shown that Wnt/β-catenin controls multiple steps of neural crest development, ranging from neural crest induction, lineage commitments and differentiation. Conditional β-catenin inactivation in normal crest abolishes melanocyte stem cell induction [5]. During hair regeneration, the activation of Wnt signaling in both epithelial and melanocyte stem cells is essential for coordinated hair repair. In fact, it was shown that both epithelial and melanocyte stem cells activate Wnt signaling at the onset of hair follicle regeneration: (i) the activation of the Wnt/β-catenin signaling pathway into melanocyte stem cells activates their differentiation into differentiated pigment-producing melanocytes; (ii) Wnt activation into epithelial stem cells is essential not only for hair regeneration, but also for the stimulation of melanocyte stem cell proliferation mediated by activated epithelial stem cells through the release of endothelins, acting as growth factors for melanocyte stem cells [6].

In addition to these signaling pathways, the stem cell factor (SCF)/c-Kit pathway also plays an essential role in melanocyte development. Mice with inactivating mutations of c-Kit displayed defects in their pigmentation. Hair shaft progenitors produce SCF and create a niche necessary for the maintenance of differentiated melanocytes and for hair pigmentation [7]. 

It is important to note that melanocytes also originate from neural crest-derived Schwann progenitors located in nerves projecting through the body [8]. Schwann cells and melanocytes share signaling molecules with glial cells, and are differentially regulated by neuregulin and other growth factors [8]. Lineage-tracing experiments have shown that neural crest and Schwann cell progenitor-derived melanocytes are differentially restricted to the epaxial and hypaxial body domains, respectively [8]. The expression of the Forkhead Box d3 (Foxd3) transcription factor regulates the balance between melanocyte and Schwann cell development [8].

## 3. Molecular Abnormalities

### 3.1. Cutaneous Melanoma

Cutaneous melanoma is a highly aggressive tumor of the skin originating from melanocytes (i.e., pigment cells residing in the basal layers of the human epidermis and originating from neural crests during development). Melanoma is certainly the most life-threating tumor of the skin, and is regarded as a major health problem due to the high mortality associated with tumor and to its growing incidence. Cutaneous melanoma is currently classified into four major clinical subtypes: superficial spreading, nodular, acral lentiginous, and *lentigo maligna*, of which the first one is the most common form of cutaneous melanoma. A series of studies carried out by Clark et al. [9] have led to a general model of melanoma development, which provided an important reference in our understanding of the multistep pathogenesis of cutaneous melanoma. According to this model, the first step in melanoma development is represented by the clustering of melanocytes, leading to the formation of benign nevus. The development of cytological atypia within a benign nevus results in the formation of dysplastic nevi (second step). A dysplastic nevus can either regress or develop to a radial growth phase (RGP, third step) melanoma; this last one can then progress into a more aggressive vertical growth phase (VGP, fourth step) melanoma. However, it is important to note that not all melanomas pass through each of these individual steps, and RGP and VGP melanomas can develop directly from transformed melanocytes or nevi [10]. The final step in melanoma development is represented by the formation of local or distant metastases.

Recently, a molecular classification of melanoma was proposed, and this represents an important development in melanoma research for the identification of possible therapeutic targets. Thus, mutually exclusive oncogenic mutations in melanoma involving B Rapidly Accelerated Fibrosarcoma (BRAF) (about 50%), Neuroblastoma Rapidly Accelerated Sarcoma (NRAS) (about 15–20%), c-Kit (about 2%), and Guanine Nucleotide -binding Protein G(q) subunit alpha/Guanine Nucleotide -binding Protein G subunit alpha 11 (GNAQ/GNA11) (about 50% of uveal melanoma) have been identified [11]. According to the different types of molecular abnormalities, eight different molecular subtypes of melanoma have been identified [12]: (a) subtype 1 harbors aberrations in the mitogen-activated protein kinase (MAPK) pathway either by itself or in combination with other pathways, such as the AKT/PI3K and cyclin-dependent kinase (CDK) pathways. Within this subtype, subgroup 1.1 is characterized by mutations of the gene *BRAF* (*BRAF*, a member of the RAF kinase family is the gene most frequently mutated in melanoma) and the other subtypes by *BRAF* mutations in association with abnormalities of the AKT/PI3K pathways; (b) subtype 2 is characterized at molecular level by mutations of c-Kit; (c) subtype 3 is characterized by mutations in two G proteins: GNAQ and GNA11. GNAQ encodes the q subunit of a guanosine triphosphate (GTP)-binding protein and is frequently mutated in malignant blue nevi and ocular melanoma of the uvea. *GNA11* encodes a q subunit of a GTP binding protein and is mutated in uveal melanoma; (d) subtype 4 is characterized by abnormalities of the *RAS* gene encoding a small GTPase. Particularly, in this melanoma subtype *NRAS* mutations are observed; (e) subtype 5 is characterized by molecular abnormalities in molecules controlling melanocyte differentiation; (f) subtype 6 is characterized by abnormalities in the AKT/PI3K signaling pathway that lead to constitutive activation of AKT or Phosphoinositol 3-kinase (PI3K); (g) subtype 7 is characterized by different types of abnormalities at the level of various molecules involved in the G1/S Cyclin/CDK machinery; (h) subtype 8 is characterized by abnormalities at the level of the intrinsic apoptotic machinery, consisting of *p53* mutations and B-cell lymphoma 2 (*BCL2*) overexpression. It is of interest to note that aberrant activity of the mitogen-activated protein kinase (MAPK) signaling pathway represents a critical key factor in the initiation and development of cutaneous melanoma. In line with this finding, extracellular signal-regulated kinase (ERK)—a downstream of MAPK signaling—was shown to be hyperactivated in about 90% of melanomas.

The identification of the *BRAF* mutations has stimulated many experimental studies focused on defining the role of this frequent alteration in melanoma development. Mouse models of *BRAF^V600E^* develop melanoma only after a long latency and with incomplete penetrance, thus suggesting that additional mutations are required for the development of a melanoma malignancy. In these mouse models, melanoma formation—driven by *BRAF^V600E^*—is considerably enhanced by cooperating mutations such as *NRAS* or *PTEN*. These two models have been explored in detail, providing some interesting data. The analysis of the *BRAF^V600E^*/*NRAS* melanoma mouse model showed an unexpected finding related to the effect of BRAF inhibitors in these cells: in fact, BRAF inhibitors induce RAS-dependent binding of BRAF to CRAF, consequent activation first of CRAF and then of MEK-ERK signaling [13]. This phenomenon occurs only in cells that have a concomitantly expressed oncogenic BRAF and an oncogenic NRAS [13]. In an additional experiment, it was shown that a BRAF mutant devoid of kinase activity mimics the effect of BRAF inhibitors, and therefore, the kinase-dead BRAF mutant cooperates with oncogenic NRAS to induce melanoma in mice [13]. These observations have great implications both for our understanding of the pathogenic mechanisms of melanoma progression and for the understanding of the paradoxical complexities of signaling regulation in cancer cells. On the other hand, in mice, the complete or partial loss of Phosphatase and Tensin Homolog (*PTEN*) dramatically accelerates BRAF^V600E^-induced melanoma, thus suggesting the oncogenic potential of combined activation of both MAPK and PI3K/AKT signaling. The analysis of this mouse model provided evidence that the oncogenic effects of BRAF/PTEN mutants is at least in part mediated through activation of the Wnt signaling pathway: this conclusion was reached through the experimental demonstration that β-catenin loss inhibits melanoma formation in BRAF/PTEN-driven melanomas, while β-catenin stabilization accelerates BRAF/PTEN-driven melanomagenesis [14]. It is of interest to note that the tumors developed in animals with β-catenin stabilization are highly metastatic and resemble a subset of aggressive human melanomas [14].

The identification of these different molecular abnormalities in melanoma has important implications for the development of specific target therapies. In fact, the discovery of BRAF mutations occurring in about 50% of melanoma patients encouraged the use of a BRAF inhibitor in melanoma patients carrying the BRAF^V600E^ mutation: the results obtained in various clinical studies (phase I, II, and III trials) have supported a clinical activity of vemurafenib, a BRAF inhibitor. Particularly, a recent phase III study with a follow-up of about 20 months showed an overall improved survival in melanoma BRAF^V600E^ patients undergoing therapy with vemurafenib, compared to the survival observed in patients undergoing standard therapy [15]. It is important to note that 53% of these patients responded to therapy with the BRAF inhibitor and displayed a median overall survival (OS) of about 16 months [15]. Interestingly, the BRAF mutations also occur at high frequencies (>80%) in melanocytic nevi and dysplastic nevi (about 60%) (Table 1). This important observation suggests that BRAF mutation occurs early in melanomagenesis. Since melanocytic nevi rarely progress into melanoma, it is reasonable to conclude that BRAF^V600E^ mutation per se is not sufficient to cause the development of melanoma, and that additional mutations or molecular alterations are required to promote melanoma progression. It is of interest to note that in melanoma patients treated with the BRAF inhibitor vemurafenib, pre-existing BRAF^V600E^ mutant melanocytic nevi either remained unchanged, regressed, or exhibited increased pigmentation. This dichotomy of response to the BRAF inhibitor may be related to the BRAF mutational status: the evolving naevi were BRAF^V600E^ mutated, while the stable naevi were BRAF wild-type [16]. This phenomenon may be related to decreased MAPK activity due to BRAF inhibition. Some BRAF^V600E^ inhibitor-treated patients displayed increased size and pigmentation in some naevi and the development of new BRAF wild-type melanomas, driven by paradoxical MAPK activation elicited by vemurafenib [17].

Similar rates of BRAF mutations are present in primary and metastatic melanomas, as well as in melanoma cell lines, suggesting that BRAF mutations occur before tumor progression and dissemination and their incidence remains constant during tumor progression. A recent study has investigated the prevalence of pathogenic mutations in *BRAF* and *NRAS* genes in primary and metastatic melanoma tissues. BRAF/NRAS mutations exhibited only a slightly increased prevalence in metastatic (63%), compared with primary site samples (58%) [18]. The paired analysis of primary tumor and metastases showed the existence of discrepancies in *BRAF/NRAS* mutation patterns: interestingly, the highest frequency of these discrepancies was observed in patients with cerebral and subcutaneous metastases [18]; in half of the discrepant cases, a wild-type primary tumor and a mutated metastasis was observed. This finding represents a precious indication that in a portion of melanoma patients, mutations in *BRAF* and *NRAS* genes occur during disease progression [18]. A very intriguing finding of this study was that some cases of wild-type metastases have been observed in cases with mutated primary tumors; furthermore, other cases displayed a different mutation pattern between primary and secondary tumors (i.e., mutations of *BRAF* in primary tumors and mutations of *NRAS* in secondary tumors) [18]. It is of interest to note that the discrepancy between primary and metastatic lesions in *BRAF*/*NRAS* mutational status was higher for brain and skin metastases than for lymph node and visceral metastases [19]. At variance with *BRAF*/*NRAS*, *p16CDKN2A* gene mutations were more frequent among metastases (14%) than in primary tumors (7%) [18]. These observations add further evidence in favor of the existence of great molecular heterogeneity in melanoma within a tumor cell population of a single patient [18]. This cellular mutational heterogeneity was also observed in other studies. Thus, Lin and coworkers have examined *BRAF* mutations in sets of single cells isolated from acquired melanocytic nevi, and observed a consistent number of nevus cells that contained wild-type BRAF, mixed with nevus cells that contained BRAF^V600E^ [20]. In another study carried out by the same authors, single cell mutation analysis showed that most primary melanomas contained both BRAF-WT and BRAF-mutant cells [21]. Thus, the analysis of different lesions in the same patient clearly indicates that in many cases cells with mutant BRAF coexist with cancer cells lacking *BRAF* mutations; the BRAF wild-type tumor cells are completely unaffected by targeted BRAF treatment, and thus greatly contribute to drug resistance [22]. Differences in the mutational status of *BRAF* were observed in 44% of cases [23].

The polyclonality of BRAF mutations support the idea that a BRAF mutation is not a founder event during melanoma development, but one of the multiple clonal events occurring in this neoplasia and related to disease progression. It is important to note that response to treatment of melanoma BRAF^V600E^ tumors to RAF inhibitors is often followed by recurrence activation of MAPK. Resistance to RAF inhibitors occurs through different molecular mechanisms that have only been partly elucidated. Particularly, these mechanisms involve the emergence of mutant BRAF-concurrent RAS or MEK mutations, and mutant BRAF amplification or alternative splicing or PI3K-AKT-upregulating genetic alterations. In some patients, the loss of stromal antigen 2 (STAG2) or STAG3—encoding subunits of the cohesion complex—results in resistance to BRAF inhibitors [24]. Given this situation, it seemed important to evaluate the effect of MAPK inhibitors in these patients—particularly downstream inhibitors of this pathway. In this context, both studies on experimental models [25] and in metastatic melanoma patients [26] support the antitumor activity of MEK inhibitors in BRAF^V600E^ melanomas. Particularly, combined treatment with a BRAF inhibitor and a MEK inhibitor increased progression-free survival, objective response, and duration of response as compared with the BRAF inhibitor alone [26]. In spite of this initial response, resistance developed in most patients after an average of 9.4 months. The mechanisms of resistance to combined RAF/MEK inhibition have been recently explored, showing the occurrence of novel activating MEK2 mutations (MEK2 Q60P) selectively observed only in resistant tumors [26]. The continued MAPK signaling-based resistance observed in these patients has suggested that either alternative dosing of current agents, new more potent MEK and BRAF inhibitors, or inhibition of the downstream ERK kinase could be used to try to obtain more durable responses [27]. During BRAF inhibition therapy, the emergence of cutaneous squamous cell carcinomas was frequently observed and has been associated with paradoxical MAPK pathway activation. Melanoma patients receiving the combination therapy with a BRAF inhibitor and a MEK inhibitor had a reduced incidence of secondary squamous cell carcinomas [28]. In addition to squamous cell carcinomas, myelomonocytic leukemia and chronic lymphocytic leukemias have also been observed among melanoma patients undergoing therapy with BRAF inhibitors.

Reactivation of MAPK—mostly in the form of additional *NRAS* or Kirsten Rapidly Accelerating Sarcoma (KRAS) mutations—is the most frequent mechanism of resistance to BRAF inhibitors [29,30]. It is commonly believed that BRAF and NRAS mutations are mutually exclusive in single cells due to self-induced apoptosis related to hyper-activation of the MAPK pathway: consequently, resistant tumors of patients treated with BRAF inhibitors may be composed of mixtures of mutually exclusive subclones. However, recent studies showed that BRAF and NRAS mutations can co-occur in some melanoma cells of BRAF-resistant patients, and their presence is associated with a heterogeneous and variable response to different types of inhibitors of MEK, ERK, PI3K, and AKT [31].

Some strategies have been proposed to bypass or to mitigate the chemoresistance to BRAF inhibitors displayed by BRAF-mutated melanomas. These strategies have been developed in the context of peculiar methodologies of study of the development of chemoresistance. Thus, Fallhi-Sachani and coworkers have used live-cell imaging, single-cell analysis, and molecular profiling to demonstrate that exposure of melanoma cells to RAF/MEK inhibitors elicits a heterogeneous response in which some cells die, some arrest, and the remaining progressively adapt to drug [32]. Xue and coworkers used the model of patient-derived xenografts to investigate the development of drug resistance linked to BRAF^ampl^ [33]. Sequential monotherapy with BRAF, MEK, and ERK inhibitors was ineffective to prevent BRAF^ampl^ development; concurrent treatment with these inhibitors delayed the occurrence of BRAF^ampl^; intermittent treatment with this combination of drugs prevents BRAF^ampl^, and is able to inhibit tumor proliferation in 100% of cases [33].

The introduction of targeted BRAF inhibition and combined BRAF and MEK inhibition therapies have improved the clinical outcomes for patients with metastatic BRAF-mutated melanomas. Unfortunately, the efficiency of these treatments is greatly limited by the occurrence of drug resistance. As outlined above, drug resistance to BRAF inhibitors is mainly related to ERK reactivation, and less frequently to upregulation of mTOR and Wnt/β-catenin pathways and modulation of apoptosis. However, the molecular mechanisms mediating resistance to combined treatment with BRAF inhibitors and MEK inhibitors are less understood. A recent study partly clarified these mechanisms. The analysis of clinical samples and of double-resistant leukemic cell lines provided evidence that p21-activated kinases (PAKs) become activated in cells with acquired combined resistance [34]. Interestingly, PAKs are able to induce resistance to BRAF inhibitors by a mechanism involving CRAF and MEK phosphorylation, with consequent ERK reactivation, and are able to induce resistance to combined BRAF and MEK inhibitors by a mechanism dependent upon the induction of Janus Kinase (JNK) and β-catenin phosphorylation and mammalian Target of Rapamycin (mTOR) pathway activation, thus bypassing ERK [34]. These observations have fundamental implications for the development of specific therapies aiming to bypass resistance to BRAF and MEK inhibitors [34].

The occurrence of tumor genetic heterogeneity represents a major challenge in the development of curative therapeutic treatments for metastatic melanoma [35]. Recent studies at the level of single melanoma cells illustrate the dramatic complexity of tumor heterogeneity. Thus, Tirosh et al. have used this technique to analyze 19 melanoma patients, showing the existence of a consistent heterogeneity at the level of both tumor cells and the tumor microenvironment (endothelial cells, immune infiltrating cells) [36]. The tumor cells displayed transcriptional heterogeneity (i.e., tumors characterized by high levels of the MITF transcription factor also contained cells with low MITF and elevated AXL kinase) associated with the cell cycle, spatial context, and a drug-resistance program [36].

The PIK3CA gene was found to be mutated in about 10% of melanomas, and these mutations co-existed with BRAF/NRAS mutations in about 10% of BRAF/NRAS mutant melanomas; on the other hand, about 50% of PIK3CA-mutant melanomas displayed BRAF/NRAS mutations [37]. 

In addition to BRAF^V600E^, recent studies have shown the existence of other frequent mutations occurring in melanoma that could be therapeutically targeted. Analyses of whole-genome sequence data have led to the identification of Phosphatidylinositol Triphosphate Dependent Rac Exchange Factor 2 (PREX2), a PTEN-interacting protein and negative regulator of PTEN, as a frequently mutated (14% of cases) gene in human melanomas [38]. The frequency of *PREX2* mutations was markedly higher in metastatic melanomas (about 45%) [38]. These mutations are biologically relevant because an ectopic expression of mutant *PREX2* accelerated tumor formation of immortalized human melanocytes in vivo [38]. Furthermore, *MEK1* and *MEK2* mutations have been detected in 8% of melanoma patients [38]. These mutations result in constitutive ERK phosphorylation and higher resistance to MEK inhibitors; the presence of MEK1 or MEK2 mutations did not seem to correlate with BRAF or NRAS mutation status [39]. Another study has shown almost mutually exclusive mutations of *MAP3K5* and *MAP3K9* occurring in approximately 24% of melanomas, which occur independently of activating mutations in *BRAF* and *NRAS* [40]. These mutations cause an abrogation of the signaling pathways controlled by these two kinases—an event relevant for melanoma development [40]. Whole-exome sequencing of 14 melanomas has led to the identification of other frequently mutated genes: among them, two were not reported in other studies [41]. The Transformation/Transcription Domain Associated Protein (TRRAP) gene, encoding the transformation/transcription domain-associated protein, was found to be mutated in 4% of melanomas [41]; the Glutamate Ionotropic Receptor NMDA Type Subunit 2A (*GRIN2A*) gene, encoding a glutamate receptor subunit epsilon-1, was found to be very frequently mutated (in approximately 25% of cases) [41].

The histone methyltransferase SETDB, pertaining to the family of SET domain histone methyltransferases and involved in the methylation of histone 3 on lysine 9 (H3K9), was found to be recurrently amplified in melanoma patients [42]. Using a zebrafish melanoma model, evidence was provided that SET Domain B1 (SETDB1) cooperates with BRAF^V600E^ to induce melanoma formation [42]. *SETDB1* overexpression determines gene expression dysregulation of several genes, including *HOX* genes [42]. The SETDB1 gene is located on chromosome 1 at the level of the 1q21.3 region—a locus region with ten genes: this locus was identified as a melanoma susceptibility locus and contains two plausible genes for melanoma susceptibility: *SETDB1* and Aryl Hydrocarbon Receptor Nuclear Translocator (*ARNT*) [43]. Immunohistochemistry studies have shown that overexpression of SETDB1 was observed in 57% of patients: SETDB1 overexpressing tumors display an aggressive tumor behavior [43]. Interestingly, in 21% of melanomas the overexpression of another H3K9 methyltransferase—EHMT2—was also observed [44].

Mutation analysis of protein tyrosine kinases identified some recurrent mutations in *ERBB4* (19% of melanomas) and Fms related Tyrosine Kinase 1 (*FLT1*) and Protein Tyrosine Kinase 2 Beta (*PTK2B*) (10% of melanomas) [45]. Missense mutations occurring at the level of the *ERBB4* gene determine increased kinase activity and tumor transformation capacity [45]. In *ERBB4*-mutant melanomas, mutant *ERBB4* expression is required to sustain melanoma growth [45].

A recent study identified the recurrent (4% of melanomas) mutation of the Required for Cell Differentiation 1 (*RQCD1*) (required for cell differentiation 1 homolog) gene in human melanomas [46]; this gene belongs to the CCR4-NOT complex and represents the first example of a gene of this complex to be mutated in cancer [46]. The *RQCD1*-mutant tumors were associated with head and neck and upper limb location, lentigo maligna melanoma subtype and BRAF^V600K^, but not BRAF^V600E^ or *NRAS* mutations [46].

UV radiation is strongly associated with an increased risk of developing melanoma. Therefore, several studies have screened the presence of signature UV mutations (pyrimidine dimers) at the level of the various oncogenes mutated in melanoma. However, surprisingly, pyrimidine dimers are identified less frequently within mutated oncogenes in melanomas than in many other tumors. Whole genome sequencing of a human melanoma, compared to normal non-tumoral tissue of the same patient, unequivocally identified thousands of mutations, many of which were pyrimidine dimers, strongly suggesting that these mutations were related to UV exposure [47]. However, not many of this type of mutation were identified at the level of driver mutations, while they were much more frequent at the level of “passenger mutations”. 

**Figure 1:**
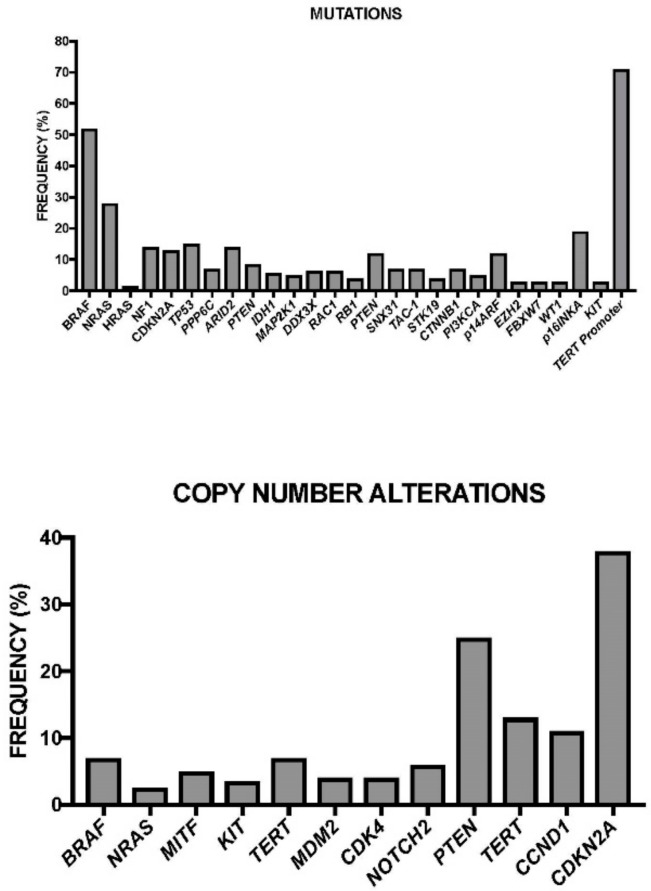
Recurrent genetic alterations (mutations and copy number alterations) observed in cutaneous melanomas.

The identification of these new recurrent genetic mutations in melanomas and the lack of identified driver mutations in the melanoma subtypes lacking *BRAF* and *NRAS* mutations indicate that our understanding of the genetic alterations driving this malignancy remains incomplete. To try to bypass this gap, Hodis et al. have recently reported whole-exome sequencing data from 121 melanoma/normal pairs, using a statistical approach enabling positive selection at each gene locus based on exon/intron mutational distributions, and the predicted functional impact of each mutation. This approach allowed the discovery of new functionally relevant gene mutations directly related to UV mutagenesis [48]. This approach allowed the identification of 11 genes harboring a significant functional mutation: six of them (*BRAF*, *NRAS*, *TP53*, *PTEN*, *CDKN2A*, and *MAP2K1*) were already-known melanoma-associated genes; five of them (PPP6C, RAC1, SNX31, TACC1, and STK19) were newly identified (Figure 1) [48]. *PPP6C* encodes for the catalytic subunit of PP6 protein phosphatase complex, acting as a negative regulator of the melanoma oncogene *CCND1* and as the major phosphatase of the Aurora kinase, and was found to be mutated in 9% of melanoma samples. STK19 encodes a predicted kinase of unknown function and was found to be mutated in 5% of cases: its mutations exhibited a hot spot pattern. *TACCa* (encoding transforming acidic coiled-coil protein 1, a stimulator of Ras and PI3K pathways) and *SNX31* (encoding the poorly characterized sorting nexin 31, probably acting as a Ras effector protein) mutations exhibited a distributed pattern of mutational events. RAC1, encoding a RAS-related member of the Rho superfamily of GTPases, was mutated in 5% of melanomas [48]. A very recent study confirmed the frequent occurrence of *PPP6C* and *RAC1* mutations in melanoma. This study was carried out in melanomas related to sun exposure. This study showed that in these patients the *PPP6C* mutations are frequent, occurring in 12% of sun-exposed melanomas, exclusively in tumors with mutations in *BRAF* or *NRAS* [49]. In addition, *RAC1* mutations have been observed in 9.2% of sun-exposed melanomas: these mutations were more frequent in melanomas that were wild-type for both NRAS and BRAF (12.5% of melanomas with wild-type BRAF and NRAS had the mutation compared to 6.2% of melanomas with mutant BRAF or NRAS). The mutation of *RAC1* occurs at the level of the Pro29 to Ser in a highly-conserved switch domain and causes a conformational change of the protein, with consequent increased binding of the protein to the downstream effectors; importantly, this mutation causes increased melanocyte proliferation [49].

A recent study provided evidence of the recurrent mutations of the *FBXW7* gene in human melanomas; in this context, it is important to point out that NOTCH1 is a substrate of this gene [50]. Thus, Aydin and coworkers have found this gene to be mutated in 8% of melanoma patients: *FBXW7* mutations determine an inactivation of the encoded protein and a consequent accumulation of its substrate NOTCH1 [50]. Protein expression analysis of tumor samples showed that *FBXW7* inactivation is a frequent event occurring in about 40% of cases. Functional experiments based on the silencing of FBXW7 in immortalized melanocytes showed an accelerated tumor formation in vivo and enhanced NOTCH1 expression [50]. FBXW7 coordinates the ubiquitin-dependent proteolysis of a number of key cellular regulators, and through this mechanism, controls processes essential for cellular physiology, such as cell proliferation, differentiation, and survival [51]. Interestingly, F-Box and WD Repeat Domain Containing 7 (FBXW7) is one of the most frequently mutated genes in human cancers [51]. Recent studies in various animal models have additionally supported a role for reduced FBXW7 expression in melanoma tumorigenesis. In fact, the reduced FBXW7 expression observed in some melanomas stabilizes the heat-shock factor 1, inducing a cell response and stimulating the metastatic potential of melanoma cells [52]. Furthermore, *FBXW7* inactivation in BRAF^V600E^-driven mouse model leads to melanoma development, not associated with an aggressive phenotype [53].

Considering all the genetic alterations occurring in melanomas a genetic landscape focused on the driver and secondary recurrent mutations occurring in these tumors was proposed [48]. According to this global evaluation, three groups of melanomas can be tentatively identified: (a) a large group characterized by the occurrence of *NRAS* or *BRAF* mutations, these two mutations being mutually exclusive (very rare cases of co-occurring mutations harbored either a non-oncogenic *NRAS* mutation with an activating *BRAF* mutation or a non-V600 *BRAF* mutation with an oncogenic NRAS mutation); a high percentage of these melanomas harbored a *PTEN* focal deletion or mutation; Cyclin-Dependent Kinase Inhibitor 2A (CDKN2A) is a melanoma tumor suppressor gene encoding two tumor suppressor proteins through alternative splicing: p16^INK4a^ and p14^ARF^, the first acting as a cyclin-dependent kinase inhibitor and the second inhibiting Mouse Double minute 2 homolog (MDM2), and through this mechanism, activating p53) copy losses are also frequent; finally, point mutations in some genes (e.g., PPP6C) are also frequent. (b) A second group is characterized by the presence of wild-type NRAS and BRAF, and by a high number of copy number gains and a low mutational load; the copy number gain occurs at the level of chromosomes 5p13 (*RICTOR*), 11q13 (*CCND1*), 12q14 (*CDK4*). (c) A third group of melanomas includes tumors with wild-type NRAS and BRAF, few copy number alterations, and a high number of mutations; among the frequent mutations are those occurring at the level of the gene *NF1* (these are inactivating mutations, with possible oncogenic implications given the capacity of NF1 to act as a negative regulator of RAS signaling) and of the tumor suppressors *p53*, *ARID2*, and *PTPRK* and of the *RAC1* gene.

Lovly and coworkers have developed a melanoma-specific multiplex mutational profiling assay to detect the 43 recurrent mutations occurring in 6 genes frequently mutated in melanomas: *BRAF*, *NRAS*, *KIT*, *GNAQ*, and *GNA11* [54]. Using this approach, they have observed that about one-third of melanomas lack any of these mutations and have been defined as “pan negative” [54]. Consistent efforts have been made to molecularly define these “pan-negative” tumors. Thus, it was observed that 8% of “pan-negative” melanomas display non-V600 exon 15 *BRAF* mutations [55]; 4–8% contain activating BRAF fusions, both of which are sensitive to MEK inhibition [56]. Xia and coworkers have recently performed a meta-analysis of somatic mutations from next-generation sequencing data of melanomas. This analysis showed that: (a) BRAF^V600^ mutations occurred in 50.2% of cases and TP53 and COL1A1 mutations co-occurred in these tumors; (b) *NRAS* mutations occurred in 19.5% of cases and *PPP6C*, *KALRN*, *PI3K3R4*, *TRPM6*, *GUCY2C*, and *PRKAA2* mutations co-occurred in these tumors; (c) *GNA11* and *Kit* mutations occurred in 1.2% and 0.4% of cases, respectively; (d) 28.7% of the tumors can be defined as “pan-negative” tumors [57]. Particular attention was focused on analyzing the mutational spectrum of pan-negative melanomas, which included: (i) less-frequent (different from those commonly observed) mutations of *BRAF, NRAS, KIT, GNA11*, and *GNAQ* (globally occurring in about 25% of these patients); (ii) 12 genes were found to be mutated in “pan negative” tumors at a frequency significantly higher than that observed in driver gene-positive tumors, and these genes include *ALK, STK 31*, *DGKI*, *RAC1*, *EPHA4*, *ADAMTS18*, *EPHA7*, *ERBB4, TAF1L*, *NF1*, *SYK*, and *KDR* (some of these genes displayed a recurrent mutation observed in several pan-negative tumors) [57]. Another recent study showed several important findings about “pan-negative” melanomas: these tumors are a complex group of melanomas, more commonly occurring in sun-exposed body sites, associated with pronounced solar elastosis [58]. In line with this finding, these tumors have frequent C > T transitions and dinucleotide CC > TT transitions—typical DNA abnormalities induced by UV-mediated damage [58]. These melanomas have a mutational load significantly higher than BRAF or NRAS-mutated melanomas [58]. Finally, these tumors display a wide spectrum of mutations involving several major pathways, including MAPK, cell-cycle, c-kit, p53, PI3K/AKT, NF1, and NOTCH [58]. According to these findings, it was concluded that “pan-negative” melanomas represent a spectrum of molecular subtypes [58]. 

It is important to note that while the discovery of *BRAF* mutations and the development of potent RAF inhibitors has revolutionized the therapy of this melanoma subtype, NRAS-mutant melanoma remains without an effective pharmacological therapy. Pharmacological inhibition of NRAS has been largely unsuccessful. Recently, alternative experimental approaches have been identified to inhibit NRAS-mutated melanomas: particularly, it was shown in experimental models of NRAS-mutated melanomas that the combined inhibition of MEK and CDK4 induced apoptosis and inhibited tumor cell proliferation [59]. As mentioned above, the PI3K signaling pathway is frequently activated in human melanomas. Elevated PI3K/AKT pathway activity was observed in about 17% of benign nevi, 43% of dysplastic nevi, 49% of primary melanomas, and 77% of metastatic melanomas [60]. As mentioned above, *PI3KCA* mutations are rare in melanomas, while the loss of PTEN function (as a consequence of mutations, loss of heterozygosity, chromosomal loss, microRNA-dependent repression of PTEN synthesis, and transcriptional silencing induced by methylation) is frequent in melanoma. Somatic PTEN alterations were identified in 14% of specimens in the TCGA melanoma cohort, including both focal deletions (6%) and mutations (8%). *PTEN* loss and *BRAF* mutation are frequent in melanoma, and these two events may cooperate in the transformation of melanocytes: in fact, it was shown that PTEN loss abrogates BRAF^V600E^-induced senescence in human melanocytes and promotes tumor development [61]. The occurrence of this tumorigenic mechanism was demonstrated in melanomas relative to adjacent nevi [61]. The pharmacologic inhibition of PI3K together with BRAF in melanomas resulted in a synergistic and potent antitumor effect [61].

A recent study provided evidence about a very peculiar molecular mechanism through which there is a loss of PTEN expression in melanoma cells. In fact, it was shown that in melanoma PTEN expression is downmodulated through competitive endogenous RNAs (ceRNAs): these RNAs sequester microRNAs to regulate mRNA transcripts containing common microRNA recognition elements. Karreth et al. provided evidence that PTEN expression is regulated by ceRNA activity [62]. Particularly, it was shown that Zinc finger E-box-binding homeobox 2 (*ZEB2*) modulates PTEN protein levels in a microRNA-dependent, protein coding-independent manner: ZEB2 downmodulation is commonly observed in human melanomas, and leads to an increased binding of some microRNAs to PTEN, with a consequent decrease of PTEN protein levels [62]. In murine mouse melanoma models, ZEB2 expression cooperates with BRAF^V600E^ to promote melanogenesis [62]. In acral melanomas there is a relationship between *PTEN* loss and amplification of the AMPK-related kinase NUAK2; importantly, in these melanomas, the presence of both *PTEN* loss and *NUAK2* amplification was associated with poor prognosis [63]. 

Particularly interesting are the observations derived from the analysis of c-kit mutations in various types of melanomas. As mentioned above, c-kit mutations are relatively rare (about 2%) in cutaneous melanoma. However, their frequency was markedly higher (23%) in acral melanomas (acral lentiginous melanoma is a peculiar type of melanoma occurring in non-hairing surfaces of the body such as the palms, soles, and under the nails, and its incidence is not related to sun exposure), mucosal melanomas (15.6%) and conjunctival melanomas (7.1%) [64]. Increased *Kit* copy number was observed in 27% of acral melanomas, 26% of mucosal melanomas, and 7% of cutaneous melanomas [64]. *Kit* mutations do not overlap with *BRAF* and *NRAS* mutations [64]. The overexpression of *Kit* was observed in 80% of primary vaginal melanomas [65]. Asian populations are more prone than Caucasian populations to develop acral and mucosal than cutaneous melanomas. Therefore, it was of some interest to evaluate the frequency of *Kit* mutations in melanomas of a Chinese population. This analysis showed that the frequency of Kit mutations was about 12% in acral melanoma and 9.6% in mucosal melanoma, which are lower than the mutation frequency reported in Caucasian patients. In contrast, the frequency of kit mutations in cutaneous melanomas (about 21%) is markedly higher than that observed in Caucasian patients [66]. *Kit* mutations were found to be associated with a significantly shortened survival time among stage III and stage IV acral or mucosal melanoma patients [67]. These *Kit* mutations determine constitutive activation of c-kit, resulting in downstream activation of the MAPK and PI3K-AKT pathways, inducing cell proliferation and enhanced survival. The role of mutant c-kit in sustaining melanoma proliferation is directly supported by the observation that tyrosine kinase inhibitor imatinib decreased melanoma cell proliferation and induced apoptosis of these tumor cells. These observations have stimulated the development of clinical trials based on the use of agents targeting c-kit, such as imatinib, sunitinib, nilotinib, and dasatinib. The therapeutical results obtained with some of these agents in the context of phase I/II studies are now available, and indicate that patients with kit mutations are more responsive to treatment with tyrosine kinase inhibitors than patients with kit amplification or overexpression [67]. 

It is of interest to note that Kit expression is low or undetectable in cutaneous melanomas displaying BRAF or NRAS mutations. The loss of Kit expression in these melanomas was attributed to frequent deletion or silencing by hypermethylation of the *Kit* locus [68]. In animal models of melanoma formation driven by BRAF^V600E^ and mutated *TP53* (loss-of-function mutation), kit loss favors melanoma formation, enhancing RAS/MAPK signaling activation [69]. According to these observations, it was concluded that in BRAF-mutated melanomas, a normal kit expression/function could dampen oncogenic signaling from mutated *BRAF* [69]. 

Alterations of RAS pathway members are also very frequent in acral melanomas, being detected in 87% of these patients: *NRAS* (17%), Aurora Kinase A (*AURKA*) (37.5%), Ciclin D1 (*CCND1*) or telomerase reverse transcriptase (TERT, 31%), and *RAS* (25%) [70]. A recent study provided an integrated genomic analysis of acral melanoma based on a detailed genetic analysis carried out on 34 acral melanomas. The mutational load of these tumors is low, with a median of 42 mutations/tumor [71]. Unlike cutaneous melanoma, in acral melanoma somatic alterations were dominated by structural variation and absence of UV-derived mutation signatures [71]. Only 38% of these patients demonstrated driver *BRAF/NRAS/NF1* mutations [69]. In 41% of acral melanoma patients, TERT aberrations were observed, encompassing point mutations, breakpoints, copy gains, and coding germline mutations [69]. All acral melanomas displaying TERT copy number gains were also all BRAF wild type, but overlapped with N/KRAS and NF1 alterations [71]. All patients with TERT alterations showed TERT expression, and TERT inhibition in these cells resulted in decreased viability [72]. These observations are particularly interesting, and may offer potential for the treatment of these melanomas, based on the use of small drug-like pharmacological molecules of recent identification, acting as hTERT repressor and inducing apoptosis through inhibition of hTERT’s role in regulating apoptosis-related proteins and induction of senescence by decreasing telomerase activity and telomere length [72]. 

The systematic sequencing of melanoma genome identified many mutations at the level of the protein coding sequences, but only very few mutations in gene regulatory regions. Recently, one exception to this rule was observed, showing the very frequent (71% of cases) mutations in two different sequences of the core promoter of *TERT* [73]. These mutations generate de novo consensus binding motifs for Ets transcription factors, leading to increased expression of telomerase reverse transcriptase [73]. These findings were confirmed in a parallel study, where it was observed that the frequency of *TERT* promoter mutations was markedly higher in metastatic tissues (85%) than in the corresponding primary tumor tissues (33%) [74]. However, other studies have failed to show an increased frequency of *TERT* promoter mutations, compared to primary tumors [75]. Apart from mutations in *BRAF* and *NRAS*, recurrent *TERT* promoter mutations were the most frequent genomic alterations [76]. Because of these mutations in the *TERT* promoter region, de novo Ets binding sites are created: these sites bind Ets transcription factors such as ELK1 and ELK4; ELK1 and ELK4 are downstream targets of BRAF, and may represent the link between *BRAF* activating mutations and telomerase activation [74]. These considerations suggest that *TERT* promoter mutations are important driver events and contribute to melanoma tumorigenesis. *TERT* promoter mutations were more frequent in non-acral skin melanomas (50%) than in mucosal (23%) and acral (19%) melanomas [76]. Importantly, the presence of *TERT* promoter mutations was associated with BRAF or NRAS mutations [74]. Importantly, *TERT* promoter mutations are associated with poorer overall survival in patients with non-acral cutaneous melanomas [76]. The negative impact of genetic and epigenetic alterations of TERT were also confirmed in a population of adolescent and young patients with melanoma [77]. Other recent studies have identified recurrent mutations in melanomas at the level of the promoter regions of some genes. Thus, Werhold and coworkers identified a recurrent mutation at the level of the promoter region of the Succinate Dehydrogenase subunit D (*SDHD*) gene: in this case, the mutations disrupted the existing ETS binding sites and decreased the levels of *SDHD* gene expression [78]. SDHD gene promoter mutations are associated with poor prognosis [78]. A recent study explored the mechanistic relevance of RAS-ERK activation in BRAF/NRAS mutant cells with mutant *TERT* promoter. An important role of RAS-ERK signaling is in the maintenance of an active chromatin state at mutant *TERT* promoters, facilitating the recruitment of RNA polymerase II that activates TERT transcription in BRAF-mutant melanoma cells [79]. This observation helps to understand the molecular mechanisms underlying the significant co-association of *BRAF* mutations and *TERT* promoter mutations in melanoma progression [79]. 

The studies on the genetic abnormalities occurring in melanoma are progressively developing a picture of the DNA changes associated with and in large part responsible for the development of this neoplasia. However, studies carried out in recent years have provided initial evidence about a series of epigenetic abnormalities occurring in this tumor. Epigenetic alterations of DNA (DNA methylation), of RNA (non-coding RNAs), histone modifications, chromatin remodeling, and changes in polycomb group proteins have been described in melanoma (reviewed in [12]). There is evidence that these epigenetic changes contribute to melanoma development, but it is still unclear how these different events combine and how they cooperate with genetic changes [12]. Recent studies suggest that some epigenetic changes—such as the loss of 5-hydroxymethylcytosine (5-hmC)—are epigenetic hallmarks of melanoma and play a relevant role in the progression of this neoplasia. Downregulation of the enzymes involved in hmC production—isocitrate dehydrogenase 2 (IDH2) and Ten-eleven Translocation (TET) family enzymes—is a main mechanism responsible for hmC loss in the melanoma epigenome [80]. Genome-wide mapping and comparative analysis of the 5-mC and 5-hmC landscape in benign nevi and primary melanomas indicates that a program of genes involving various cancer pathways display a significant reduction of 5-hmC in comparison between benign nevi and melanomas [80]. This reduction of 5-hmC was reversed by overexpression of active *TET2* [80].

Interestingly, at variance with tumor cells often expressing reduced TET2 levels, increased *TET2* expression was reported in tumor-associated macrophages [81]. The increased *TET2* expression in these cells exerts a tumor-promoting effect, since it sustains the immunosuppressive activity of these cells [81]. Ablation of *TET2* expression in myeloid cells suppresses the growth of melanoma cells in vivo and induces a switch of the gene expression profile of tumor-associated macrophages from an immunosuppressive to a proinflammatory pattern [81]. 

Another important epigenetic target in melanoma is MDM4, also known as MDMx, a negative regulator of p53 function. Although inactivating mutations or allelic loss of p53 are rare in melanomas, the p53 pathway is inactivated in the large majority of melanomas through a mechanism involving overexpression of negative regulators of p53: rarely by overexpression of *MDM2* due to gene amplification, frequently (about 65%) by overexpression of *MDM4* due to post-transcriptional mechanisms [82]. The increased *MDM4* expression in melanoma has pathological implications, as shown by several observations: (a) MDM4 protein expression was either undetectable or very low in normal melanocytes and in benign nevi; (b) *MDM4* overexpression cooperates with NRAS mutation in inducing melanoma development in experimental models; (c) *MDM4* overexpression promotes the survival of melanoma cells and the inhibition of the MDM4–p53 interaction restores p53 function in melanoma cells, resulting in increased sensitivity to standard cytotoxic therapy and to BRAF inhibitors [82]. More recent studies have clarified the molecular mechanisms responsible for *MDM4* overexpression in melanoma, mainly related to an alternative splicing switch. In normal adult melanocytes, a decay-targeted isoform of MDM4 (MDM4-S) is produced; in melanoma cells, enhanced exon 6 inclusion leads to the expression of full-length MDM4 [80]. Interestingly, in human melanoma cell lines and in melanoma patient-derived xenograft mouse models, antisense oligonucleotide-mediated skipping of exon 6 decreased MDM4 levels, inhibited melanoma growth, and enhanced sensitivity to various anti-melanoma therapeutics [83]. Other mechanisms contribute to p53 inactivation in melanoma. Although *MDM2* overexpression due to gene amplification is rare, post-transcriptional mechanisms determine the frequent upregulation of MDM2 expression in melanoma cells. In fact, in these cells miR-18b expression is frequently downmodulated by hypermethylation; MDM2 is a molecular target of miR-18b, and the downmodulation of this miR in melanoma cells is responsible for MDM2 upmodulation, with consequent p53 inactivation [84]. Another mechanism stimulating *MDM2* expression in melanoma cells is related to AXL receptor signaling [85].

Recently, the TCGA Network performed a detailed multiplatform analysis of 333 cutaneous melanomas (20% primary site and 80% metastases, mostly regional metastases), and proposed a genomic classification which is very useful for the understanding of the molecular basis of this tumor and for the development of individualized treatments [86]. This study allowed the identification of four molecular subtypes (Table 2): (a) a *BRAF-mutated subtype* (52% of total), with the majority of spot mutations targeting the V600 amino acid residues (in large part V600E) and, less frequently, the K601 residue (these hot-spot mutations anti-correlated with hot-spot NRAS mutations); *BRAF* non-hot-spot mutations (i.e., exon 11 mutation) are rare, and co-occurred with *RAS* hot-spot and *NF1* mutations; (b) *a RAS-mutated subtype*, the second most frequent subtype, with the most frequent involvement of *NRAS* (18% of total) with a hot-spot mutation mostly at the level of Q61 amino acid residue; less frequent hot-spot mutations occurred at the level of *HRAS* and *KRAS*; all RAS hot-spot mutations were mutually exclusive with *BRAF* mutations; (c) *an NF1-mutated subtype* (14% of total), with frequent loss-of-function mutations (leading to MAPK activation due to the lack of the inhibitory effect of NF1 on RAS activity); *NF1* mutations were frequently (about 39%) observed in non-hot-spot BRAF/RAS melanomas, and particularly those with a UV-signature (about 70%); (d) *a triple wild-type subtype* (about 15% of total), characterized by loss of hot-spot *BRAF*, *RAS*, and *NF1* mutations; some driver mutations were observed in these tumors, including *GNAQ*, *GNA11*, *KIT*, *CTNNB1*, and *EZH2* [86]. Patients with BRAF subtype were younger, while patients with NF1 subtype were older. The number of mutations per tumor was highly variable at the level of the single patients pertaining to the four subtypes; patients with *TP53* mutant melanomas had more mutation counts, and particularly of C > T transitions [86]. The analysis of copy number alterations in the various tumor subtypes provided some interesting findings: Triple-WT had significantly more copy number alterations (CNAs) than the other tumor subtypes, these CNAs involving some known oncogenes such as *Kit, PDGFRA*, *KDR*, *CDK4*, *CCND1*, *MDM2*, and *TERT*; the BRAF mutant subtype was characterized by frequent CNAs of *BRAF*, *MITF*, *PD-1*, and *PD-L1*; *NRAS* mutant subtype was characterized by frequent *NRAS* amplifications [86]. *TERT* promoter mutations were frequent in the BRAF, RAS, and NF1 subtypes (ranging from 72% to 83%), while they are rare in Triple-WT (about 7%); in this last tumor subtype, alternative mechanisms (gene amplification) are involved in *TERT* activation [86]. According to the mutational spectrum, the RAS-MAPK-AKT is the pathway most frequently (91%) activated in melanoma, followed by the RB1/CDKN2A cell-cycle pathway (69%) and MDM2A/TP53 (19%). According to the transcriptional profile of gene expression, three stable clusters of expression were observed: immune, characterized by the expression of an elevated number of genes associated with immune cell subsets, cytokines, chemokines, and immune checkpoint proteins; keratin, characterized by high expression of genes associated with keratins, pigmentation, and epithelium; MITF-low, characterized by low expression of genes associated with melanocytic differentiation, pigmentation, and epithelial differentiation [86]. These studies are of fundamental importance because they enable the identification of several candidate driver events in the various melanoma subtypes amenable to the development of target therapies.

The same group of investigators explored the NF1-mutated melanoma subgroup in detail [87]. Particularly, they showed that inactivating *NF1* mutations were present in 46% of melanomas expressing WT-BRAF and NRAS, occurred in older patients, were associated with a higher mutational burden, and showed a peculiar pattern of co-mutation with other RASopathy genes (particularly *RASA2*) and with *RAC1* and *ARID2*. *NF1* encodes a negative RAS regulator, and therefore it is not surprising that NF1-inactivating mutations led to increased RAS activation. NF1 mutation does not predict sensitivity to MAPK and ERK inhibitors [87]. In mouse melanoma models, NF1 mutations cooperate with BRAF mutations in driving the development of melanoma by preventing oncogene-induced senescence, thus suggesting a role for NF1 in the early stages of tumor development [88]. RNA interference studies have provided evidence that NF1 is an important mechanism of resistance to BRAF inhibitors. NF1 is strongly affected by the treatment of BRAF-sensitive melanoma cell lines with BRAF inhibitors, and NF1 knockdown abrogated the growth inhibitory effects elicited by BRAF inhibition [89]. Recently, Cirenajwis and coworkers have analyzed the clinical data present in various studies, relative to 870 melanoma patients, screened for the more recurrent molecular abnormalities (including NF1), and reached the important conclusion that NF1-mutated melanomas harbor distinct biological and clinical features [90]. In line with previous observations, they confirmed that the NF1-mutated melanoma subtype had a higher mutational burden and displayed the strongest UV mutation signature [90]. The most co-occurring mutated genes are represented by the RASopathy genes *PTN11*, *RASA2*, and *RASSF2* [90]. The majority of NF1-mutated melanomas occur in males, with older age at diagnosis. The analysis of the clinical parameters showed that NF1-mutated melanomas have a poor survival and an increased risk of death from melanoma, and these findings remained significant after adjustment for various important patient’s parameters such as age, gender, and lesion type [89]. This observation strongly supports the study of this melanoma subtype to identify suitable therapeutic targets [90].

A recent study provided the first large genome-wide comparative sequencing of cutaneous, acral, and mucosal melanomas, providing evidence of distinct mutation processes; mutational signatures of ultraviolet radiation exposure dominated cutaneous melanomas, while structural variants represented most aberrations in acral and mucosal melanomas [91]. Particularly, single-nucleotide variant and indel frequencies were markedly higher in cutaneous melanomas (49.2 mutations per megabase) than in acral and mucosal melanomas (2.6 mutations per megabase), while somatic structural variants were more frequent in acral/mucosal melanomas than in cutaneous melanomas; finally, copy number alterations were more frequent in acral/mucosal than in cutaneous melanomas [91]. The analysis of mutated genes showed remarkable differences between these three subtypes of melanomas: most acral and mucosal melanomas (51%), but only 11% of cutaneous melanomas lacked *BRAF*, *NRAS*, or *NF1* mutations [91]. Thus, cutaneous melanomas were characterized by frequent *BRAF*, *CDKN2A*, *NRAS*, and *TP53* mutations; BRAF, NRAS, and NF1 mutations were frequent in acral melanomas, and splicing factor 3B subunit 1A (SF3B1) mutations were frequent in mucosal melanomas [91]. The triple wild-type group of melanomas—comprising most acral and mucosal melanomas—was enriched of loss-of-function mutations in *CDKN2A, TP53*, and *ARID2*, and activating spot mutations in *GNAQ* and *SF3B1* [91]. *TERT* promoter mutations were the most frequent; however, neither these mutations nor ATP-dependent X-linked helicase II (*ATRX*) mutations were associated with greater telomere length [91]. Most melanomas pertaining to these three subtypes had potentially actionable mutations, mostly in components of the MAPK and PIK pathways [91].

Recently, Kong et al. analyzed a very large number of Asiatic patients with acral melanoma (514 patients) for the presence of aberrations of the Cyclin-dependent Kinase 4 (CDK4) pathway. This analysis displayed *CDK4* gain (36.5%), *CCND1* (26.7%), and *P16^IKN4a^* (CDKN2A); 32.6% of these patients contained two concurrent mutations, and 8.6% contained three aberrations [92]. The overall survival of patients with CDKN2A or CDK4 gain was significantly shorter than those without *CDKN2A* loss or *CDK4* gain; in contrast, *CCND1* gain does not modify the disease survival [92]. Experiments on melanoma cell lines and on primary melanoma cells support the sensitivity of acral melanomas to treatment with CDK inhibitors [92].

Melanomas in children and adolescents are a rare condition, but their incidence continues to rise, particularly in the age group between 15 and 19 years. Although certain predisposing factors play a role in the genesis of pediatric melanomas, most cases are sporadic. The analysis of a group of 15 conventional cutaneous pediatric melanomas showed that they have a genomic landscape comparable to that observed for adult cases, with some remarkable quantitative differences: 100% of cases displayed *TERT* promoter mutations, 87% displayed *BRAF* mutations, while no RAS mutations were observed; furthermore, these cases showed a coding mutation rate (14.4 mutations per megabase) comparable to that observed in adult melanomas and >80% of the identified single nucleotide variants (SNVs) were consistent with UV damage [93]. In contrast, in pediatric melanomas arising in congenital nevi, *NRAS*^Q61^ mutation, and *TERT* promoter mutation were observed [93].

### 3.2. Genetic Alterations of Deep Penetrating Nevi

This peculiar neoplastic lesion was described by Helwig and coworkers in 1989; this lesion was characterized by the proliferation of enlarged melanocytes, that—in contrast with common nevi—remain well pigmented in surface as well as in deep regions [94]. Some of these lesions may be misdiagnosed as melanoma. These lesions are basically benign, and only rarely undergo a malignant transformation.

A recent study provided a detailed molecular characterization of 18 of deep penetrating nevis (DPNs): these tumors were characterized by the simultaneous presence of mutations of the β-catenin pathway (6% display Adenomatous Polyposis Coli (*APC*) mutations and 89% Catenin β1 (*CTNNB1*) mutations) and of the MAPK pathway (50% display *BRAF* mutations, 33% *MAPK21*, and 6% *HRAS* mutations) [94]. Interestingly, one of these 18 DPNs do not display mutations of the β-catenin and of the MAPK pathway, but possesses *GNAQ* and *IDH1* mutations [95]. In parallel, the analysis of seven lesions displaying hybrid features of DPN and blue nevus showed the absence of mutations of the β-catenin and MAPK pathways and the presence of GNAQ mutations in 71% of cases [95]. Most β-catenin pathway activating mutations were missense mutations in exon 3 of *CTNNB1*, disrupting the phosphorylation of β-catenin and its ubiquitin degradation, determining increased β-catenin levels [94]. The majority of BRAF mutations observed in DPN are classical BRAF^V600E^ mutations; indels of MAPK21 cluster near a highly-conserved lysine within the kinase catalytic domain [95]. According to these observations, it was hypothesized that *CTNNB1* mutations confer phenotypic characteristics of DPN to classical nevi. This hypothesis is directly observed by functional studies showing that the transduction of immortalized mouse melanocytes with *BRAF^V600E^* and *CTNNB1^S33F^* resulted in the formation of large, heavily-pigmented melanocytes, highly expressing cyclin D1 and resembling DPN melanocytes [94]. Rare cases of DPN progressing to melanomas display additional mutations, such as *TP53* and *TERT* promoter mutations and copy number alterations [94].

### 3.3. Genetic Evolution of Melanoma

**Figure 2:**
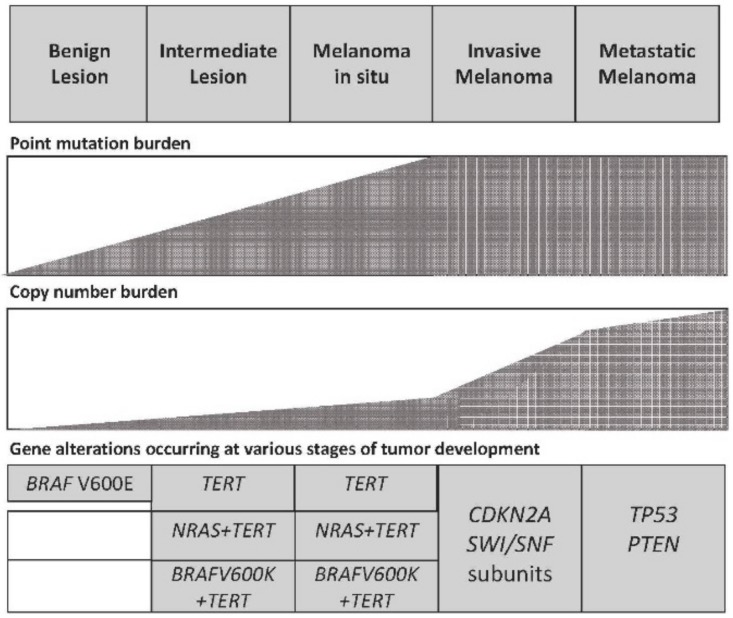
Evolution of biologic and molecular properties of melanomas during the natural progression from a benign lesion, to melanoma in situ, to invasive melanoma, and then metastatic melanoma. The mutational burden increases more at in initial stages, while copy number alterations preferentially occur at later stages of tumor development.

Large-scale sequencing studies have provided important information about the mutational profile of advanced melanomas. However, these studies do not allow determination of how the numerous genetic alterations observed in these tumors are progressively acquired during tumor development. Some recent studies have attempted to delineate the succession of events that leads to melanoma. Melanoma frequently derives from precursor lesions such melanocyte nevi, intermediate lesions, or melanoma in situ, thus offering the possibility to comparatively analyze the genetic abnormalities observed in these tumor lesions and to characterize the progressive accumulation of these abnormalities during tumor progression. Some mutations present in melanoma, such as *BRAF*, *NRAS*, *GNAQ*, or *GNA11*, and rearrangements in fusion kinases, have been observed in benign nevi, and therefore represent events acquired early during tumor progression [96]. A recent study analyzed the development of genetic alterations during melanoma progression using an approach based on the analysis of primary melanomas and their adjacent precursor lesions [97]. According to the findings observed in this important study, some conclusions were reached: (a) at the level of precursor lesions, the initial mutagenic events triggering the neoplastic transformation are represented by a number of mutations leading to activation of the MAPK pathway (such as *BRAF^V600E^*); (b) at an intermediate stage of tumor progression, *NRAS* and additional driver mutations are observed: interestingly, in these tumor lesions *TERT* promoter mutations are very frequent (77% of cases), suggesting that the selection of mechanisms precluding cell senescence are an early event during melanoma progression; (c) biallelic inactivation of *CDKN2A* and *PTEN* and *TP53* mutations were found only in advanced melanoma lesions (Figure 2) [97]. Furthermore, the mutational burden clearly increased from benign to intermediate and then to invasive melanoma lesions, with strong evidence of the effects of ultraviolet signature evident at all stages of tumor evolution [97].

A recent study better clarified the role of TERT promoter mutations during the evolution of genetic abnormalities leading to melanoma development [97]. In benign nevi, *BRAF* mutations occur and lead to a stimulation of melanocyte proliferation; melanocyte proliferation results in progressive shortening of telomeres, since these cells do not possess a telomere-maintaining mechanism and undergo a process of growth arrest known as oncogene-induced senescence [98]. If additional genetic alterations occur (e.g., *CDKN2A* inactivation), melanocytes receive an additional proliferative stimulation and divide, and when telomeres become very short, these cells undergo a process of replicative senescence. For the neoplastic progression of these cells, the activation of a telomere-maintaining mechanism is required, which is dictated by the acquisition of telomerase promoter mutations (TPMs) [98]. In these transformed melanocytes (still premalignant), TPMs are unable at this stage to induce a sufficiently high telomerase expression and their effect is limited and transient, sufficient to bypass replicative senescence [98]. However, this proliferative stimulation determines a further shortening of telomere length and exposes the cells to a new replicative block. At this point, the transformed cells either remain in this condition and undergo apoptosis or, alternatively, acquire new genetic alterations and again activate a telomere-maintaining mechanism which is required for cell proliferation and promotes telomeres-driven instability and become immortalized. Therefore, this study supports a two-step “escape from crisis model”, where two sequential activations of telomerase are required first to bypass oncogene/replicative-induced senescence and then to bypass proliferation crisis related to telomere shortening [98].

### 3.4. Genetic Abnormalities of Spitz Melanomas

Spitzoid tumors are melanocytic tumors with peculiar histological features, predominantly occurring during the first two decades of life. Tumors with spitzoid morphology encompass a group of neoplasias with a wide spectrum of tumor properties, ranging from a group of benign neoplasias defined as Spitz nevus, to a group of tumors with low-grade, borderline malignant potential, defined as atypical Spitz tumors (ASTs) and fully malignant tumors, comparable to standard melanomas and called spitzoid melanomas. These tumors are composed of large epithelioid- and/or spindle-shaped melanocytes that contain large nuclei and prominent nucleoli. Importantly, ASTs are tumors with a limited malignancy, able to spread only at the level of loco-regional lymph nodes, while spitzoid melanomas generate extranodal metastases. Spitz nevi have an intrinsic tendency to evolution. A recent study based on the follow-up of 27 Spitz nevi provided evidence that only seven remained stable over time, while 20 exhibited an evolution; therefore, the most common biologic behavior for Spitz nevi is evolution [99]. Few studies have characterized the mutational spectrum of spitzoid tumors. In this context, initial studies have shown that spitzoid neoplasms lack some of the mutations typically observed in melanomas, such as *NRAS*, *Kit*, *GNAQ* or *GNA11*. A subset of spitzoid tumors were shown to display *HRAS* mutations or *BRAF* mutations, associated with BRCA1-associated protein 1 (BAP1) biallelic loss. Only recent studies have more systematically characterized the mutation spectrum of spitzoid tumors. Thus, a study carried out on 140 spitzoid tumors reported the frequent occurrence of kinase fusions in spitzoid neoplasms, involving *ROS1,* Anaplastic Lymphoma Kinase (*ALK*), *BRAF*, *RET*, and Neurotrophic Receptor Tyrosine Kinase (*NTRK1*) [100]; the *ROS1*, *ALK*, and *BRAF* fusions are more frequent in Spitz nevus and AST than in Spitz melanoma; in contrast, *NTRK1* fusions are more frequent in Spitz melanoma than in Spitz nevis and AST [100]. Importantly, kinase fusions were observed in about 55% of Spitz nevus, 56% of AST, and 39% of Spitz melanoma [100]. All fusions occurred in a mutually exclusive pattern, and no fusions were detected in tumors with *HRAS* mutations [100]. The presence of kinase fusions during the entire biologic spectrum of spitzoid tumors strongly suggests that the fusions are an early event in the pathogenetic development of these tumors and are necessary—but not sufficient alone—for malignant transformation [100]. Recently, *NTRK3* kinase fusions were reported in some Spitz tumors: NTRK3 fusion proteins constitutively activate MAPK, and their activities can be inhibited by DS-6051a, a small molecule inhibitor specific for *NTRK* and *ROS1* [101]. A recent study explored the existence of a possible link between the type of fusion kinase and the biologic/molecular properties of spitzoid tumors. *BRAF* fusion cases were characterized by a predominance of epithelioid cells and exhibit a sheet-like growth pattern or a dysplastic architecture; importantly, these cases have high-grade nuclear atypia, are frequently diagnosed as spitzoid melanomas, and develop copy number gains in the kinase domain of the fusion protein [102].

Kinase fusions are a frequent event in spitzoid tumors, but are rare events in conventional melanomas. In addition to the abovementioned differences, a comparison of the mutational spectrum of spitzoid melanoma and conventional melanoma also showed many similarities between these two tumors: the mutational burden of spitzoid melanomas (747 ± 138 mutations) and conventional melanomas (758 ± 97 mutations) is highly comparable; there is a large overlap of driver mutations in spitzoid and conventional melanomas, with 66% of spitzoid melanomas displaying mutations affecting the MAPK pathway (37% *BRAF* mutations, 18% *RAS* mutations, and 11% *NF1* mutations) [103]. Furthermore, like conventional melanomas, spitzoid melanomas displayed inactivating mutations of *CDKN2A*, *TP53*, *RAC1*, *PTEN*, *IDH1*, and *ARID2* [103].

Recently, Yeh and coworkers reported *MET* fusion kinases in 0.5% of melanocytic tumors; interestingly, all these cases displayed spitzoid histopathologic tumors [104]. *MET* fusions occurred in tumors that ranged from benign to malignant, and this finding strongly suggests that *MET* fusion kinases represent an early event during melanoma progression [104]. *MET* fusion kinases occur in a mutually exclusive pattern with activating mutations of known melanoma oncogenes, and represent a potential therapeutic target in these patients [104].

These observations also indicate that the presence of kinase fusions cannot be used as a marker of malignant transformation of spitzoid tumors. However, a recent study by Lee and coworkers showed that the presence of *TERT* promoter mutations is predictive of aggressive clinical behavior in patients with spitzoid melanocytic neoplasms [104]. In fact, these authors showed the presence of *TERT* promoter mutations among 56 patients with spitzoid tumors—only in those displaying distant metastases, but in none of the remaining 52 patients with a benign disease [105]. It is important to note that the heterogeneity of the fusion transcripts observed in spitzoid tumors correlates with the morphologic and clinical diversity of patients with spitzoid tumors [106]. Among these patients, those with *TERT* promoter mutations have a metastatic disease, with fatal outcome [106].

Unlike in spitzoid melanoma, kinase fusions are rare in classical melanomas. In fact, recent studies have explored the occurrence of *ALK* fusions in primary and metastatic cutaneous melanomas, but none out of 600 melanomas—including those positive for ALK expression—displayed the ALK translocation [107]. Interestingly, ALK^+^ melanomas (observed in about 2.5% of cases) express a peculiar ALK isoform which lacks the extracellular and transmembrane domain of wild-type ALK, consisting of the intracellular tyrosine kinase domains and originating from an alternative transcriptional initiation site of the *ALK* gene [108].

The identification and classification of Spitz tumors remains a great unsolved problem. However, the recent molecular studies allowed the proposal of a rational molecular classification for these tumors: (i) spitzoid tumors with *HRAS* alterations or 11p amplification; (ii) homozygous deletion of 9p21; (iii) isolated loss of 6q 23; (iv) *BAP1* loss and *BRAF^V600E^* mutation; (v) translocations involving various oncogenic kinases, such as *ROS1*, *ALK*, *NTRK1*, *NTRK3*, *BRAF*, *RET*, and *MET*; (vi) mutations at the level of the *TERT* promoter [109,110]. A high proportion of spitzoid tumors are characterized at the molecular level by kinase fusion proteins (55% of Spitz nevi, 56% of atypical spitzoid tumors, and 39% of Spitz melanoma) [110]. The screening of spitzoid tumors for kinase fusions allowed data to be obtained on these tumors, classified on the basis of the various kinase fusions. *ALK* fusions-positive spitzoid tumors (10–17% of these tumors) usually display a single tumor lesion, preferentially located on the extremities, are usually polyploid, and are composed of spindled (fusiform) rather than epithelioid melanocytes, with a plexiform growth pattern [111]. *ROS1* fusions were found in 17% of spitzoid tumors; these tumors do not have a peculiar architectural structure and are composed of dermal nests of epithelioid and spindled melanocytes [111]. *NTRK1* encodes the receptor *TRKA*, and the tumors with *NTRK1* fusions show classical spitzoid features and are composed of epithelioid melanocytes [111]. As mentioned above [110], spitzoid tumors with *BRAF* mutations display some peculiar properties. Interestingly, BRAF fusions were observed in about 3% of melanomas, including some non-spitzoid melanomas; in these tumors, BRAF fusions are mutually exclusive of other mutations in the MAPK pathway and determine a constitutive BRAF kinase activity; the BRAF fusions are not sensitive to the inhibitory effects of vemurafenib, but are sensitive to MEK inhibitor [111].

According to the acquisition of these data on molecular genetics, spitzoid lesions can now be reasonably classified according to their distinctive molecular-genetic alterations as spitzoid lesions with (1) 11p amplification and/or *HRAS* mutations; (2) isolated loss of 6q23 by fluorescence in situ hybridization (FISH); (3) homozygous deletion of 9p21 by FISH; (4) *BAP1* loss and *BRAF^V600E^* mutation; (5) translocations involving any of a number of different oncogenic kinase drivers, including *ROS1, ALK, NTRK1, NTRK3, MET, BRAF*, and *RET*; (6) *TERT* promoter mutations [109,112].

### 3.5. Genetic Factors Controlling Melanoma Development

Approximately 5–10% of melanoma cases are familial; most familial cases lack germ-line mutations in known cancer susceptibility genes [112]. Furthermore, most familial melanomas lack germ-line mutations in genes that are commonly mutated in sporadic melanoma [113]. Thus, about 10% of all cases of cutaneous melanoma occur in patients with a personal or family history of the disease. These analyses included melanoma-prone families and individuals with multiple cutaneous melanomas. A recent study reported the analysis of 27 melanoma-prone families, showing: *CDKN2A^V126D^* mutation in 7/27; *CDKN2A^A148T^* was observed in 7/27 (this germline mutation was observed in 7/146 normal healthy blood donors); MC1R melanoma-associated polymorphism was detected in 78% of cases (and 66% in healthy donors); the *MITF^E318K^* mutant was observed in 7% of cases (and in 0.7% of healthy controls) [114]. 

Seven population-based genome-wide association studies (GWAS) of cutaneous malignant melanoma have been reported, allowing the identification of 13 regions that reach genome-wide significance. As expected for common variants influencing cutaneous malignant melanoma risk, many of these loci contain genes involved in one of two heritable risk phenotypes for melanoma, such as pigmentation (*MC1R,* Tyrosinase (*TYR*), Solute Carrier Family 45 member 2 (*SLC45A2*), and Agouti Signaling Protein (*ASIP*)) and nevus counts (*CDKN2A, TERT*, and Phospholipase A2 Group 6 (*PLA2G6*)) [115]. DNA repair genes such as Poly ADR Ribose Polymerase 1 (*PARP1*) and Ataxia-Telangiectasia-Mutated (*ATM*) are present at two other loci [115]. The 1q42.1 melanoma risk allele is correlated with higher *PARP1* level [116]. In human primary melanocytes, PARP1 promotes cell proliferation and rescues *BRAF^V600E^*-induced senescence phenotypes in a PARylation-independent manner; furthermore, *PARP1*-transformed *TERT*-immortalizes melanocytes expressing *BRAF^V600E^* [116]. PARP-mediated senescence rescue was accompanied by transcriptional activation of the MITF transcription factor [116].

One of the major genetic determinants of cutaneous melanoma risk in the general population is represented by disruptive variants (R alleles) in the melanocotin 1 receptor gene (*MC1R*). MC1R is a G protein-coupled receptor expressed on the membrane of melanocytes and signals to downstream molecular effectors, including the microphtalmia-associated transcription factor: through these targets, this gene controls skin pigmentation and melanocyte proliferation and apoptosis. Activation of MC1R in melanocytes by α-melanocyte-stimulating hormone (α-MSH) stimulates cAMP signaling and melanin production and enhances DNA repair after ultraviolet irradiation. As stated above, melanin is generated by melanocytes in two major forms: eumelanin and pheomelanin. While melanin is brown or dark in color and exerts a protective effect against the damage derived from UV-exposure, pheomelanin is red and is associated with type I/II skin, freckles, red hair, and an inability to tan, and confers a high photosensitivity and a tendency to sunburn after exposure to UV light. Population sequencing studies have shown the presence of null or hypomorphic MC1R alleles (defined as R alleles), strongly associated with the red hair skin and the light skin phenotype [117,118]. Other missense variants are defined as r alleles and are less strongly associated with the red hair color [116]. The large majority of red-heads are R/R, and they only rarely have 0 or 1 R allele; the level of tanning after UV exposure depends on the number of R alleles [119]. The physiologic role of MC1R is not restricted only to the regulation of skin pigmentation, but also extends to the phosphorylation of DNA repair proteins and to the activation of survival pathways [120]. Polymorphism in MC1R is linked to increased melanocyte apoptosis and inefficient DNA repair, and is related to increased melanoma risk [121].

The idea that loss of function of *MC1R* contributes to oncogenic transformation of melanocytes is supported by animal models based on the finding of a cooperation between null *MC1R* and BRAF^V600E^ to promote melanoma development [1]. These findings suggest that loss of MC1R function may be oncogenic, even in the absence of UV light. A recent study provided evidence that MC1R contributes to the mutational load in melanoma. Mutations observed in melanoma are predominantly of the C > T transition type, due to the production of cyclobutane pyrimidine dimers (CPDs) in response to solar UV damage; however, other mutational classes (e.g., C > A transversions) have also been observed, such as those occurring in key driver genes such as *BRAF* and *Kit* [121]. The comparative analysis of melanoma patients with WT and null *MC1R* alleles have a significantly higher mutational load than individuals with no R alleles [121]. Interestingly, in individuals carrying R alleles, a significant and similar enrichment not only of C > T mutations, but also of non-C T mutations was found, thus supporting the existence of multiple mutagenic processes in melanoma development [122]. A recent study showed that protein palmitoylation could represent a strategy to rescue the activity of variant *MC1R*, associated with melanoma development [123]. In fact, biochemical studies have shown that MC1R palmitoylation—mediated by the acyl transferase Zinc Finger DHHC-Type Containing 13 (*ZDHHC13*)—is of fundamental importance for the activation of MC1R signaling and consequent stimulation of pigmentation, UVB-induced cell-cycle arrest and control of senescence, and of melanomagenesis in vivo [123]. Importantly, pharmacological activation of palmitoylation rescues the functional defect of MC1R variant and prevents melanomagenesis [123].

Germline mutations of *CDKN2A* have been observed in about 20% of familial melanoma kindred from various countries. A relationship between *CDKN2A* mutation carriage and atypical nevus counts was observed, but not with typical nevi (i.e., 2 mm and 5 mm nevi) [124]. Other recent studies have explored the relationship between germline *CDKN2A* mutations and multiple primary melanomas (MPMs), showing that familial MPMs with germline *CDKN2A* mutations display several peculiarities, such as early age of appearance and worse survival, compared to cases with familial and sporadic wild-type MPM [125]. 

As mentioned above, MITF encodes an oncogenic-lineage specific transcription factor that plays a key role in melanocyte differentiation. A germline mutation, *MITF^E318K^*, was identified in human patients and predisposes to nevus and melanoma formation. The molecular mechanism underlying the oncogenic activity of MITF^E318K^ was recently clarified, showing that the mutant *MITF* was less sumoylated than WT MITF, and its genetic introduction in mice determined a slightly hypopigmented phenotype [126]. In an animal model of melanoma, *MITF^E318K^* was not sufficient to cooperate with *BRAF^V600E^* alone to generate melanoma, but it was capable of accelerating tumor formation on a *BRAF^V600E^* and PTEN-deficient background [126]. Experiments on human melanocytes showed that the pro-tumorigenic activity of *MITF^E318K^* was related to its capacity to inhibit oncogene (BRAF^V600E^)-induced senescence [126].

### 3.6. Genetic Abnormalities of Uveal Melanomas

Recent studies have explored the genetic abnormalities observed in uveal melanomas, providing evidence that these tumors have a pattern of genetic abnormalities markedly different compared to those observed in cutaneous melanomas. Uveal melanoma is a malignant tumor originating from melanocytes of the choroid plexus, ciliary body, and iris of the eye, and represents the most common intraocular tumor in adults. It represents 3.1% of all melanomas, has an aggressive clinical course, is frequently metastatic, and is associated with poor survival (only 10–15% of patients survive at 1 year). In contrast to cutaneous melanomas, uveal melanomas lack mutations in *BRAF*, *NRAS*, and *Kit* genes, possess typical chromosomic abnormalities, and have a very pronounced tendency to metastasize at the level of the liver. Recent studies have shown the occurrence of some frequent and typical mutations in uveal melanoma. Thus, frequent somatic mutations in the heterotrimeric G protein α-subunit, GNAQ, have been reported in 83% of blue naevi (intradermal melanocytic proliferations forming discrete bluish moles) and 46% of ocular melanomas of the uvea [127]. The *GNAQ* mutation occurs at the level of codon 209 in the Ras-like domain and determines the constitutive activation of this protein and, consequently, of the MAPK pathway (Table 3) [127]. Interestingly, a *GNAQ* mutation was observed in 6% of patients with naevus of Ota, a condition predisposing to the development of uveal melanoma in which a proliferation of intradermal melanocytes gives rise to hyperpigmentation of the conjunctiva and periorbital skin. Mutant *GNAQ* drives uveal melanoma development, activating various downstream signaling pathways, including Rho/Rac, YAP, and PLC/PKC. In this complex signaling pathway, a key role is played by the GTPase ADP-Ribosylation Factor 6 (ARF6) which acts as an initial target of GNAQ, coordinating the activation of all these signaling pathways and also inducing the activation of β-catenin signaling [127]. ARF6 acts as a coordinator of all these pathways by a common mechanism involving the trafficking of both GNAQ and β-catenin from the cell membrane to cytoplasmic vesicles and the nucleus [128]. In line with these observations, the pharmacological blocking of ARF6 function with a small-molecule inhibitor induced an inhibitory effect on the proliferation of uveal melanoma cells and of tumorigenesis in a model of uveal melanoma, thus supporting the functional relevance of this pathway and suggesting its possible targeting at a therapeutic level [128].

Another study carried out by the same investigators showed frequent mutations in uveal melanoma of the gene *GNA11*, encoding for a *GNAQ* paralogue. Mutations affecting the residue Q209 (glutamine is mutated either to proline or leucine) were present in 7% of blue naevi, 32% of primary uveal melanomas, and 57% of uveal melanoma metastases (Table 3). The incidence of Q209 mutations in *GNAQ* was 55% in blue naevi, 45% in uveal melanomas, and 22% in uveal melanoma metastases [129]. In addition to Q209 mutations, more rarely somatic mutations in exon 4 (affecting R183) have been observed both in *GNA11* and *GNAQ* genes: these mutations are mutually exclusive from those occurring at Q209 [129]. These mutations in *GNA11* determine a constitutive activation of the MAPK pathway and induce the formation of spontaneously metastasizing tumors in suitable mouse models [129]. The conclusion of these studies was that more than 80% of uveal melanomas had somatic mutations in *GNAQ* or *GNA11*, implying that MAPK activation is a major contributor to the development of uveal melanoma.

**Figure 3:**
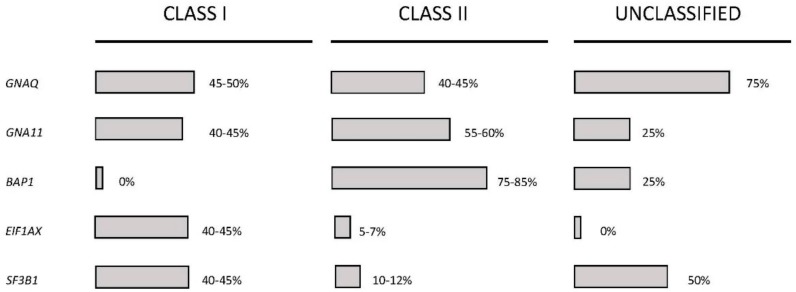
Driver mutations occurring in uveal melanomas, subdivided into class I, class II, and unclassified subtypes.

A molecular classification of uveal melanoma was currently reported and adopted for the identification of this tumor into two different risk classes: class I associated with a low risk of metastasis, and class II associated with high risk of metastasis. Among the various prognostic criteria, the monosomy of chromosome 3 strongly associates with class II tumors. According to these findings, Harbour et al. identified frequent mutations of the gene encoding *BAP1* located on chromosome 3p21.1, occurring in 86% of class II uveal melanomas, but only in <5% of class I uveal melanomas (Figure 3) [130]. Most BAP1 gene mutations corresponded to premature stop codons; therefore, it is not surprising that BAP1 protein levels were markedly lower in class II tumors compared to those observed in class I tumors [130]. It is of interest to note that a minority of uveal melanoma patients exhibited germline *BAP1* mutations, suggesting a possible role of mutations of this gene as a genetic factor predisposing to cancer development. In line with this hypothesis, a novel autosomal dominant syndrome was recently reported in two families that is caused by germline mutations of BAP1; it is characterized by a high penetrance of melanocytic tumors with peculiar clinical and histopathological feature, associated with an increased risk of uveal melanoma [131]. These observations were also confirmed by the analysis of families with the predisposition to develop uveal melanoma: in these families, a germline truncating mutation in the BAP1 gene was observed; in these cases, the development of uveal melanoma was associated with the concomitant development of another tumor—either meningioma, lung cancer, or neuroendocrine carcinoma [132]. According to all of these findings, a mutational model of uveal carcinoma was developed. This model implies that the initiating event is represented by an activating mutation occurring in a uveal melanocyte cell and acting via induction of the cell cycling triggered by constitutive MAPK activation. In most cases, the mutant clone does not progress and is either eliminated by apoptosis or gives rise to a benign nevus. More rarely, the initial clone progresses due to the accumulation of additional genetic abnormalities either along a low-risk or a high-risk melanoma, depending on the type of mutations accumulated [133].

**Figure 4:**
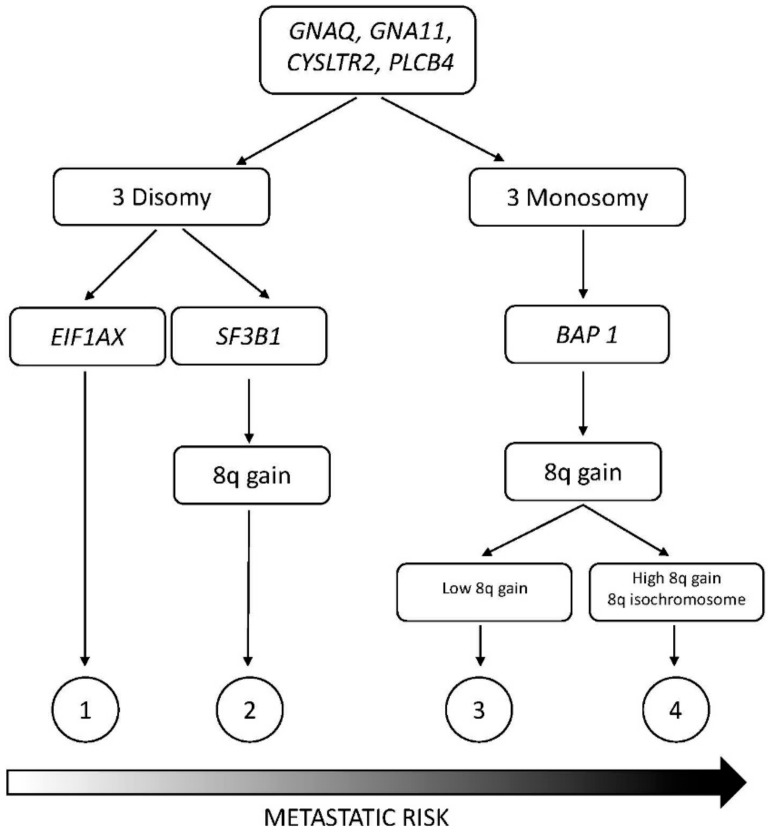
Mutational evolution of different molecular subtypes of uveal melanoma. An initial event, common to all tumor groups, is represented by GNAQ or GNA11 or CYSLTR2 or PLCB4 mutations determining constitutive MAPK activation. After this initial event, a first major branching determines the formation of two branches: one characterized by chromosome 3 disomy, and the other by chromosome 3 monosomy and BAP1 mutations. The subsequent sub-branching of the 3 disomy branch into sub-branches 1 and 2 is dictated by the acquisition of EIF1AX or SF3B1 mutations, respectively. The sub-branching of the 3 monosomy branch into sub-branches 3 and 4 is dependent upon the level of acquisition of a chromosome 8q gain, low in sub-branch 3 and high in sub-branch 4.

This model of uveal melanoma development was supported by a recent study of whole-genome sequencing of uveal melanomas [134]. Unsupervised hierarchical clustering of uveal melanoma samples based on the analysis of copy number alterations showed the existence of four subgroups, from A to D [134]. Groups A and B involved tumors displaying chromosome 3 monosomy, chromosome 8q gain, and in some cases, chromosome 8p loss; group B tumors also had loss of chromosome 6q; groups C and D displayed less chromosome abnormalities, with group C tumors not showing major aneuploidies and group D displaying gains of the distal segment of chromosome 8q [134]. Tumors displaying chromosome 3 monosomy were associated with BAP1 mutations (77% of cases), while *SF3B1* and *EIF1AX* mutations were observed among tumors C and D [133]. Considering the hallmark driver mutations in the *GNAQ* and *GNA11* paralogs were observed in 100% of cases, a model of tumor evolution based on genetic mutational events progressively occurring in all the four tumor subgroups is provided (Figure 4). An initial event, common to all tumor groups, is represented by *GNAQ* and *GNA11* mutations, determining constitutive MAPK activation. After this initial event, a first major branching determines the formation of two branches: a branch A/B, characterized by functional loss of BAP1 and copy number loss of chromosome 3 and copy number gains of chromosome 8q; a branch C/D, characterized by relatively normal chromosomal ploidy and acquisition of *EIF1AX* or *SF3B1* mutations. Subsequent sub-branching of A/B in A and B is dictated by the occurrence or not of chromosome 6q deletion; the sub-branching of C/D in C and D is dependent upon the occurrence or not of chromosome 8q gain [133]. 

Risk of metastatic disease and survival is strongly associated with the molecular features of the tumor. Particularly, the status of chromosome 3 allows two prognostically different uveal melanoma groups to be distinguished: (a) metastatic disease strongly associated with chromosome 3 monosomy; (b) non-metastatic disease associated with disomy of chromosome 3 [131]. Recent studies showed the presence of recurrent mutations of the *EIF1AX* and *SF3B1* genes preferentially associated with chromosome 3 disomy; in fact, in a group of uveal melanoma patients with chromosome 3 disomy, the frequency of EIF1AX (48%) and *SF3B1* (29%) was clearly higher than in patients with chromosome 3 monosomy, where *EIF1AX* and *SFB31* mutations together exhibited a frequency of 5.7% (Table 3) [135]. In contrast, patients with partial chromosome 3 monosomy—who usually have good prognosis—have uveal melanomas more similar to those with chromosome 3 disomy. In fact, 8% of these patients displayed *EIF1AX* mutations and 54% *SF3B1* mutations [136]. Sequencing of *SF3B1* and *EIF1AX* in ten uveal melanomas with disomy 3 who developed metastases showed that none of them displayed *EIF1AX* mutations, while 30% harbored a mutation in *SF3B1* [136]. At the structural level, all *EIF1AX* mutations caused in-frame changes affecting the N-terminus of the protein, whereas the large majority of SF3B1 mutations affected an alteration of Arg 625 [136]. The findings concerning the *SF3B1* gene were independently confirmed in another study showing that mutations of this gene are observed in low-grade uveal melanomas associated with good prognosis [136].

*SF3B1* encodes for a core component of the U2 small nuclear ribonucleoprotein complex of the spliceosome, and is involved in early stages of splicing. *SF3B1* hot spot mutations in uveal melanoma are associated with deregulation of a subset of splice junctions, caused by the use of alternative 3’ss (AG’) upstream of the canonical 3’ss (AG) [137]. Importantly, the *SF3B1*-mutant pattern was not reproduced by either gene knockout or overexpression, thus suggesting that the mutant displays a change of function [137]. It is of interest to note that the impact of *EIF1AX* and *S3BF1* mutations on the prognosis of uveal melanomas is different, in that patients with tumors harboring *EIF1AX* mutations rarely demonstrated metastases and had a longer disease-free survival; on the other hand, patients with chromosome 3 disomy and with SF3B1 mutation had an enhanced metastatic risk compared with those without an *SF3B1* mutation [138]. Finally, *SF3B1* and *EIF1AX* mutations have been reported in primary leptomeningeal melanocytic neoplasms, thus suggesting a similarity between these tumors and uveal melanomas [139].

A recent study reported recurrent mutations of G-protein-coupled receptor *CYSLTR2* (cysteinyl leokotriene receptor 2) exclusively occurring in uveal melanoma patients not displaying *GNAQ, GNA11*, and phospholipase C, beta 4 (PLCB4) mutations (in about 40% of these patients) [140]. *CYSLTR2* mutations occur in 3% of uveal melanoma patients, and 50% of cases associate with *BAP1* mutations (Table 3). The mutant CYSLTR2 constitutively activates endogenous Gα_q_, and its enforced expression in melanocytes promotes tumorigenesis [140].

Another recent study reported the recurrent mutation in *PLCB4*, occurring in about 4% of uveal melanomas (Table 3) [141]; importantly, *PLCB4^D630Y^* mutations are mutually exclusive with mutations in *GNA11* and *GNAQ*, in line with the observation that *PLCB4* is a canonical downstream target of *GNA* genes [141]. Therefore, PLCB4 hot-spot mutations are gain-of-function mutations leading to the activation of the same MAPK signaling pathway induced by *GNA11* and *GNAQ* mutants [141].

The identification of several driver mutations in uveal melanoma allows the unique opportunity to define molecular subtypes. Thus, GNAQ, GNA11, PLCB4, and CYSLTR2 form four modules of independent mutated uveal melanomas. The mutations of these four genes are related to different steps of G membrane protein activation: *CYSLTR2* activation of *GNAQ* or *GNA11* promotes the exchange of GDP for GTP and binding of GNAQ/GNA11 to PLCB4 to activate cleavage of phosphatidylinositol 4,5-biphosphate (PIP2) to produce diacylglycerol (DAG) and inositol triphosphate (IP_3_), resulting in calcium release [142]. On the other hand, the analysis of driver mutations, combined with analysis of the major prognostic marker (chromosome 3 status) allows a tentative definition of molecular prognostic biomarkers. In this context, a recent study by Decatur and coworkers performed an analysis of the association of the main driver mutations with patient outcome [143]. This analysis showed that: (a) *GNAQ* and *GNA11* mutations are mutually exclusive, *BAP1* and *EIF1AX*, as well as *BAP1*, *SF3B1*, and *EIF1AX* are almost mutually exclusive; BAP1 mutations are associated with a poor prognostic risk and high metastatic risk; EIF1AX and SF3B1 are usually biomarkers of good prognosis [143]. A recent study showed that the expression of the oncogene PRAME in a group of uveal melanoma patients allowed the identification of a group of class 1 patients with intermediate metastatic risk, and these uveal melanomas often harbor SF3B1 mutations [144]. According to these findings, it was proposed that *SF3B1* mutations help to define a subgroup of uveal melanomas associated with metastatic risk that is intermediate between uveal melanomas with *BAP1* mutations (high risk) and uveal melanomas with *EIF1AX* mutations (low risk).

The dramatic progresses made in the understanding of the molecular abnormalities underlying uveal melanoma allowed an integrative analysis to be performed based on multiplatform analyses, proposing a molecular and clinical classification of these tumors in four groups: two associated with poor prognosis monosomy 3 (M3) and two with better prognosis disomy 3 (D3) [145]. *BAP1* loss (86% of cases) follows M3 occurrence, and is associated with a global DNA methylation state that is clearly distinct from that observed in D3 tumors [145]. Despite showing a comparable global DNA methylation profile, M3 tumors were divided into two subgroups with different biological profiles and clinical outcomes based on the transcriptional and mutational profiles [145]. Group 4, with poorest prognosis, was characterized by the occurrence in 100% of cases of chromosome 8q gain, while in group 3 (the other group of M3 tumors) chromosome 8q gain occurred in 25% of patients [145]. It was suggested that loss of *BAP1* function may result in defective DNA damage repair/response, thus playing an active role in isochromosome 8q formation. The D3 group is subdivided into two subgroups 1 and 2: group 1, characterized by *EIF1AX* mutations and by the best prognosis (low metastatic potential); group 2, characterized by Splicing Factor 3b Subunit 1/Serine-Arginine Rich Splicing Factor 2 (*SF3B1/SRSF2*) mutations and by a less-optimal prognosis [146]. This classification is also supported by RNA (mRNA, microRNA (miRNA), long non-coding RNA (lncRNA)) and cellular pathway activity profiles [144]. It is of interest to note that the presence of a tumoral immune infiltrate characterizes M3 tumors, and is therefore associated with a poor prognosis [145].

It is important to make a brief comparison of the uveal melanomas with cutaneous melanomas. Although both of these tumors derive from the malignant transformation of melanocytes (uveal melanocytes in uveal melanoma and cutaneous melanocytes in cutaneous melanoma), they differ in etiopathogenesis (cutaneous melanoma is largely related to UV radiation, while uveal melanoma is not linked to UV radiation), in the landscape of genetic alterations, and in their different metastatic routes [146]. Finally, the two tumors also differ in their response to treatment. In fact, in cutaneous melanomas consistent improvements in disease outcome have been achieved in recent years using BRAF/MEK inhibitors and various immunotherapeutic agents, yet these approaches have failed in uveal melanomas [146]. However, molecular studies have identified several actionable molecular abnormalities in uveal melanoma, suitable for the development of new therapeutic strategies [146].

### 3.7. Genetic Abnormalities of Mucosal Melanoma

Malignant melanoma of the mucosal membranes is a rare neoplasm, representing 1–1.5% of all melanomas in Caucasians. These tumors develop from melanocytes present in mucosal membranes, and their biological function in these tissutal areas is largely unknown; these melanomas are more frequently localized at the head/neck region, anorectal region, and female genital tract. Although rare in Caucasians, mucosal melanoma is the second most common subtype in Asians; its incidence is similar in White and Black people. The mutational pattern of mucosal melanoma is different from that observed in cutaneous melanomas: *BRAF* mutations are rare in mucosal melanomas (5–10%) [147], except for conjunctival melanomas (about 30%) [148]; *NRAS* mutations were observed in about 15–20% in mucosal melanomas [146]; *NF1* mutations were frequently identified in mucosal melanomas (about 20% of cases) [147]; *TERT* promoter mutations were more rare in mucosal melanomas (about 5%) than in conventional cutaneous melanomas [147]. Most of the *NF1* mutations observed in mucosal melanomas are inactivating mutations [147]. The lower frequency of *TERT* promoter mutations observed in mucosal melanomas compared to cutaneous melanomas may be due to the very limited UV-exposure of these tumors [147]. Other recent studies have reported the frequent occurrence (37%) of the *SF3B1* gene of the spliceosome pathway in mucosal melanomas [149]; in cutaneous melanomas, this gene is mutated in only 4% of cases [149]. The frequent commutation of *NF1* and *Kit* in mucosal melanomas was also observed [149].

These studies indicate that *NF1* and *RAS* mutations are the two driver mutational events more frequent in mucosal melanoma. *NF*1 and *RAS* mutations are mutually exclusive, while *KRAS* mutations occur alone or in association with *NF1* mutations [147]. *BRAF* in mucosal melanoma is mutated in <10% of patients, and these mutations occur in association or not with *NF1* mutations [147]. *Kit* mutations occur in 7–10% of mucosal melanoma, and are observed not in association with *NF1, RAS,* or *BRAF* mutations; interestingly, *Kit* mutations are particularly frequent in vulvar melanomas [147]. Importantly, more than 40% of patients with mucosal melanoma display mutations in the MAPK pathway [147]. These findings indicate that the principal mechanisms driving mucosal melanomas are not attributable to ultraviolet radiation, and imply alternative carcinogenic mechanisms. In this context, it is of interest to note that some mucosal melanomas exhibited mutated genes such as *GNAQ* and *SF3B1*, previously observed in uveal melanoma. Finally, it is important to note that mucosal melanomas are only rarely driven by TP53, PTEN, or RB1 pathway alterations.

Conjunctival melanoma may be considered as a subtype of mucosal melanoma. Conjunctival melanoma accounts for about 5–10% of all ocular melanomas, and displays a genomic landscape in terms of recurrently mutated genes (with *BRAF* and *NRAS* as the mostly frequently mutated gene) and copy number alterations similar to those observed in cutaneous melanomas, but entirely distinct from uveal melanomas (the other ocular melanomas) [150]. An important element of distinction between conjunctival melanoma and uveal melanoma is given by the analysis of *TERT* promoter mutations: these mutations were present in about 30% of the former ones, but were absent in the latter ones [150]. 

Recent studies suggest that among mucosal melanomas, those originating at the level of vulva and vagina display unique properties. Primary melanoma of the female genital tract accounts for about 1% of all melanomas. Most of these melanomas originate from the vulva (75%), and less originate from the vagina (25%). *BRAF*, *Kit*, and *APC* were the genes most frequently mutated in gynecological melanomas [151]. In a recent study based on the sequencing data on >50 gynecological melanomas, these tumors displayed a *BRAF* mutation rate of 26%, compared to 8.3% observed in mucosal melanomas and 36% in cutaneous melanomas [151]. Another molecular property of gynecological melanomas is that they display a higher *Kit* mutation rate (22%), compared to about 9% of other mucosal melanomas [151]. At the protein level, *Kit* was expressed in about 75% of gynecological melanomas [151]. *NRAS* mutations were rare in gynecological melanomas [151]. These observations have implications for the therapy of gynecological melanomas, whose prognosis is poor [151].

### 3.8. Genetic Abnormalities of Desmoplastic Melanoma

**Figure 5:**
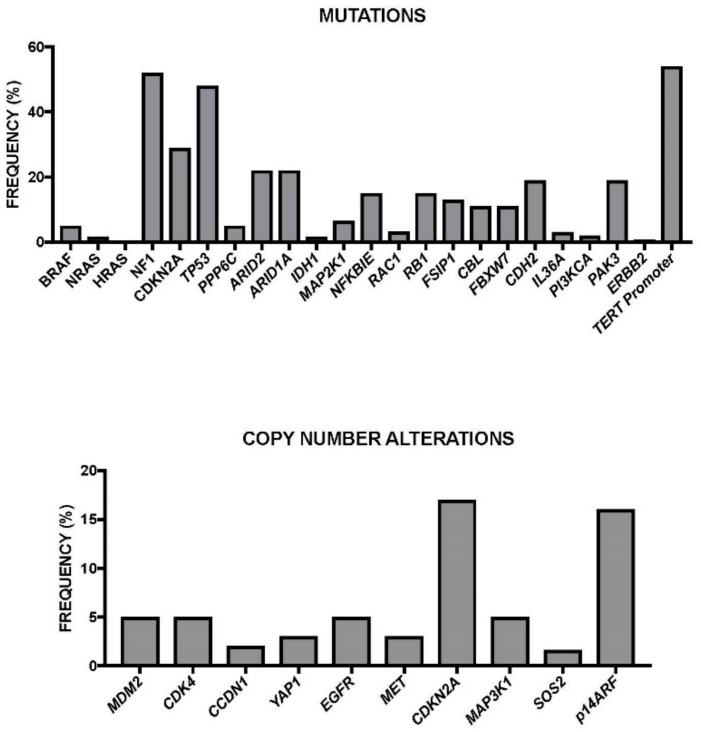
Recurrent gene mutations and copy number alterations occurring in desmoplastic melanoma.

Desmoplastic melanomas comprise about 4% of all primary melanomas. Desmoplastic melanoma is a variant of cutaneous melanoma with sarcomatous histology, peculiar biological and clinical features, and unknown pathogenesis. This rare melanoma subtype typically occurs at the level of chronically sun-exposed skin and forms unpigmented lesions, whose diagnosis is usually difficult. The three most frequent locations of desmoplastic melanomas include head and neck (53%), extremities (26%), and trunk (20%). Desmoplastic melanoma has a propensity for neurotropism and is frequently associated with nerve invasion. Patients with desmoplastic melanoma have a slightly better prognosis than other melanomas. At the histological level, these tumors are characterized by the proliferation of spindle-shaped cells embedded in an abundant desmoplastic stroma and are difficult to diagnose. Two types of desmoplastic melanomas are described: (a) *mixed desmoplastic melanoma*, when the desmoplastic melanoma represents <90% of the total melanoma tissue; and *pure desmoplastic melanoma*, when the desmoplastic melanoma represents >90% of total melanoma tissue. Recent studies have provided a characterization of the genetic abnormalities occurring in desmoplastic melanoma (Figure 5): *BRAF* and *NRAS* mutations were absent; *NF1* (52%) and *TP53* (48%) mutations were particularly frequent; genes involved in MAPK pathway, cell-cycle, TP53 pathway, and epigenetic control are frequently mutated [152]. Interestingly, a novel alteration was observed in desmoplastic melanomas, consisting of the recurrent (14.5% of cases) promoter mutation of the NFKB Inhibitor Epsilon (*NFKBIE*) gene, encoding for an inhibitor of NFκB. Importantly, desmoplastic melanomas displayed a high mutation rate (with a median of 62 mutations per megabase). The mutation signature strongly implicates UV-radiation as the dominant mutagen responsible for desmoplastic melanoma generation [152]. The high mutational burden observed in desmoplastic melanoma is of significance for patient response to immunotherapy: in fact, a higher overall mutational load determines the expression of more neoantigens and a greater sensitivity to anti-tumor immunotherapies compared to those based on anti-PD1/anti-PDL1 antibodies [153]. Immunohistochemical studies have shown that about 50% of desmoplastic melanomas express PDL1 and mostly pertain to the mixed histological variant [154]. 

The frequency of some genetic alterations is different in pure compared to mixed desmoplastic melanoma. Thus, NF1 mutations were more frequent in desmoplastic than in non-desmoplastic melanoma (69% vs. 54%); furthermore, *NF1* mutations were more frequent in pure desmoplastic melanoma than in mixed desmoplastic melanoma (80% vs. 56%); finally, non-head desmoplastic melanomas are more frequently *NF1*-mutated than head and neck-localized melanomas (88% vs. 55%) [155]. Interestingly, most *NF1* mutations result in a truncated/absent neurofibromin protein. Finally, *TERT* promoter mutations were more frequent in mixed than in pure desmoplastic melanoma subtypes (54% vs. 23%) [156].

It is important to underline the high frequency of *NF1* mutations among desmoplastic melanomas. In this context, it is important to underline that desmoplastic melanomas and malignant peripheral nerve sheath tumor (MPNST) are the two tumors displaying the highest frequency of NF1 mutations; interestingly, these two tumors are cytologically and histologically similar [157]. NF1 mutational loss in desmoplastic melanoma is often associated with concurrent mutations in RASopathy genes; these concurrent mutations may act synergistically in melanoma development [90,158].

The diagnosis of desmoplastic melanoma is often difficult, and this tumor must be differentiated from neufibroma, spindle cell sarcoma, schwannoma, dermatofibroma, fibromatosis, and scar. At the immunohistochemical level, positivity for SOX10 is an essential marker to distinguish desmoplastic melanoma from these other tumors [159,160].

### 3.9. Epigenetic Changes in Cutaneous Melanoma

The studies carried out in recent years have highlighted the complexity of genetic somatic changes occurring in various types of melanoma. Only recently has the attention been addressed to the numerous epigenetic changes occurring in melanomas. The study of these changes is important because may help to better understand melanoma pathogenesis and may also offer the opportunity to identify new therapeutic targets. 

The epigenome consists of a wide spectrum of biochemical modifications, consisting of DNA methylation and histone marks, which associate with dynamic changes in the rate of gene expression and consequent changes in the rate of gene expression and consequent modifications in various biochemical pathways and cellular processes.

Cutaneous melanoma offers a unique model to explore the dynamic changes in epigenetic regulation as it relates to tumor progression. In this context, a fundamental observation was made by Lian and coworkers in 2012 that loss of DNA methylation at position 5 of the cytosine (5-mC) is an epigenetic hallmark of melanoma [129]. This conclusion is strongly supported by the observation that loss of 5-hmC correlated with melanoma progression, 5-hmC levels being much higher in benign nevi than in primary or metastatic melanomas [83]. Downregulation of IDH2 and TET family enzymes is one of the main mechanisms responsible for 5-hmC loss in melanoma [83]. Importantly, the expression of active TET2 or IDH2 into melanoma cells restores the 5-hmC landscape, inhibits melanoma proliferation, and increases survival in animal models [83]. The association between the loss of 5-hmC levels and malignant melanoma transformation was also supported through the study of proliferative nodules arising with congenital nevi; these nodules display some atypical features and predispose to malignant transformation, but were basically benign and exhibit—in contrast to melanomas—high 5-hmC levels and high TET2 and IDH2 activity [161]. TET2 and TET3 are silenced in melanoma cells by epigenetic mechanisms triggered by TGF-β and mediated by DNA methyltransferase 3A (DNMT3A); these events play a functional role in the epithelial-to-mesenchymal transition (EMT) and in metastatic processes of melanomas [162].

Other studies have provided evidence that some tumor suppressor genes, such as *PTEN*, *p16* (inhibitor of CDK4/6), *p14* (inhibitor of MDM2), RAS-association domain family 1 (*RASSF1A*) (cell cycle regulator), and *O*-6-Methyguanine-DNA Methyltransferase (*MGMT*) (a regulator of DNA repair) are silenced in melanoma cells through epigenetic mechanisms involving promoter hypermethylation [163]. Interestingly, CpG hypermethylation determines downregulation of the MITF—a key regulator of melanocyte development [164]. A comprehensive DNA methylation study of melanomas at various stages of development identified some genes specifically involved in tumor development (*HOXA9*) or progression (*TBC1D16*) [165].

Fiziev and coworkers have performed a systematic epigenomic analysis of melanomas at various stages of development, focused on the evaluation of various types of histone modifications occurring during tumorigenesis, and discovered that: chromatin states harboring acetylation and H3K4me2/3 are lost in melanoma cells; loss of histone acetylations preferentially occurred on regulatory regions proximal to specific cancer-regulatory genes involved in melanoma development; importantly, restoration of acetylation levels on deacetylated loci by histone deacetylase inhibitors selectively blocked hyperproliferation of melanoma cells [166].

Interestingly, a recent study based on the analysis of the transcriptome changes occurring in the transition from benign states to early-, intermediate-, and late-stage tumors allowed the identification of a high-risk subgroup of melanomas characterized by the deregulation of various genes regulating epigenetic machinery (“epigenetic gene signature”) and of the *TP53* gene family (*TP53, TP63,* and *TP73*) [167]. This subtype was characterized by poor overall survival and the enrichment of cell cycle genes [167].

### 3.10. Gene Expression Classification of Cutaneous Melanoma

In parallel to studies that have classified melanomas according to their spectrum of genetic alterations, other studies have explored the gene expression profile of cutaneous melanomas and have analyzed the possible link with the various genetic alterations. Basically, these studies have shown that melanoma gene expression patterns are determined by the expression of defined sets of genes, mainly represented by melanocyte differentiation and mitotic genes and by the genes expressed in the surrounding stromal cells or in the infiltrating immune cells. Few classifications of melanomas according to gene expression profiling have been proposed. Before analyzing these various gene expression-based classifications, it is important to point out that these classifications are not overlapping with the TCGA molecular classification of melanomas based on the mutational status for BRAF, NRAS, and NF1. 

In 2010, Jonsson and coworkers proposed a classification of cutaneous melanoma in four expression-based subtypes: the *high immune* subtype characterized by the elevated expression of immune genes; the *normal-like* (stromal) subtype, characterized by genes expressed in the surrounding normal cells; the *MITF-high pigmentation*, characterized by elevated expression of cell-cycle genes; the *MITF-low proliferative* subtype, characterized by a high expression of cell-cycle genes and a low expression of melanocyte differentiation genes [168]. This classification was based on the analysis of stage IV melanoma patients and was confirmed through the analysis of primary melanomas [169,170] and stage III melanoma [171]. Importantly, these molecular subtypes were predictive of outcome and response to therapy. Thus, the study of stage III melanoma patients showed that an increased risk of developing distant metastases was observed in the pigmentation and proliferative subtypes, as compared to the high-immune response group; furthermore, in patients receiving targeted therapy, melanomas resistant to targeted therapy were enriched in the MITF-low proliferative subtype [171].

The TCGA study on genomic characterization of cutaneous melanoma reported three melanoma gene expression subtypes: the immune group characterized by increased expression of immune genes; the keratin group characterized by elevated expression of keratin, pigmentation, and epithelial genes; the MITF-low expression displayed low expression of melanocyte differentiation genes and activation genes involved in nervous system development [89].

The comparison of these two gene expression classifications showed that: the TCGA immune group consisted of the Jonsson high-immune group (88%) plus 55% of the MITF-high pigmentation group samples; the TCGA keratin group consisted mainly of the Jonsson normal-like and MITF-high pigmentation groups; finally, the MITF-low TCGA group contained 76% of the Jonsson proliferative subtype [172]. Interestingly, all these studies provided evidence that the immune groups (immune and high-immune) had favorable survival rates compared to the other groups.

Several recent studies have explored gene expression profiles on subpopulations of melanoma patients characterized by the sensitivity or resistance to a given treatment. Thus, Lardone and coworkers have compared gene expression profiles of favorable outcome and poor outcome melanoma patients [173]. The results of this comparative analysis led to the identification of a “favorable outcome signature” of 228 genes, enriched in genes related to the immune function—particularly T cell-associated genes and B cell-associated genes [173]. Another study evaluated the association of gene expression profiling with progression-free survival outcomes of BRAF^V600^-mutated melanoma patients treated with vemurafenib alone or in combination with cobimetinib [174]. In these patients, two gene signatures were identified: cell cycle and immune. Cell cycle signature was associated with a poor response to vemurafenib treatment; however, in cobimetinib combined with vemurafenib-treated patients, both cell cycle and immune signature subgroups had comparable progression-free survival [174]. Hugo and coworkers analyzed the transcription features of metastatic melanoma responding or not to anti-PD1 therapy and observed that innately resistant tumors display a transcriptional signature, defined as innate anti-PD-1 resistance (IPRES), showing simultaneous over-expression of genes involved in the regulation of mesenchymal transition, cell adhesion, extracellular matrix remodeling, angiogenesis, and wound healing [175]. It is of interest to note that treatment with MAPK inhibitors induces a similar signature in melanoma cells [175].

In addition to the analysis of gene expression profiles, other studies evaluated the pattern of DNA methylation in cutaneous melanomas and tried to classify these tumors according to this pattern. Through the analysis of DNA methylation at CpG dinucleotides, Lauss and coworkers identified three different melanoma groups and validated these groups in independent data sets. One group was similar to normal melanocytes and displayed hypermethylation of a developmental promoter set, genome-wide demethylation, increased proliferation, and activity of the Switch/Sucrose Non-Fermentable (SWI/SNF) complex; a second group had a methylation pattern similar to stromal and leukocyte cells, overexpressed immune signature, and had improved survival; a third group had intermediate methylation levels and expressed both immune and proliferative signatures [176]. The first group was associated with a negative prognosis and was characterized by the expression of genes that promote the cell cycle, of genes that directly modify CpG methylation, and by the up-regulation of several members of the SWI7SNF complex [176]. Wouters and coworkers have performed a comprehensive study of DNA methylation of cutaneous melanomas at various stages of tumor development, and this analysis identified and validated biomarkers for melanoma development (such as *HOXA9* DNA methylation) and tumor progression (such as TBC1 Domain Family Member 16 (TBC1D16)) and discovered a prognostic signature with clinical applicability, involving the identification as biomarkers Paraoxonase 3 (PON3), Oligodendrocyte Transcription Factor 3 (OLIG3), and Mesenchyme Homeobox 2 (MEOX2), whose methylation was associated to negative prognosis [165]. Low Ovo-like Transcxriptional Repressor 1 (*OVOL1*) expression was also identified as a negative prognostic factor [165,177].

### 3.11. Intratumor Heterogeneity of Melanoma

Several recent studies have investigated the important problem of intra-patient tumor heterogeneity. The study of intra-patient heterogeneity is of fundamental importance not only for the understanding of the mechanism of tumor evolution at a clonal level, but also for the fundamental implications at a therapeutic level.

The problem of intra-patient tumor heterogeneity was assessed at two levels: at the level of various regions of the primary tumors, and at the level of tumor metastases. Initial studies have provided preliminary evidence that multiple metastases from primary cutaneous melanomas may display heterogeneous genomic and epigenomic patterns [178]. Targeted gene sequencing and gene expression microarray analysis provided evidence about molecular and genetic diversity in the metastatic process of melanoma [179]; the analysis of primary tumor and distant metastases showed the acquisition of new mutations in the individual metastases [179]. BRAF and NRAS mutations present in the first metastasis were always preserved in subsequent metastases [179]. These findings provided evidence of the continued evolution of individual tumors following divergence from a common parental clone [179]. Sanborn and coworkers have analyzed metastasis formation at a single-cell level, providing evidence that in most patients, genetically distinct cell populations in the primary tumor metastasize in parallel to different anatomic regions, rather than sequentially from one site to another [180]. Importantly, in a portion of melanoma patients, individual metastases are founded by multiple cell populations of the primary tumor that were genetically distinct [180].

Harbst and coworkers have explored intra-tumor heterogeneity at the level of various regions of primary tumors; this study provided evidence about the existence of an intra-tumor heterogeneity of somatic mutations, DNA copy number, and transcriptomic changes: particularly, 3–38% of somatic mutations were not identified in all regions of the individual tumors [181]. The level of intra-tumor heterogeneity observed in melanoma is only moderate, compared to other tumors: in fact, only 12% of cancer driver mutations are affected by intra-tumor heterogeneity, compared to levels much higher in other tumors, such as renal cell cancer [181]. Mutations in *BRAF* or *NRAS* are always ubiquitous events [181]. Importantly, patients harboring a high degree of intra-tumor heterogeneity were associated with more aggressive disease progression, thus suggesting that tumor heterogeneity promotes tumor progression [181].

As mentioned above, tumor heterogeneity is one of the main causes of drug resistance. Two studies provided evidence about a mechanism of primary resistance to MAPK inhibitors occurring in a portion of *BRAF^V600^*-mutant melanoma [182,183]. In fact, although MAPK inhibitors show clinical benefit in some *BRAF^V600^*-mutant melanomas, about 20% of these tumors are intrinsically resistant to these drugs. BRAF inhibitor-resistant and BRAF inhibitor-sensitive melanomas display distinct transcriptional profiles: in fact, drug-sensitive cells display high MITF expression, associated with low NF-kB signaling and low receptor tyrosine kinase AXL, while drug-sensitive cells display the opposite phenotype, with low MITF expression, associated with high NF-κβ and AXL activity [182,183]. Ennen and coworkers performed an analysis of the MITF-high and MITF-low transcriptional profiles at the level of single melanoma tumor cells isolated from various lesions of five different melanoma patients, and reached two important conclusions: single-cell expression analysis revealed inter- and intra-tumor heterogeneity in primary melanoma; importantly and interestingly, primary melanomas comprise subpopulations of tumor cells co-expressing genes of the MITF-high and MITF-low signatures at the level of single cells [184]. 

Li and coworkers recently reported the analysis of intra-tumor heterogeneity by multiregional sequencing and clonal dynamics in a case of mucosal melanoma of the esophagus—a very rare localization of mucosal melanomas [185]. The characterization of intra-tumor heterogeneity of this patient showed a pattern of tumor evolution compatible with a branching evolution model, with an ancestor clone evolving along different trajectories and diversified into primary tumor subclones and early and late metastatic clones [185]. Particularly, *BRAF* and *KRAS* mutations, as well as *CDKN2A* biallelic inactivation, were observed in trunk clones, whereas some clinically actionable mutations—such as *PIK3CA* and *JAK1*—were detected in branch clones [185]. Ancestor clones evolved along three different trajectories: clade 1 fostered metastatic clones that displayed some metastatic-related genomic alterations, such as *PIK3CA* and *ARHGAP26* mutations, as well as chromosome 13 arm-level deletion; clade 2 fostered branch-specific subclones, characterized by branch-specific alterations such as *JAK1* mutations and *PTEN* deletion; clade 3 represented a vertical region of tumor cell transmission from the primary tumor [185]. 

The study of the mechanisms of resistance of melanoma cells to vemurafenib helped to understand the basis of a peculiar melanoma heterogeneity, not directly related to genetic mechanisms. These studies defined a mechanism of drug resistance related to a phenotypic—not heritable—heterogeneity of melanoma cells. In fact, in a recent study Sheffer and coworkers provided evidence that even in a sensitive melanoma cell population, there is a very small cell population of vemurafenib-resistant cells characterized at transcriptional level by the coordinated expression of resistance genes [186]. The addition of the drug induces an epigenetic reprogramming in these cells, triggered by a loss of SOX10-mediated differentiation, followed by the activation of new signaling pathways, induced by the activity of the transcription factors JUN and/or AP-1 and TEAD [186]. 

## 4. Animal Models of Melanoma

The development of genetically engineered melanoma models (GEMMs) did take considerable advantage of the progressive understanding of the current genetic abnormalities occurring in melanomas. Most of these models were based on the development of transgenic animal models. Two recent review papers have given a detailed analysis of the mouse models of melanoma [187,188]. Targeted deletion of the *CDKN2A* locus induced the development of various tumors, but not of melanomas. The role of RAS in melanoma development was investigated through the melanocyte-specific expression of mutated *HRAS*: tyrosinase-driven expression of *HRas^V12G^* in mouse melanocytes failed to induce melanoma development. However, ultraviolet radiation therapy (UVR) treatment in these animals induced melanoma with long latency: interestingly, *CDKN2A* was found to be deleted in developing tumors [189]. Another *RAS* mutation, *NRas^Q61K^*, in melanocytes induced melanoma development with a low incidence rate and only after a long latency; cross-breeding with *CDKN2A* knockout mice increased the incidence of melanomas, with a reduced latency period [190]. 

Other studies have explored the effect of the induction of BRAF^V600E^ expression, providing conflicting evidence about the capacity of this oncogene alone to induce melanoma development [191,192]. The observation that the benign nevi carry *BRAF^V600E^* mutation suggests that *BRAF* mutation is not sufficient alone to drive melanoma formation; in line with this observation, most of the mouse studies based on BRAF^V600E^ expression indicate the development of melanocytic nevus-like hyperplasia [192]. Other studies have shown that *BRAF^V600E^* cooperates with *PTEN* loss to induce the formation of metastatic melanoma. In this genetic setting, the development of melanoma was rapid and aggressive [193].

Particularly interesting are the results obtained in the zebrafish model. In line with observations made in benign nevi, the simple expression of BRAF^V600E^ under the control of the melanocyte-specific *MITFA*-promoter failed to induce the formation of melanocytic tumors in zebrafish; however, when crossed into a *PT53* mutant loss-of-function background, these zebrafish develop nevi, and after several months, melanomas [194]. The fact that these p53/BRAF melanoma mice developed one to three melanomas only after several months strongly suggests that other molecular abnormalities—in addition to BRAF—are important for melanoma development. Using live imaging of transgenic zebrafish reporters of the crestin gene (normally expressed in embryonic neural crest progenitors, NCPs), evidence was provided that the *BRAF^V600E^*, TP53-deficient genetic background reactivates a state observed at the level of embryonic NPCs in melanocytes [194]. This transition indicates that a fate change occurs at the initiation of the melanoma tumorigenic process, orchestrated by the SOX10 transcription factor [194]. In line with this observation, enforced *SOX10* overexpression in melanocytes consistently accelerated melanoma formation, thus supporting that the NPC state induced by SOX10 is a prerequisite for melanoma transformation in this genetic setting [194].

The mouse melanoma models also offer the opportunity to identify the cell of the melanocyte lineage whose transformation determines melanoma formation. While it is evident that all melanomas—as well as other melanocytic neoplasms—derive from the neural crest-derived melanocytic lineage, it is unclear whether melanomas derive from the malignant transformation of melanoma stem cells or from differentiated melanocytes. In human skin, the large majority of melanocytes reside at the dermo-epidermal junction; the murine tail contains interfollicular melanocytes. Both human and mouse melanocytes also colonize hair follicles, where they contribute to melanin production of the hair shaft. The hair follicles undergo a process of cyclic expansion (known as anagen) and a process of regression (known as catagen): during the anagen phase, melanocyte stem cells located in their hair bulge undergo a process of expansion and partial differentiation first in transient amplifying cells and then in melanin-producing melanocytes; during the catagen phase, most of differentiated melanocytes undergo an apoptotic process [195]. At least in mouse, hair follicles are not the only source of melanocytes, in that mice tail skin lacking appendages is capable of maintaining melanocytes and a low number of amelanocytic melanocytes that may act as a reservoir of interfollicular melanocyte stem cells [196]. A recent study used mouse genetics, lineage tracing experiments, time-lapse imaging, and single-cell profiling techniques to identify the cells responsible for the generation of *BRAF^V600E^*-induced melanoma and to investigate the sequential cellular and molecular changes occurring in melanoma-initiating cells from the first stages of tumor in initiation to the late stages of melanoma development [197]. The results of this study showed a variable tumor susceptibility among different melanocyte subpopulations, with the highest melanomagenic capacity at the level of mature melanin-producing melanocytes [197]. These observations imply that mature melanocytes (at least in the context of these experimental conditions) are able to de-differentiate into cancer-initiating cells [197]. Moon and coworkers have used lineage-tracing of melanocyte stem cells in melanoma mouse models to define the cells of origin of these tumors [198]. These studies showed that UVB radiations induce mesenchymal stem cell activation and translocation through an inflammation-dependent process [198]. In the process of UVB-mediated melanocyte stem cell activation/translation and melanoma development, an essential role is played by the chromatin remodeling factor HMGA2 [198].

The development of mouse models of melanoma has played a major role in the understanding of the molecular mechanisms underlying melanomagenesis. It is important to point out that animal models of melanoma may also play a fundamental role in the study of environmental factors inducing melanoma formation. Thus, using mouse melanoma models it was shown that melanoma induction by ultraviolet A (UVA) radiation (320–420 nm) requires the presence of melanin pigment and is associated with oxidative DNA damage within melanocytes; in contrast, ultraviolet B radiation (280–320 nm) triggers melanoma formation in a pigment-independent manner with direct UVB DNA damage [2,199].

Patient-derived tumor xenografts (PDTXs) also represent a model for the study of human melanomas. PDTX models are superior to traditional cell lines because they maintain similarities to the tumors observed in patients [200]. This assay was particularly useful in the evaluation of the drug sensitivity of melanoma-defined subsets using clinically-relevant drug doses. Thus, using patient-tumor derived xenografts it was possible to evaluate the drug sensitivity of primary and metastatic melanomas in in vitro and in vivo three-dimensional environments [201]. The xenograft models were very useful in the study of the development of drug resistance to target therapy and in the definition of a possible switch therapy to bypass this resistance. Thus, PDTX models of melanomas resistant to BRAF inhibitors (vemurafenib) have been developed, and have been studied to explore the mechanisms of drug resistance [202,203,204]. Now, in some clinical trials the treatment of mice xenografted with the same tumor cells of patients undergoing clinical trials has been performed, and this parallel analysis will allow the evaluation of the capacity of mouse xenograft studies to predict the clinical response on the same tumor specimens.

## 5. Melanoma Cancer Stem Cells

Early evidences of the existence of stem cells within melanoma cancer samples came from a study by Fang et al. [205] reporting the presence of a cell subpopulation forming non-adherent growing spheres in melanoma metastases and melanoma cell lines when cultured in a medium suitable for human embryonic stem cells; these spheroids displayed tumorigenicity when xenografted in NOD/SCID mice [205]. Interestingly, this initial report provided evidence that the membrane CD20 antigen could represent a membrane marker for melanoma cancer stem cells. More recently, it was shown that the targeted elimination of less than 2% subset of CD20^+^ melanoma cells in a transplantation model could confer lasting eradication of the tumor lesion in the immunodeficient animal [206,207]. These observations implied CD20^+^ melanoma cells as a main driver of melanoma inhibition and progression. Given these observations, attempts were made to eliminate these CD20^+^ cells first in experimental models of melanoma and then in melanoma patients. First, Schmidt and coworkers carried out experiments in a preclinical melanoma xenograft model, reporting a marked inhibition of growth and relapse of highly tumorigenic melanoma cells through the targeting of CD20^+^ tumor cells with autologous T cells genetically engineered to express a chimeric CD3ζ/CD20 antigen receptor [208]. Taking advantage of these observations, Schlaak and coworkers treated a progressing, chemotherapy-refractory metastatic melanoma patient with the anti-CD20 monoclonal antibody rituximab (intra-lesional administration), resulting in a lasting remission, accompanied by a decline of the melanoma serum marker S-100 and in a switch of serum cytokines from a T helper-2 to a pro-inflammatory helper-1 cell profile [209]. In another study, nine melanoma patients with stage IV disease who had been rendered apparently disease-free by standard therapies received treatment with rituximab (anti-CD20 monoclonal antibody) [208]. After a median observation of 42 months, six out of nine patients are still alive and five of them are recurrence-free [208]. These observations suggest a possible clinical utility of anti-CD20 treatment in CD20^+^ melanomas. These initial clinical observations need to be validated in carefully designed clinical trials. Recently, it was reported the development of CD20 antibody-conjugated immunoliposomes for targeted therapy of melanoma cells, including melanoma cancer-initiating cells [210]. It is important to note that the CD20 antigen is also expressed on tumor-associated B cells (TABCs), which may represent up to 30% of immune cells infiltrating melanomas [211]. TABCs play a positive role in tumor growth and, particularly in tumor chemoresistance through the release of the growth factor insulin-like growth factor 1 (IGF-1), mediating the appearance of heterogeneous tumor cell populations resistant to BRAF and/or MEK inhibitors [211]. Importantly, IGF-1 also stimulates the expression of cancer stem cell markers on melanoma cells, including CD20, CD133, and CD271 [211]. In line with these findings, the resistance of melanomas to BRAF and/or MEK inhibitors is associated with increased CD20 and IGF-1 transcripts in TABCs [211]. Furthermore, preliminary clinical data deriving from a phase I trial in therapy-resistant melanoma patients showed initial evidence of anti-tumor activity though B-cell depletion induced by anti-CD20 antibody [211].

A subsequent study by Monzani et al. showed that CD133 positivity in metastatic melanoma patients was associated with increased tumorigenicity as assayed by xenotransplantation in NOD/SCID mice [212]. It is important to note that tumor formation in these mice was observed only 40–50 days after transplantation [212]. However, in these studies it was not shown whether CD133^+^ melanoma cells were capable of self-renewal. Interestingly, ABCG2, one of the membrane transporters responsible for multi-drug resistance, was found to be expressed on these CD133^+^ cells. Some additional data support the idea that CD133 expression in melanoma could be related to the tumoral stem cell compartment. In fact, a positive correlation between CD133 expression and melanoma progression was observed in immunohistochemical staining of tumor tissue specimens [213]. Furthermore, in another study it was shown that downregulation of CD133 expression resulted in a decrease of melanoma cell growth and capacity to develop metastasis, thus suggesting a role for this membrane antigen in tumor progression [214].

In a subsequent study, Schatton et al. have shown that a rare subpopulation of melanoma cells—identified based on the positivity for the expression of the multidrug transporter ABCB5—was responsible for tumorigenicity in NOD/SCID mice [215]. In this study, it was estimated that the frequency of tumorigenic cells was 1/1,000,000, and it was enriched about 10 times in ABCB5^+^ cells [215]. ABCB5^+^ melanoma cells were shown to be capable of tumorigenesis, self-renewal, and differentiation into a heterogeneous cell population when primary patient-derived tumor cells were serially transplanted into NOD/SCID mice. In this study, it evidence was also provided that there is a positive correlation between ABCB5 immunoreactivity and the clinical evolution of melanoma: in fact, ABCB5 expression was higher in primary melanoma than nevus and lymph node metastasis than primary melanoma [215]. Subsequent studies have provided additional confirmatory evidence about the existence of a progressively increasing expression of ABCB5 following disease evolution. Furthermore, the induction of differentiation of melanoma cells was associated with a decreased ABCB5 expression [215]. Another study showed that the membrane transporter conferring multi drug resistance (MDR-1) was expressed in a minority of melanoma cells displaying in vitro clonogenic properties; interestingly, these cells co-express ABCB5 [216]. Finally, a very recent study showed that melanoma chemotherapy leads to the selection of ABCB5-expressing cells [217]. Studies carried out in vitro and in patients’ sample specimens provided evidence that ABCB5-expressing cells selectively survive when melanoma cells are exposed to melanoma chemotherapeutics, including vemurafenib, the BRAF inhibitor used for treatment of BRAF V600E melanomas [217]. These observations suggest that anti-melanoma chemotherapy might participate in the acquisition of chemoresistance by selecting tumor cell subpopulations expressing ABCB5 [218].

Frank et al. reported that ABCB5^+^ melanoma cells co-express CD133 in vitro [219]. Immunohistochemical analyses have shown that CD133^+^ and ABCB5^+^ subpopulations are co-localized in human melanomas at the level of perivascular niches that contain VE-cadherin-positive melanoma cells forming vessel-like channels—a phenomenon called vascular mimicry [220]. CD133 knockdown reduced the tumorigenic potential and the capacity to form VE-cadherin^+^-like channels [220]. According to these findings it was proposed that melanoma cancer stem cells (CSCs) drive tumor growth by promoting vascular mimicry and the formation of a specialized perivascular niche in melanoma.

Additional studies have shown that ABCB5^+^ melanoma cells overexpress the vasculogenic differentiation markers CD144 and Tie1 and are associated with vasculogenic mimicry. In addition to these markers, ABCB5^+^ melanoma cells also express VEGF-R1, which plays a role in the control of the proliferation and vasculogenic mimicry of these cells [221]. In fact, it was shown that ABCB5^+^, but not ABCB5^−^ melanoma cells express VEGF-R1 [221]; in vitro studies have shown that VEGF induced CD144 expression in ABCB5^+^ cells. Importantly, VEGF-R1 knockdown reduced ABCB5-induced tumor growth and blocked the development of ABCB5^+^ vascular mimicry morphology [221]. This role of VEGF-R1 in the control of the growth of melanoma CSCs also opens the way to some possible new targeted therapies [222]. In addition to VEGF-R1, also RANK (receptor activator of NF-kB) has been shown to be expressed on ABCB5^+^ melanoma-initiating cells, thus suggesting a possible role of this receptor in maintaining melanoma CSCs [222].

Given the expression of ABCB5 transporter on melanoma CSCs, it is not surprising that the Rh123 dye efflux assay was used as a tool to isolate these cells from tumoral specimens. Thus, Tpuil and coworkers isolated low Rh123-retention cells from melanoma metastatic lesions and demonstrated that a small subset of these cells display stem cell-like activities such as the capacity of self-renewing, to form melanospheres. Interestingly, at a molecular level these cells express ABCB5, HIF-1α, and the pluripotency transcription factor Oct4. Through a set of experiments these authors provided evidence that melanoma CSCs may exist in three different states: (a) quiescent cells, with long-term growth capacities; (b) slow-growing, slow-cycling melanoma stem cells; (c) actively proliferating stem cells directly feeding the cycling tumor mass [223]. The PI3K/AKT pathway plays an essential role in the control of the quiescence of Rho123^low^ cells [223].

It is important to note that the optimal detection of ABCB5 at the level of melanoma cells require the isolation of these cells through techniques that do not involve the use of trypsin. The expression of ABCB5 on melanoma cells was confirmed through the immunohistochemical analysis of primary tumor tissues [224]. Consistent with a possible functional role of ABCB5 in the maintenance and/or biological activity of melanoma-initiating cells, ABCB5 genetic variation was associated with melanoma risk [225]. A recent study provided evidence that ABCB5 might provide a functional link between melanoma-initiating cell maintenance, multi-drug resistance, and tumor growth in malignant melanoma. In fact, it was shown that in melanoma-initiating cells ABCB5 controls UK-IL1-β secretion, which acts to maintain slow-cycling, chemoresistant cells through a signaling pathway mediated by the IL-1/βIL-8/CXCR1 signaling axis [226]. On the other hand, ABCB5 blockade induced cellular differentiation of melanoma cells, reversed their resistance to multiple chemotherapeutic drugs, and impaired their tumorigenesis in vivo [226]. A recent study provided evidence that ABCB5 promotes metastatic activity by melanoma cells; in fact, ABCB5^+^ cells have more metastatic activity than ABCB5^−^ cells, this difference being related to a higher migratory capacity of ABCB5^+^ cells [227]. In experimental models, ABCB5 knockdown reduced the migratory capacity of melanoma cells and their capacity to metastasize in vivo in mouse models [227]. In melanoma tissues, ABCB5 and NF-κβ expression are positively correlated, and ABCB5 was shown to activate NF-κβ activity, promoting p65 protein stability [227].

However, Quintana et al. modified some parameters of the xenotransplantation assay and observed that the frequency of tumor-initiating cells in melanoma was markedly higher that that reported in previous assays [228]. Particularly, they modified the recipient immunodeficient mice (in fact, they used NSG mice (NOD/SCID/IL-2Rγ^−^ mice), more immunodeficient than NOD/SCID mice), the immediate extracellular environment (the tumor cells were resuspended in Matrigel), and the assay duration [228]. Using these experimental conditions, the frequency of cancer stem cells was evaluated in six metastatic melanoma patients with advanced disease reporting an average frequency corresponding to 25% [221]. More recent reports confirmed a high frequency of tumorigenic capacity of melanoma cells [229,230].

Two recent studies further provided new interesting information on melanoma CSCs. Thus, Boiko et al. showed that CD271-expressing cells are able to initiate and maintain melanoma cell growth in vivo [231]. Interestingly, the frequency of CD271^+^ cells varied from 2.5% to 41% in the various melanoma samples, thus suggesting that tumor-initiating cells are not rare in these tumors. It is important to note that in this study it was shown that the tumor initiating capacity was not exclusively limited to CD271^+^ cells, but in some cases it also extends to CD271^−^cells [231]. An additional important observation coming from this study was that xenograft tumors had higher CD271^+^ cell fractions than the initial tumor, indicating that the xenografting process favors the selection of highly tumorigenic CD271^+^ cells. These observations were confirmed in a subsequent study, showing using NSG mice as nude mice recipients that both CD271^+^ and CD271^−^ cells induce the development of tumors. However, CD-271 positive fractions, but not CD271 negative fractions could be passaged several times in the nude mice, thus supporting the idea that the former ones, but not the latter ones, are true cancer stem cells [232]. In fact, the CD271-positive cell fraction displayed the capacity to self-renew and to repeatedly induce tumor formation even after six passages into immunodeficient mice; in contrast, CD271-negative cells exhausted with time and could be propagated in vivo for a maximum of four passages [232]. Recent studies provided evidence that CD271 is a marker of a stem cell-like population in uveal melanoma [167]. Interestingly, these cells exhibit the property of vascular mimicry (i.e., they are capable of forming peculiar vascular structures) [233].

CD271^+^ cancer stem cells lack the expression of melanocytic markers, and this finding is not surprising because these cells are dedifferentiated. A recent study addressed the problem of melanoma dedifferentiation. Thus, Kumar et al. have shown that the expression of Oct4 transcription factor into melanoma cell lines promoted dedifferentiation of melanoma cells to CSC-like cells: these dedifferentiated cells displayed a reduced expression of melanocytic markers and acquired the capacity to form tumor spheroids, and in xenotransplantation assays these cells displayed increased tumorigenicity [234]. CD271 expression on melanoma cells seems to be required for the induction of some stemness properties. This conclusion was reached through CD271 knockdown experiments showing a reduced expression of the transcription factors SOX10 (whose expression was required for maintenance of melanoma CSCs) and MITF (required for melanoma proliferation and invasiveness) [235]. Furthermore, CD271 expression was observed in all primary melanoma tumors (primary sites and metastases) analyzed, and its expression never co-localized with CD133 expression [235]. These findings were challenged by other studies indicating that CD271 expression on patient melanoma cells is unstable and not linked to tumorigenicity: in fact, CD271^+^ and CD271^−^ primary melanoma cells were found to be similarly tumorigenic using various specific assays [236]. Furthermore, variable CD271 expression patterns were observed in sibling analysis of CD271^+^ - derived cells, thus indicating that CD271 expression is unstable [236]. In a recent study, Ngo and coworkers reported an experimental strategy to effectively suppress melanoma metastasis in patient-derived xenografts, based on the simultaneous targeting of the tumor microenvironment using an anti-CD47 mAb (CD47 is expressed on all melanoma clinical samples, its expression being higher in metastatic than in primary tumors) and of melanoma cells using an anti-CD271 mAb [237]. This double antibody treatment was associated with a drastic change in the tumor microenvironment coupled with an increased density of differentiated macrophages and fewer inflammatory pro-metastatic macrophages [237].

Cheli and coworkers have characterized a slow-growing population of melanomas exhibiting properties of melanoma-initiating cells [238]. This cell population, observed in both melanoma cell lines and primary melanoma metastases, is characterized by the low expression of the master regulator of melanocyte differentiation—the MITF [238]. The low expression of MITF is functionally relevant for these cells, as supported by the observation that inhibition of MITF expression in melanoma cell lines increased the tumorigenic potential and the expression of stemness markers such as OCT4 and Nanog [238]. Ablation of these slow-growing cells from either melanoma cell lines or primary melanoma tumors greatly decreased their tumorigenic potential [238]. In a subsequent study, the same authors investigated the possible relationship between low-MITF-expressing cells and ABCB5^+^ and CD271^+^ cells [239]. They showed that CD271^+^ and ABCB5^+^ cells poorly overlap; low-MITF cells are enriched in CD271^+^, but not in ABCB5^+^ cells [240]. They also showed that only slowly-growing CD271^+^ cells, but not rapidly-growing CD271^+^ cells are highly tumorigenic [239]. Finally, they also observed that CD271 expression in vitro is unstable, in that CD271^+^ cells are rapidly converted to CD271^−^ cells [239]. CD271 expression in melanoma cells was related to the migratory properties [240] and to DNA damage response and drug resistance [241].

Another interesting observation comes from a study by Roesch et al., based on the identification of human melanoma cell subpopulations expressing the enzyme JARID1B. JARID1B belongs to the highly-conserved family of jumonji/ARID1 histone demethylases capable of removing mono-, di-, or tri-methyl groups from histone lysine residues. The pattern of expression and the biologic effects exerted by JARID1B in melanocytic cells have led to the suggestion that it may act as a suppressor in melanoma. JARID1B^+^ cells grow more slowly, were more tumorigenic, and generated a larger progeny than JARID1B^−^ cells [242]. A very intriguing observation was that the expression of JARID1B-positive cells was dynamic, in that JARID1B^+^ cells may be generated by JARID1B^−^ cells and vice-versa [242]. This observation may have important implications, including those related to therapies which target cancer stem cells.

Recently, using their very sensitive in vivo assay of cancer stem cells based on the use of highly immunodeficient mice, Quintana et al. have re-explored the tumorigenic capacity of various melanoma cell types [243]. First, they showed that 28% (range from 15% to 50%) of single melanoma cells were able to form tumors when injected into NOD/SCID IL2Rγ^null^ mice. These findings were confirmed for stage II, II, and IV melanoma patients and showed virtually unlimited tumorigenic capacity upon serial transplantation. None of 22 heterogeneously expressed membrane markers, including ABCB5 and CD271, were able to enrich for tumor-initiating cells [243]. Thus, both ABCB5^−^ and ABCB5^+^, as well as CD133^−^ and CD133^+^ cells exhibit the capacity to generate tumors exhibiting similar heterogeneity regarding CD133 expression. These findings indicate that melanoma cells possess an intrinsic phenotypic plasticity, which contrasts with both the cancer stem cell and clonal evolution models and may be attributed to reversible changes within tumorigenic cells, rather than to irreversible genetic and epigenetic changes that occurred and continuously occur in these cells.

Other studies have proposed additional biomarkers for melanoma cancer stem cells. Studies carried out in many cancers have shown that high aldehyde dehydrogenase (ALDH) represents a useful marker for identifying cells with properties of tumor-initiating cells. Initial studies carried out on melanoma cells indicated that ALDH activity does not select for cells with enhanced aggressive properties; furthermore, both ALDH^+^ and ALDH^−^ cells were shown to be able to induce tumor formation in vivo in immunodeficient animals [244]. In line with this observation, Amann et al. reported that melanoma cells display ALDH levels comparable to those observed in normal melanocytes [245]. In contrast to these findings, other reports suggested that melanoma ALDH^+^ cells displayed a higher tumorigenic activity in vivo compared to ALDH^−^ cells [246,247]. Particularly, using IL-2Rgamma^−/−^ NOD/SCID mice as recipient animals, Boonyaratanakomkit et al. showed that ALDH^+^ cells were enriched about 100-fold in tumorigenic cells compared to unfractionated cells [245]. On the other hand, Santini et al. reported that HEDGEHOG-GLI signaling was essential to drive the self-renewal of melanoma ALDH^+^ cells [247]. A very recent report reinforced the concept that ALDH may represent a useful and important biomarker of melanoma cells. In fact, Luo et al. showed that melanoma ALDH^+^ cells (predominantly expressing ALDH1A1 and ALDH1A3 isozymes) are more tumorigenic than ALDH^−^ cells [248]. Importantly, the silencing of ADLH1A reduced the tumorigenic potential of melanoma ALDH^+^ cells [241]. These findings implicate ALDH isozymes not only as biomarkers of melanoma CSCs, but also as therapeutic targets for human melanoma [248]. A recent study provided evidence that ALDH1A3 is epigenetically upregulated in nevi and melanoma [249]. Interestingly, the ALDH1-specific inhibitor DIMATE or depletion of ALD1A promoted the accumulation of apoptogenic aldehydes, with consequent induction of cell death and tumor growth inhibition both in vitro and in vivo in patient-derived xenograft assays [249]. Interestingly, the ALDH1A inhibitor targets both the bulk melanoma population and the slow-cycling tumorigenic/chemoresistant cells [249]. 

Studies carried out on melanoma cell lines suggest that CXCR6—a co-receptor for a cytokine—could represent a useful membrane biomarker for the isolation of melanoma cells capable of initiating the formation of tumors exhibiting a more aggressive behavior than tumors initiated by ABCG2-positive cells in immunodeficient animals [250].

On the other hand, evidence was provided that the expression of tenascin—a secreted extracellular matrix protein—is strongly upmodulated in melanoma cells grown as 3D spheres, compared to tumor cells grown as adherent cells. Knockdown of tenascin-C elicited a dramatic decrease of the ABCB5-positive side population of melanoma cells [203], and markedly increased the sensitivity of melanoma cells to doxorubicin [251].

Some studies have explored the expression of markers of neural crest stem cells in melanomas. Particularly, some studies have explored the expression and the function of SOX10, a transcription factor belonging to the HMG-box transcription factor family expressed at the level of neural crest stem cells. SOX10 plays an important role as a multipotency determinant in neural crest stem cells and also regulates the expression of some lineage-specific genes in melanocytes. Sox10 was found to be expressed in all melanomas. Importantly, *SOX10* silencing in human melanoma cells suppresses neural crest stem cell properties, counteracts proliferation and cell survival, and completely inhibits tumor formation in vivo [252]. A recent study partly clarified the molecular mechanisms through which SOX10 promotes melanoma development. This tumor promoting effect seems to be related to the capacity of SOX10 to repress the anti-tumorigenic program mediated by the activity of the related factor SOX9 [245]. *SOX10* inactivation induces a marked upmodulation of SOX9, with consequent induction of cell cycle arrest and melanoma cell death [253]. On the other hand, SOX9 upmodulation determines a marked decrease of SOX10 levels through the modulation of SOX10 gene transcription mediated by the binding of SOX9 at the level of the *SOX10* gene promoter [253]. These observations indicate the existence of an antagonistic relationship between SOX9 and SOX10 in promoting melanoma initiation [253]. Another mechanism through which SOX10 mediates melanoma development—in cooperation with MYC—is related to the induction of a lysosomal program of gene expression: melanoma cells exploit the development of lysosomal degradation pathways to accelerate tumor growth [254]. Among the lysosomal factors, a particularly relevant factor is the RAB7A GTPase, identified as a regulator of melanoma progression [254]. In addition to SOX10, SOX2—another member of the HMG-box transcription factor family—also plays an important role in the control of self-renewal and tumorigenicity of melanoma-initiating cells [255]. This conclusion was reached through experiments of *SOX2*-enforced expression and knockdown in melanoma cells [255]. 

Some studies have addressed the problem of melanoma cancer stem cells in the context of the dynamics of melanoma tumor cell populations. In fact, some studies have identified three types of melanoma cells with different phenotypes: cells expressing markers of differentiated melanocytes (up to the stage of pigment production); cells endowed with a high proliferative potential and with some invasive properties; and cells slowly proliferating with a stem-like phenotype [256]. The cells can switch from one phenotype to another. These three tumor cell populations can be defined according to MITF levels: cells with low MITF levels are G1-arrested stem-like cells endowed with a tumor-initiating potential and are highly-invasive, while high MITF levels are observed in cells that are able either to highly proliferate or to differentiate into pigment-producing cells [200]. These findings were corroborated by transcription profiling studies showing a low-expressing MITF cell population, low invasive potential, and low TGF-β signaling. According to these findings, it was hypothesized that a key event in cancer stem cell maintenance could consist of the events that maintain low MITF levels in these cells. A candidate regulator of MITF levels in cancer stem cells is represented by the transcription factor BRN-2, which is frequently overexpressed in melanoma cells and represses MITF transcription [257]. Interestingly, double staining experiments clearly showed that MITF and BRN-2 expression in melanoma cells is mutually exclusive [257]. BRN-2 and MITF expression was also detected in melanoma spheres obtained from melanoma cell lines: within each melanosphere, the expression of these two transcription factors was reciprocal [257]. It was also shown that in melanospheres BRN-2 acts as an activator of the NOTCH pathway, while MITF was a repressor of the NOTCH pathway [257]. Silencing of BRN-2 expression in melanoma cells resulted in a reduction of tumor spheres formation, thus indicating that high BRN-2 levels are required to sustain the properties of melanoma tumor-initiating cells [258]. The existence of these two stem cell populations is also supported by real-time intravital imaging of melanoma syngeneic tumors engineered to express a BRN2 promoter-GFP reporter, showing that high levels of BRN-2 promoter activity allows the identification of invasive melanoma cells and provides direct support to the occurrence in vivo of melanoma self-renewal and of frequent phenotypic switching from a stem cell-like condition (invasive and self-renewing cells) to a proliferative/differentiative condition and less-frequent phenotypic switching from a proliferative/differentiative condition to a stem cell-like phenotype [258]. The molecular mechanisms responsible for BRN-2 upmodulation in melanoma cells have been recently explored. In this context, one study provided evidence that BRN-2 expression is stimulated in response to the activation of MAPK: in fact, it was shown that the expression of the oncogenic BRAF^V600E^ mutant activates MEK, with consequent upregulation of BRN-2 levels, which in turn downregulates the cGMP-specific phosphodiesterase PRE5A with consequent increase in cytosolic free Ca^2+^, stimulating tumor invasivity [259]. In addition to being regulated by BRAF and β-catenin, the BRN-2 promoter is controlled by the transcription factor PAX3: the expression of PAX3 (and consequently of BRN-2) is strongly inhibited by PI3K inhibitors [260].

It is of interest to note that the large majority of stem-cell-associated markers of melanoma cancer stem cells identify a population of slow-cycling cells. There is growing evidence that the slow cycling phenotype in melanoma identifies a subset of cells associated with resistance to various types of treatments: these cells are characterized by a de-differentiated state, low MITF expression, activation of signaling pathways such as WNT5A and EGFR, and high expression of JARID1B protein [261]. The slow-cycling melanoma subpopulation display highly invasive properties, in part related to the high Serpin 2 [262] and CD36 [263] expression. CD36 identifies melanoma metastasis-initiating cells [263]. 

In spite the absence of specific membrane markers allowing the unequivocal identification of melanoma CSCs, some recent studies have tried to identify cell populations with stemness markers within the melanoma cell population. Basically, these studies aimed to demonstrate the expression of pluripotent transcription factors, Oct4 and Nanog, at the level of some melanoma cells. The inhibition of microphtalmia-associated transcription factor (MITF), a master regulator of melanocyte differentiation, increased the tumorigenic potential of melanoma cells, concomitantly with an upregulation of stem cell markers Oct4 and Nanog [264]. On the other hand, the enforced expression of Oct4 promoted the dedifferentiation of melanoma cells toward melanoma CSCs, associated with decreased expression of melanocyte differentiation markers, acquisition of multipotent differentiation capacity, acquisition of the expression of the membrane markers ABCB5 and CD271, resistance to chemotherapy, and increased tumorigenicity [265].

Other studies have provided some evidence that the genetic programs of cancer stemness and tumor invasiveness overlap in melanoma. In fact, Romano and coworkers recently reported that FKBP51—an immunophilin involved in the mechanism of chemoresistance which is markedly expressed in melanoma cells—from one side induced the expression of some stemness genes and from the other side promoted the activation of epithelial-to-mesenchymal transition, and improved the melanoma migration and invasion capacities [266].

A recent study reported the identification of a new potential membrane marker, Cripto-1, identifying a subpopulation of melanoma cells endowed with slow-cycling activity and with the capacity of forming tumors in immunodeficient mice. Cripto-1 is a membrane receptor pertaining to the EGF-related family, acting as a receptor for Nodal, a TGFβ-related morphogen. Cripto-1 plays an important role in the control of self-renewal of human embryonic stem cells, and is expressed at the cell surface level in a subset of melanoma cells. Given the important role played by Cripto-1 in the control of cells of the stem cell compartment and its limited cellular distribution at the level of the membrane of melanoma cells, it seemed interesting to isolate and evaluate the properties of Cripto-1 cells isolated from melanoma cancer tissues. Cripto-1 cells isolated from melanoma tissues displayed increased expression of Oct-4 and MDR-1 and showed only a moderate tumorsphere-forming capacity in vitro; however, when injected into immunodeficient mice, Cripto-1^+^ cells produced a first generation of slow-growing heterogeneous tumors. These tumors, however, originated a second generation of aggressive rapidly growing tumors when re-transplanted into immunodeficient mice [267]. These observations suggest that Cripto-1^+^ cells may contribute to the formation of a pool of slow-growing melanoma cells with cancer stem cell properties [267].

As mentioned above and as will be discussed in another section, a number of antibody-based therapeutics targeting the PD-1/PD-L1 axis have entered clinical development and have been approved for melanoma treatment. Interestingly, Kleffel and coworkers have recently shown that a variable and usually small proportion of melanoma cells express the PD-1 receptor on their surface; interestingly, the majority of PD-1^+^ cells co-express ABCB5 [268]. Importantly, PD-1^+^ cells isolated from melanomas promote tumorigenicity when inoculated into immunodeficient mice [268]. The activation of PD-1 present on melanoma cells with its ligand PD-L1 promotes melanoma proliferation [268]. According to these observations, it was concluded that anti-PD-1 blocking antibodies exert an anti-melanoma effect by two different mechanisms: triggering an immunologic response anti-tumor cells and inhibiting melanoma cell proliferation through the inhibition of melanoma PD-1^+^ cells [268].

The conclusion that may be tentatively derived from all these studies on melanoma cancer stem cells is that melanoma follows a model of tumorigenesis where virtually all tumor cells may have the potentiality to become tumorigenic due to their intrinsic plasticity and then to their capacity of acquiring stemness properties under appropriate conditions. These conclusions were mainly derived from the studies carried out in the laboratory of Dr. Morrison, definitively proving using NOD/SCID IL2Rγ^null^ mice as recipient animals that the frequency of tumorigenic cells from more than 30 patients with different stages and anatomical sites of disease was consistently high [269]. It is important to note that similar conclusions have been reached in studies carried out through transplantation of murine melanomas [270]. According to these findings, the melanoma must be considered as a neoplasia not hierarchically organized, which follows a dynamic model, in which subpopulations change depending on microenvironmental stimulations. Because of this tumor cell plasticity, it is evident that it is virtually impossible to identify cell membrane markers specific for cancer stem cells.

It is important to note that in contrast to these reports, other studies have suggested that melanomas follow the classical cancer stem cell model. The discrepancy between these studies could mainly be related to important technical differences, such as the methodology used to digest tumor specimens (in some studies tumor specimens were dissociated using either collagenase or collagenase/dispase, while in other studies a trypsin digestion step was added to collagenase digestion) for the isolation of cancer stem cells and the strain of immunodeficient mice used for xenotransplantation assays [271]. Another important difference between the various studies on melanoma CSCs concerns the site of inoculation of tumor cells into immunodeficient mice. In fact, in the large majority of these studies, tumor cells were inoculated subcutaneously, giving rise to the growth of an encapsulated tumor mass, not generating metastases; in contrast, Boiko et al. [231] inoculated tumor cells intradermally, generating metastasizing tumors.

It is very important to note that the studies on melanoma cancer stem cells and the identification of suitable strains of immunodeficient mice (NSG) for xenotransplantation assay have recently triggered the use of this experimental model to evaluate the metastatic potential of melanoma cells. Thus, Quintana et al. have shown that melanomas from 25 patients showed reproducible differences at the level of the rate of spontaneous metastases occurring after their engraftment into NSG, and that these differences correlated with clinical outcome. Stage III melanoma patients who developed distant metastases within 22 months also formed in mice tumors that rapidly metastasized, whereas stage III melanoma patients who did not form distant metastasis within 2 to 50 months metastasized slowly after tumor transplantation into immunodeficient mice [269]. The tendency to develop metastasis in mice correlates with the presence of circulating melanoma cells [269]. Therefore, these observations indicate that xenotransplantation of human melanomas into NSG mice offers an opportunity to study both cancer stem cells and the mechanisms that regulate the metastasis of human melanomas.

Finally, it is important to underline that the differentiation status of melanoma cells may also explain their sensitivity/resistance to immunotherapy. Adoptive cell transfer therapies based on cytotoxic T cells that target melanoma-specific antigens are able to induce disease regression in some patients with metastatic disease, but many of these patients relapse after their initial response. It was hypothesized that in these patients, tumor relapse could be related mainly to the development of a mechanism of immunological resistance, but recent evidence suggests a different explanation, related to the effects of immunotherapy in tumor differentiation status. In fact, Landsberg and coworkers studying an experimental melanoma model have provided evidence that the T-cell-driven-inflammatory response—mainly mediated by TNF-α—exerts an inhibitory effect on melanoma cell differentiation [272]. Because of these effects, melanoma cells dedifferentiate and become scarcely sensitive to T-cell-mediated cytolysis [261]. These findings suggest that the intrinsic phenotypic plasticity of melanoma cells, stimulated by an inflammatory microenvironment, contributes to tumor relapse after an initial response to T-cell-immunotherapy [272]. In line with these observations, Boiko and coworkers [231] also provided evidence that the CD271^+^ tumor-initiating melanoma cells are able to escape immune cell control. In contrast to these findings, a recent study by Giammaritoni and coworkers suggested that citokyne-induced killer (CIK) cells are able to kill both differentiated melanoma cells and putative melanoma CSCs (identified through a gene-transfer strategy involving the transduction with a lentiviral vector encoding the eGFP under expression control of the Oct4 promoter) [273]. Importantly, in these studies the cytolytic activity of CIK cells was tested in vitro and in vivo using CIK cells obtained from the blood mononuclear cells of metastatic melanoma patients and tested for their cytolytic activity against autologous tumor cells. In the in vivo studies, the CIK cells delayed tumor growth, increased necrotic areas, and promoted the lymphocyte infiltration of tumor sites [273]. 

In addition to the above-mentioned effects, a recent study provided evidence that TNF-α exerts an effect promoting the proliferation of melanoma CSCs. Thus, Ostyn and coworkers using an inducible H2B-GFP tracing system have shown that TNF-α increases the sub-population of quiescent or slow-cycling melanoma stem-like cells at the level of melanospheres [274]. This increase was associated with increased stem capacities of melanoma cells; i.e., with increased self-renewal and melanosphere-forming ability in vitro and with increased tumor-forming capacity in vivo [263]. Though serial passages, TNF-induced melanoma cells were shown to be capable of extensive self-renewal [274].

## 6. Circulating Tumor Cells and Circulating Tumor DNA in Melanoma

Recent studies carried out in many solid tumors have shown the existence of circulating tumor cells (CTCs). These cells are the tumor seeds responsible for the hematogenous dissemination of solid tumors, including melanomas, and derive from both primary and metastatic tumors. These cells can be identified in the circulating blood of many melanoma patients, and their characterization at the cellular and molecular levels contribute to the understanding of the mechanisms involved in tumor metastasis [275]. CTCs represent cancer biomarkers, and can be used to investigate the response to treatment at sequential times, without the need to use an invasive methodology [275]. Various studies have investigated the potential significance of CTCs as biomarkers in melanoma patients, indicating that their number may be predictive of clinical outcome and investigation of the treatment response [276].

However, the detection and characterization of CTCs is a technically challenging problem for two main reasons: the rarity of these cells in circulating blood, and the heterogeneity of CTCs at phenotypic level [275,276]. Given the paucity of CTCs in melanoma patients, their isolation through conventional methods based on the use of antibodies to epithelial membrane markers (e.g., EpCAM) are not suitable [275]. Thus, the best technical option to isolate melanoma CTCs is based on labeling-independent methods, such as microfluidic devices, utilizing differential cell size, density, and rigidity to separate CTCs from blood cell elements [277]. In melanoma, microfluidic devices such as CTC-Chip, Cluster-Chip, or herringbone CTC-Chip have been used to isolate single CTCs or clusters of CTCs from metastatic melanoma patients [276,277,278]. CTC numbers in *BRAF*-mutated melanomas decreased after treatment in patients responding to treatment [276]. Recently, using a slanted spiral microfluidic device, Aya-Bouilla and coworkers reported the isolation and characterization of CTCs from melanoma patients, obtaining a good enrichment and yield [279]. The analysis of the few isolated CTCs showed that these cells are heterogeneous and co-express stem-like markers, such as PAX3 and ABCB5 [279]. Given these consistent limitations, some authors have tried to develop xenografts from enriched populations of CTCs. Thus, Girotti and coworkers reported the attempt to grow melanomas from enriched preparations of CTCs in immunodeficient mice: out of 21 cases, they were able to grow melanoma cells in 6; all 6 cases corresponded to patients with a high tumor burden [280,281]. However, in most melanoma patients, the number of CTCs isolated is extremely low and the impact of the study of CTCs in melanoma thus remains limited. 

Circulating tumor DNA (ctDNA) has emerged in recent years as a promising blood-based biomarker for the monitoring of the disease status of patients with advanced cancers. Particularly, in melanoma, ctDNA has been shown to have a potential value as an alternative tumor source for the detection of genetic alterations and for the assessment of the response to therapy [282]. In some studies, the procedure of isolating ctDNA was called a “liquid biopsy”, to distinguish it from the traditional “solid biopsy” [282]. In this context, particularly interesting was the study carried out by Santiago-Walker and coworkers and based on the analysis of the use of ctDNA analysis in four clinical studies involving the treatment of *BRAF*-mutated melanomas with BRAF/MEK inhibitors [283]. BRAF mutations were detected in ctDNA isolated from the patient’s plasma in 71% and 81% of patients, respectively, with *BRAF^V600E^* and *BRAF^V600K^*-positive tumors [283]; patients with BRAF-positive tumors, but negative for BRAF-mutant ctDNA at diagnosis, had longer progression-free survival (PFS) and overall survival (OS) compared to patients for which BRAF mutations were detectable in the blood [283]. ctDNA analysis of BRAF mutations may be considered as a predictor of PFS in these patients [283].

Other studies have evaluated the feasibility of monitoring advanced melanoma patients using ctDNA, using an eight-gene screening; an 82% concordance was observed between ctDNA and tumor tissue analysis [284]. Interestingly, Girotti and coworkers have used ctDNA as a methodological strategy to monitor the response to therapy and the possible mechanism of resistance to treatment with MAPK/MEK inhibitors or with immune check point inhibitors [280]. Interestingly, the use of molecular analyses on ctDNA allowed a disease relapse to be monitored before routine LDH evaluation and to explore at the time of relapse the possible mechanisms of resistance by whole exome sequencing on ctDNA [280]. The same authors have shown that in a patient with *Kit*-mutant vaginal mucosal melanoma, it was possible to monitor tumor evolution during treatment by whole-exome sequencing carried out on ctDNA [285].

In conclusion, the pre-treatment detection of *BRAF^V600^*-mutant ctDNA is a prognostic factor for patients undergoing BRAF/MEK inhibitors treatment, and the monitoring of ctDNA of known driven mutations can be used for treatment monitoring and detection of acquired resistance. However, additional studies are required to assess the contribution of CT analyses, particularly in view of the consistent methodological heterogeneity existing in the various studies evaluating ctDNA in melanoma patients.

## 7. Development of New Melanoma Treatments

During the last decade, new anti-melanoma treatments have been developed, mainly based on targeted therapy and immunotherapy. The development of these new treatments was triggered by the progresses in the basic research of the genomics of melanoma and in the understanding of the mechanisms underlying the anti-cancer immune response. As mentioned in the section on molecular abnormalities, *BRAF*-mutant melanomas are amenable to new therapeutic regimens based on the combined administration of a BRAF inhibitor with an MEK inhibitor, showing a high rate of responder patients, but resistance—although delayed compared to patients treated with a BRAF inhibitor alone—remains inevitable. Relapse is attributed to the reactivation or over-activation of various signaling pathways, sustaining tumor survival.

In addition, immunotherapy is another treatment strategy that has recently achieved important improvements. Thus, several new agents have been introduced in recent years for the treatment of melanoma, including immunotherapeutic antibodies directed at cytotoxic T-lymphocyte associated antigen (CTLA-4) and programmed cell-death protein 1 (PD-1) or programmed cell-death ligand 1 (PD-L1) and small-molecule inhibitor of BRAF and MEK. For the majority of these agents, in phase III clinical trials evidence was obtained about an improvement of overall survival, compared to that obtained with standard therapies (reviewed in [286]). Actually, it is possible to obtain in a minority of metastatic patients, durable responses, and effective tumor control and palliation for the majority of these patients [286]. The development of immunotherapy studies has led to the registration of two therapeutic agents: ipilimumab, targeting the CTLA-4 (cytotoxic T lymphocyte-associated antigen-4), and nivolumab and pembrolizumab, both targeting the PD-1 (programmed cell death) receptor, both agents resulting in upregulation of host immune response against melanomas through a block of the inhibitory effect of T-lymphocyte regulators.

Distinct cellular mechanisms underlie anti-CTLA-4 and anti-PD-1 checkpoint blockade. Checkpoint blockade targets only specific subsets of tumor infiltrating T cell populations: anti-PD-1 predominantly induces the expansion of specific tumor-infiltrating exhausted-like CD8T cell subsets; in contrast, anti-CTLA-4 induces the expansion of an ICOS^+^Th-1like CD4 effector population, in addition to stimulate the expansion of specific tumor-infiltrating exhausted-like CD8^+^ lymphocytes [287]. Furthermore, anti-PD-1 therapy leads to a dynamic expansion of proliferating PD-1^+^ CD8 T cells in the peripheral blood of melanoma patients [288].

In phase III clinical trials, both these agents have been shown to produce improved survival. Treatment with immune check inhibitors in monotherapy (administered as monoclonal antibodies against anti-CTLA-4 or anti-PD-1) is associated with response rates ranging from 8% to 44%, and some of the responding patients displayed durable responses (i.e., >2 years). However, most patients do not respond to these therapeutic regimens as monotherapy, and some patients develop toxicity—particularly when these regimens are combined.

Particularly, ipilimumab—an anti-CTLA-4 mAb—has been shown to improve OS in two phase III studies, this improvement being significant but small. Despite these only marginal improvements in OS, the inspection of individual patients showed that the improvements in median OS are due to the presence of a population of about 20% of responding patients, surviving for years to the treatment [289]. More recently, Eggermont et al. reported the results of an EORTC study showing in a group of stage III melanoma patients who had undergone complete resection of the tumors, an advantage of overall survival in the ipilimumab arm (65.4% OS at 5.3 years of follow-up), compared to the placebo arm (54.4% OS). Thus, in these patients ipilimumab leads to an 11% improvement of OS, compared to placebo [290]. The analysis of the quality of life in these two groups of patients was comparable, although ipilimumab administration led to an increase of toxicity-related events [291]. However, the meta-analysis of all phase II/III studies carried out using ipilimumab versus placebo showed that this drug was responsible for about 1–5 of treatment-related deaths [292]. The response rates and the overall survival to treatment with ipilimumab were much lower for stage II and IV melanoma patients with unresectable tumors: median OS was 15.7 months for patients treated with ipilimumab at 10 mg/kg and of 12.5 months for patients treated at 3 mg/kg [293]. Among patients undergoing resection of stage III or IV melanoma, immunotherapy based on nivolumab administration resulted in a higher level of recurrence-free survival and a lower rate of grade 3 or 4 toxic events than immunotherapy based on ipilimumab administration [294]. 

Other studies have used anti-PD-1 blocking mAbs. The inhibitory mechanisms of immune response are triggered by melanoma cells, induced to express PD-L1 by IFN-γ released by peritumoral activated T lymphocytes or by oncogenic signaling pathways such as MYC, and through this mechanism conveying inhibitory signals to cytotoxic T lymphocytes expressing PD-1 on their surface. In phase III studies, nivolumab—a blocking mAb anti-PD-1—was shown to be superior to chemotherapy based on dacarbazine and ipilimumab in patients with advanced melanoma [295]. The same applies to pembrolizumab, another anti-PD-1 antibody [296]. A meta-analysis of all published data involving the PD-1 antibody monotherapy of 2828 adult patients provided evidence that this treatment compared to standard treatments resulted in an improvement of the six-month progression-free survival (PFS) and of the overall response rate [296]. Patients treated with nivolumab reported fewer treatment-related adverse events than those treated with other agents [297]. However, a recent phase III randomized trial failed to show an increased survival for patients treated with nivolumab (16 months), compared to the survival observed in patients treated with investigator’s choice chemotherapy (14 months) [298]. Only the overall response rate (27% vs. 10%) and median duration of response (32 months vs. 13 months) were higher for nivolumab vs. standard chemotherapy [298].

Preclinical studies have supported the rationale of combining anti-CTLA-4 and anti-PD-1 treatment, based on their synergistic capacity of stimulating anti-melanoma effects. Initial clinical trials have supported the anti-melanoma efficacy of the drug combination involving anti-PD-1 and anti-CTLA-4 mAbs. Subsequently, phase II and phase III studies have supported this conclusion. Thus, a phase II randomized clinical trial (CheckMate 069) comparing response to nivolumab + ipilimumab to ipilimumab + placebo showed 22% complete response in the former and 0% in the latter group [299]. At 1 and 2 years, the OS of the BRAF-non-mutated melanomas treated with the combination was 79% and 69%, respectively [299]. Another phase III study (CheckMate 067) compared three arms of treatment nivolumab+ipilimumab, nivolumab alone, and ipilimumab alone [300]. The combination arm showed an improved response rate and PFS rate compared to the two other arms [300]. At 18-month follow-up, the PFS was 46% for the combination arm, 39% for nivolumab, and 14% for ipilimumab arms [300]. At a follow-up of 36 months, the overall survival rate was 58% in the nivolumab+ipilimumab group and 52% in the nivolumab group, as compared with 34% in the ipilimumab group [301]. Treatment-related events of grade 3 or 4 occurred in 59% of patients in the nivolumab+ipilimumab group, in 21% of those in the nivolumab group, and in 28% of those in the ipilimumab group [301]. The important conclusion of this study was that in advanced melanoma patients, significantly longer survival occurred with combination therapy or with nivolumab than with ipilimumab alone [301]. In this study, the response of melanoma patients to therapy was analyzed in the two cohorts of *BRAF*-mutant and *BRAF*-WT tumors, providing evidence that after 36 weeks of treatment the overall survival was better among BRAF-mutant (68% of surviving patients), compared to BRAF-WT patients (53% of surviving patients) [301]. It is difficult to compare this rate of response with the rate of response observed for BRAF-mutant patients treated with BRAF and MEK inhibitors, but this result certainly raises the problem of the best therapy for *BRAF*-mutant melanomas, either BRAF/MEK inhibitors or anti-PD-1/anti-CTLA-4 immunotherapy. It is very interesting to point out that patient subgroup analysis showed that: (a) in the groups of patients showing at least 5% of PD-L1-positive tumor cells the PFS was similar in the two nivolumab-containing arms; (b) in the PD-L1-negative tumor cells the PFS was significantly higher in the combination arm, compared to the nivolumab-alone arm [302,303]. Toxicities were higher in the combination arm, compared to nivolumab alone. Given these results, combined CTLA-4 and PD-1 was approved by FDA for the treatment of *BRAF*-WT melanomas. 

A great problem of melanoma immunotherapy with immunocheck inhibitors is related to the considerable cost of these therapies [304]. Thus, it is particularly important to reduce the cost of these melanoma anti-therapies. A possible strategy to reduce their cost would consist of stopping these therapies after few cycles of treatment, but this point was not evaluated in specific clinical trials. However, a recent study provided some interesting indications deriving from the analysis of melanoma patients who stopped nivolumab+ipilimumab treatment due to adverse events [305]. In fact, about 40% of patients with advanced melanoma who received nivolumab+ipilimumab in clinical trials had to discontinue treatment (the majority during the induction phase) because of adverse events; a retrospective analysis of efficacy in these patients showed that the objective response rate was about 50% for patients who did not have to discontinue treatment and 58% for patients who discontinued because of adverse events [305]. These observations suggest that, even after discontinuation, many melanoma patients may continue to derive benefit from combination immunotherapy. 

The complexity of the mechanisms underlying a response to the immunotherapeutic treatment of melanomas implies the need for the identification of biomarkers predicting the response to these treatments. Thus, genomic and RNA-based studies have provided evidence that the tumor mutational load and neoantigen signature and the cytolytic activity are parameters associated with response to immunotherapy and survival after treatment [306,307,308]. Tumor immunohistochemistry indicates that the level of PD-1^+^ T-lymphocytes (CD4^+^ and CD8^+^), as well as the level of PD-L1 expression on tumor cells predict the response to therapy [309,310]. PD-L1 expression on tumor cells and on T lymphocytes is regulated by the membrane proteins CMTM4 and CMTM6: CMTM6 associates on the cell surface with the PD-L1 protein, reduces its ubiquitination, and increases PD-L1 protein half-life [311]. Importantly, CMTM6 depletion selectively decreases PD-L1 levels, without affecting MHC class I, and alleviates the suppression of tumor-specific T cell activity in vitro and in vivo [312]. However, these markers are not always predictive of melanoma response to immune checkpoint blockade, and more accurate biomarkers are needed. 

Other studies have tried to better define the differences in the anti-tumor immune response elicited by different forms of immune checkpoint blockade. Thus, some studies have explored the transcriptome profile and the signaling pathways activated in immune cells (T lymphocytes and monocytes) by PD-1 and CTLA-4 blockade, respectively [313,314]. Thus, it was shown that PD-1 blockade determines changes in genes involved in natural killer cell function and lymphocyte cytolytic activity, while CTLA-4 blockade induces a proliferative signature in memory T lymphocytes [314]. Furthermore, it was shown that PD-1 blockade determines at the level of CD8^+^ T lymphocytes an increased IL-2 signaling, response to type I interferon, and metabolic changes, while CTLA-4 blockade induced in these cells an increased NFAT-JAK-STAT signaling, cell proliferation, and activation of effector pathways [314].

Features of the tumor microenvironment are also associated with response to checkpoint inhibitor therapy. In this context, the studies carried out in melanoma patients undergoing anti-PD-1 therapy have shown three major findings: an expression of PD-L1 in the TME associated with clinical response to anti PD-1/PD-L1 therapies; baseline levels of tumor-infiltrating CD8^+^ T lymphocytes correlate with the probability of response and tend to increase in responding, but not progressing tumors; the location of CD8^+^ T lymphocytes at the invasive margin of tumors is indicative of an effective immune response. Riza and coworkers have recently investigated tumor microenvironment evolution during treatment with an anti-PD-1 drug [315]. Tumors were analyzed by whole-exome, transcriptome, and T cell receptor sequencing. This study provided several fundamental conclusions: (i) increased tumor load is associated with response to immune checkpoint therapy; (ii) after 4 weeks of therapy, a marked decrease in detectable mutations was observed in patients with complete/partial responses, but only a moderate decrease in patients with stable disease; (iii) clonality analysis showed that anti-PD-1 therapy affects the whole clonal populations in patients with complete/partial responses, while the treatment affected only some subclones in patients exhibiting stable disease; (iv) genomic evidence of effective immune elimination of tumor cells containing non-synonymous mutations and neoantigens was obtained in responding patients; moreover, T cell clones expanded during treatment in these patients in proportion to the number of neoantigenic mutations that became undetectable on therapy; (v) analysis of the transcriptome of pre-therapy tumor samples showed the existence of a subset of upregulated immune-related genes in patients responding to immunotherapy; (vi) analysis of gene expression during treatment provided evidence about the marked upregulation of a multitude of immune pathways, more pronounced among responding patients [315].

On the other hand, other studies have attempted to define the molecular and cellular mechanisms underlying resistance to immune checkpoint blockade. In this context, there is growing evidence that various mechanisms could contribute to these resistance phenomena, including the occurrence of somatic mutations at the level of genes involved in antigen processing and presentation, upregulation of genes involved in angiogenesis, cell adhesion, and extracellular matrix remodeling [316,317]. Finally, other studies based on animal models and on melanoma samples of treated patients support an inhibitory role to immunotherapy played by activation of the WNT/β-catenin signaling pathway in tumor cells [317]. Chen and coworkers have performed a longitudinal analysis of tumor biopsies of melanoma patients first undergoing therapy with CTLA-4 blockade and then treated with PD-1 blockade if they did not respond or progressed on therapy [318]. Thirteen percent of the treated patients with CTLA-4 blockade achieved clinical benefit, and 87% did not [318]. At the pretreatment level, there were no differences in any tested parameter between responders and nonresponders to CTLA-4 blockade treatment [318]. Only the analysis of early on-treatment biopsies displayed a parameter (CD8^+^ T lymphocytes) predictive of response to treatment [319]. Early on-treatment biopsies, analyzed by immune profiling based on the study of the expression of T-cell markers CD3, CD4, CD8, PD-1, and PD-L1 were highly predictive of response to PD-1 blockade [318]. Finally, gene expression profiling carried out on early on-treatment tumor samples of patients undergoing anti-PD-1 therapy showed the predictive value of an expression signature characterized by the upmodulation of genes encoding cytolytic markers, HLA molecules, IFNγ pathway effectors, chemokines, and some adhesion molecules [318].

Recent studies suggest that IFNγ is a major determinant in the response or resistance to immunotherapy. Melanoma cells from patients responding to immunotherapy are sensitive to the anti-proliferative and pro-apoptotic effects of IFNγ, and the continuous exposure to the cytokine could select for the outgrowth of IFNγ-resistant subclones [319]. IFNγ-resistant clones with inactivating *JAK1*/*JAK2* mutations frequently evolve in melanoma patients receiving different types of immunotherapy [319]. Another recent study provided evidence that a gene expression signature including IFNγ-responsive genes related to antigen presentation, chemokine expression, cytotoxic activity, and adaptive immune resistance predicts the response of melanoma and other cancer patients to immunotherapy [320]. Another study showed that the expression of IFN-γ signaling genes is associated with response to immunotherapy [321]. Furthermore, the functional essentiality of antigen presentation for immunotherapy was shown. The expression of these genes correlates with cytolytic activity in patient tumors [321]. Interestingly, some patients displaying resistance to immunotherapy with immune check inhibitors exhibited loss-of-function mutations of the APLNR gene, encoding the apelin receptor and involved in the modulation of IFN-γ responses [321]. Zaretski and coworkers have analyzed the genomic landscape of melanoma patients relapsing after an initial response to PD-1 blockade therapy [322]. It was estimated that about 25% of patients responding to anti-PD-1 immune check inhibitors had disease progression at a follow-up of about 20 months. Fifty percent of these relapsing patients displayed resistance-associated loss-of-function mutations in one allele encoding Janus Kinase 1 (*JAK1*) or Janus Kinase 2 (*JAK2*), with concurrent deletion of the wild-type allele [322]. Twenty-five percent of these relapsing patients displayed a truncating mutation in the gene encoding β2-microglobulin, leading to a loss of membrane expression of HLA class I [322]. These observations support the view that acquired resistance to PD-1 blockade immunotherapy was associated with defects in the pathways involved in IFN receptor signaling and in antigen presentation. 

The efficacy of immunotherapies in humans is related to the capacity of addressing a T cell response against cancer neoantigens, a class of HLA-bound peptides arising from tumor-specific mutations. Many of these neoantigens are highly immunogenic because they are absent in normal tissues [323]. The discovery of these neoantigens is only recent, being linked to the development of massively parallel sequencing for detecting coding mutations occurring in tumors. A strategy of cancer immunotherapy consists of the production of vaccines containing tumor neoantigens: vaccination with tumor neoantigens should determine an expansion of pre-existing neoantigen-specific T cell populations and induce the production of new specific anti-tumor T cell clones [324]. Using this innovative strategy, a recent study evaluated the safety and efficacy of an immunogenic personal neoantigen vaccine, targeting up to 20 predicted personal tumor neoantigens, in six melanoma patients [324]. Of these six patients, four had no recurrence at 2 years after vaccination, while the two remaining patients with recurrent disease were subsequently treated with anti-PD-1 therapy and displayed complete tumor regression [324]. These observations strongly support further development of this vaccination approach, alone or in combination with checkpoint blockade.

A systematic understanding of anti-tumor immunity at a systemic level is required to achieve significant progress in developing rational immunotherapeutic strategies. The consistent variability in clinical responses to immunotherapy in melanoma patients strongly suggests that the development of systemic immunological responses against cancer cells are complex and regulated by a multifactorial system. In this context, the analysis of various experimental models and of the clinical data observed in melanoma patients treated with immune check inhibitors indicate that the development of a systemic anti-tumor response is required for effective cancer immunotherapy, involving both a CD8- and CD4-mediated anti-tumor response [325]. 

As repeatedly reported above, 15–20% of cutaneous melanomas display *NRAS* mutations; this melanoma subset is reported to be more aggressive than non-*RAS*-mutated melanomas. The best treatment for NRAS-mutated melanomas remains undetermined, and no specific target therapy is available for these tumors. Current therapies for *NRAS*-mutant melanomas remain very limited. Some studies have evaluated a possible therapeutic effect of MEK inhibitors in this melanoma subtype, showing a limited clinical benefit. In this context, a recent study provided evidence of some clinical activity of the MEK1/2 inhibitor binimetinib (MEK162): in a phase 3 study (NEMO trial), binimetinib improved PFS in comparison with dacarbazine (2.8 months vs. 1.5 months) [326]. According to this finding, binimetinib may represent a new treatment option for *NRAS*-mutant patients who have failed after initial immunotherapy. Preclinical studies support the existence of positive mechanistic interaction between MEK inhibitors with cyclin-dependent kinases 4 and 6 (CDK 4/6) inhibitors, MDM2 antagonists, or PI3K/AKT pathway inhibitors, triggering the development of ongoing phase I–II clinical studies. Very few studies have specifically addressed the problem of the response of *NRAS*-mutated melanomas to immunotherapy. Johnson et al. reported a higher rate of clinical benefit (objective responses + stable disease) of NRAS-mutated melanomas to immunotherapies (IL-2, ipilimumab, and anti-PD-1/PD-L1) compared to BRAF-mutated or no *BRAF/NRAS*-mutated melanomas [327]. A recent study presented at the last ASCO Meeting reported the study of the response of a cohort of 224 metastatic melanoma patients to checkpoint inhibitors, and showed an objective response rate of 15% and PFS of 4.5 months among the 180 patients receiving ipilimumab, and a response rate of 34% among the 98 patients undergoing PD-1 monotherapy [328]. Overall survival for all patients was 29 months [328]. According to these data, it was concluded that the efficacy data of ipilimumab and anti-PD-1 therapy in *NRAS*-mutated melanoma patients were comparable to the known responses observed in *NRAS* wildtype patients [328].

As mentioned above, the treatment of BRAF-mutated melanomas was largely based on the use of BRAF inhibitors. Two second-generation RAF inhibitors—vemurafenib and dabrafenib—showed remarkable clinical activity in patients with *BRAF^V600E/K^* melanoma, and were approved for the treatment of this melanoma subtype. Although these drugs significantly prolonged patient survival compared to standard treatment, their effect is not curative and is limited by the development of drug resistance and consequent relapse. Combined treatment of RAF inhibitors with MEK inhibitors was used to bypass resistance and to improve anti-tumor response. Both combinations (vemurafenib and cobimetinib and dabrafenib and trametinib) improved clinical efficacy compared to RAF inhibitor monotherapy. Thus, the updated results of a phase 3 study in patients with advanced BRAF^V600^-mutation-positive melanoma (stage IIIC and IV) showed a median overall survival 22.3 months for cobimetinib and vemurafenib groups versus 17.4 months for Vemurafenib and placebo [329]. Two phase 3 trials (COMBI-d and COMBI-v) have shown that combined treatment with the BRAF inhibitor dabrafenib and the MEK inhibitor trametinib improved overall survival in patients with unresectable or metastatic melanoma with BRAF^V600E/K^ mutations. Furthermore, a recent study provided evidence that adjuvant dabrafenib plus trametinib in stage III resected *BRAF*-mutated melanoma significantly improved PFS and OS compared to placebo [330]. The 3-year overall survival was 86% in the combination therapy group and 77% in the placebo group [330]. Interestingly, the analysis of long-term outcomes in patients with *BRAF^V600^*-mutant metastatic melanoma who received dabrafenib plus trametinib showed an overall survival of 28% at 5 years [331]. The overall survival was increased among patients who received the combination therapy with normal baseline lactate dehydrogenase levels, particularly when associated with fewer than three organs sites with metastasis [331]. In *BRAF*-mutated melanomas, the dabrafenib and trametinib association also showed clinical activity against brain metastases; however, the media duration of the response was relatively short [332].

Schreuer et al. performed an interesting clinical study attempting to demonstrate a possible sensitivity of BRAF^V600^-mutant melanoma patients, who had previously progressed on BRAF inhibitors and were off-treatment for several weeks, to a new re-challenge based on dabrafenib + tramatenib administration [333]. A partial response was observed in 32% and a stable disease in 40% of the treated patients [333]. Thus, drug rechallenging could represent a potential new treatment option for these patients [333]. This finding is particularly interesting and indicates the importance of cancer drug addiction. In fact, as cancer cells become resistant to drugs that target cancer-specific signaling pathways, an intriguing phenomenon called cancer addiction sometimes develops [203]. This phenomenon was initially reported for *BRAF^V600^*-mutated melanomas treated with BRAF inhibitors [203]. Interestingly, it was shown that a discontinuous dosing strategy, exploiting the fitness disadvantages displayed by drug-resistant cells when they are not exposed to the inhibitory drugs, forestalls the onset of drug resistance [203]. Recently, Kogass et al. have used an elegant experimental approach to identify the genes encoding the proteins ERK2 and JUNB as the main mediators of the drug-addiction process: genetic depletion of *ERK2* (also known as MAPK1), JUNB blocked the cell death of drug-addicted cells [334]. This drug addiction was highly specific in that ERK1 depletion, despite ERK2 depletion, was unable to block drug addiction [334]. When drug-addicted melanoma cells are deprived of the inhibitory drug they undergo a death process, characterized at the gene expression level by a switch of expression profile from proliferation to increased expression of genes associated with metastasis [334]. Interestingly, drug-addicted cells are particularly sensitive to the cell killing by the drug dacarbazine [334]. The response of MAPK inhibitor-resistant cells to drug withdrawal is variable, in that some melanoma cells respond with a lowering of cell cycling, while other cells undergo an apoptotic process [335]. Importantly, the phenomenon of MAPK inhibitory addiction is not exclusive to BRAF^V600^ mutant melanoma cells, but is a hallmark of MAPK inhibitors-resistant melanoma cells, including both BRAF^V600^-mutant and NRAS-mutant cells [335]. Pharmacological impairment of DNA damage repair markedly augmented the MAPK inhibitor addiction by switching a cell-cycle deceleration to a caspase-induced cell death [335]. Interestingly, in MEK inhibitor-resistant NRAS-mutant melanoma cells, treatment with a type I RAF inhibitor such as vemurafenib intensified the p-ERB rebound elicited by MEK inhibitor withdrawal and promoted cell death [335].

The development of drug resistance represents the main limitation of the therapy with RAF inhibitors. As mentioned above, the identified resistance mechanisms include gain-of-function mutations at the level of *NRAS, MAP2K1*, and *PIK3CA*; upregulation of *PDGFRβ, EGFR, ERBB3*, and *IGFR1*; amplification or splice variant expression of *BRAF*. The majority of these mechanisms determine MAPK activation through a mechanism independent of BRAF-mutant, and this explains the clinical studies based on the combination of a BRAF inhibitor with MEK1/2 or ERK1/2 inhibitors. In addition to these mechanisms, recent studies suggest an important role of the transcription factor MITF in regulating the response of BRAF-mutated melanoma to BRAF inhibitors. Particularly, MITF can induce resistance to MAPK-pathway inhibitors through various mechanisms, including enhanced survival signaling and alterations of metabolism [336]. Enhanced MITF expression is linked to innate resistance, and MITF focal amplifications are linked to the BRAF mutant subtype. Importantly, MITF expression is upregulated by BRAF-induced MAPK signaling. The upregulation of the melanoma survival oncogene MITF induces drug tolerance; this conclusion is supported by the observation that the protease inhibitor acts as a potent suppressor of PAX3 and MITF expression and strongly sensitizes BRAF and NRAS mutant melanoma cells to MAPK-pathway inhibitors [336]. Furthermore, MITF levels within a melanoma tumor are heterogeneous at the single cell level and this heterogeneity affects the response of the tumor to BRAF inhibition; in fact, this MITF expression heterogeneity creates a dynamic cell population in which cells with different MITF levels influence one another’s drug response profiles via paracrine signals [337]. This paracrine protection to MAPK inhibition is mediated by increased expression of endothelin-converting enzyme-1, which produces biologically active endothelin-1, which in turn stimulates the proliferation of cells expressing the endothelin receptor A (EDNRA) [337]. EDNRA inhibitors synergize with BRAF inhibitors in interfering with the growth of melanoma xenografts [337]. Interestingly, Eskiocak et al. have identified two subtypes of BRAF-mutated melanomas segregated according to SOX10 expression [338]. The SOX10-dependent BRAF^V600^-mutated melanoma respond to clinical BRAF and MEK inhibitors; the SOX10-independent subtype is resistant to targeted therapy, but is sensitive to the inhibition of TBK1/IKKε kinase activity [338]. This second cohort includes BRAF mutant and BRAF wild-type tumors and corresponds to a gene expression signature reminiscent of host defense pathway activation and TGFβ-induced mesenchymal status [338].

Some recent studies suggest that metastasizing melanoma cells are sensitive to oxidative stress. This study was originated from the observation that melanomas grow subcutaneously, but have only humoral cells able to grow in immunodeficient animals after intravenous or intrasplenic transplantation, and this peculiar property seems to be related to the incapacity of most of melanoma cells to survive to the oxidative stress that these cells experienced in the blood and visceral organs, but not observed in the subcutaneous tissue [339]. Melanoma cells able to metastasize underwent reversible metabolic changes enabling these cells to survive to an oxidative stress, particularly characterized by increased dependence on NADPH-generating enzymes in the folate pathway [339]. In line with these observations, anti-oxidants promoted metastasis formation of melanoma cells when injected into immunodeficient mice, while folate inhibition by methotrexate inhibited distant metastasis formation, but did not affect the growth of subcutaneous melanomas [339].

## Figures and Tables

**Table 1:** Main properties of different types of melanocytic nevi.

Nevus Type	Main Biological Properties	Molecular Analysis	Risk of Transformation to Melanoma	Cellular Origin
**Congenital Melanocytic Nevus (CMN)**	CMN is a benign clonal melanocytic proliferation that develops in utero. CMNs are classified according to their size, as: Small (<1.5 cm)/Medium (<20 cm) Large (20–40 cm)/Giant (>40 cm) Small/Medium: 1% of birth Large: 1/20,000/50,000 births Giant: 1/200,000–500,000 births	Low mutational burden. Recurrent mutations: NRAS Q61; BRAF^V600E^ Small/Medium: 30% BRAF; 70% NRAS Large: 5–10% BRAF; 80–90% NRAS Giant: 5% BRAF; 90–95% NRAS.	The risk of melanoma transformation is related to the nevus size. Small/Medium: Low Large/Giant: 4–10%	CMNs may derive from mutations at the level of neural crest cells. Smaller lesions arise from the descendants of a single somatically mutated cell, where the driver mutation occurred later than in the larger lesions. According to mouse models, if the BRAF mutation occurred in melanoblasts or earlier progenitors, it would be incompatible with post-natal life; in contrast, NRAS mutations occurring in neural crest cells are compatible with life.
**Acquired Melanocytic Nevus (AMN)**	AMNs are a benign, clonal melanocytic proliferation that develops after birth. AMNs are classified according to their dermoscopic patterns: Globular: predominates in youth and has a lifelong persistence; have a dermal growth pattern. Reticular: predominates in adulthood; has an epidermal growth pattern.	Low mutation burden. 100% AMNs have mutations of MAPK pathway. Recurrent mutations: NRAS Q61; BRAF^V600E^ Globular: 92% BRAF; 8–10% NRAS Reticular: 65–70% BRAF; 30–35% NRAS Whole: 85% BRAF; 15% NRAS.	The risk of melanoma transformation is low; 20–30% of cutaneous melanomas develop from pre-existing AMNs.	AMNs derive from single melanocyte precursors, whose limited, benign, clonal expansion is dictated by BRAF or NRAS mutations.
**Dysplastic Melanocytic Nevus (DMN)**	DMNs are also known as Clark nevi or moles. DNSs display variability in size, border, and colors. In Europe, from 2% (Germany) to 8% (Sweden) of the population have DNSs.	Low mutational burden. Increased microsatellite instability and ROS production.BRAF^V600E^: 60–70%. No mutations of CDKN2A, TP53, NF1, RAC1 and PTEN.	DMNs have a 10-fold increased risk of developing cutaneous melanoma, compared to AMNs. Their role as transition lesions from AMNs to cutaneous melanomas is not completely demonstrated.	DMNs display the same cellular origin as AMNs.
**Blue Nevi (BN)**	The common BN is a flat to slightly elevated smooth-surfaced macule, papule, or plaque that is bluish black in color. The cellular BNs are larger lesions (1–3 cm). They derive their name from the blue color observed when viewed clinically, an appearance attributed to the Tyndall effect.	BNs display fewer BRAF mutations compared to CMNs and AMNs, but show frequent somatic mutations in the heterotrimeric G protein α-subunit, GNAQ (about 80%). Other frequent mutations: KRAS (15–20%), GNA11 (3–5%), CYSLTR2 (3%). BN-like melanomas display more frequent BRAF mutations and BAP1, SF3B1, and EIF1AX alterations.	The risk of melanoma transformation is low.	BNs derive from the limited clonal expansion of melanocyte precursors acquiring a GNAQ or GNA11 mutation. All of these mutations occur at the level of Glutamine 209 and determine constitutive activation of MAPK pathway.
**Spitz Nevi (SN)**	Spitz tumors are a subgroup of melanocyte neoplasms, ranging from benign (Spitz nevi) to malignant (Spitz melanoma), with intermediate malignancy tumors (atypical Spitz tumors), characterized by large epithelioid or spindled melanocytes. Their incidence is low.	15–20% Spitz nevi harbor HRAS alterations (copy number gain or mutation); these nevi are characterized by desmoplasia, and are known as desmoplastic Spitz nevi. 55% of Spitz nevi harbor kinase fusions, mostly ROS1, ALK, and NTRK1 fusions; less frequent are BRAF and RET fusions; kinase fusions are mutually exclusive with HRAS alterations. 5% of atypical Spitz nevi display BAP1 allelic loss and BRAF mutations: these nevi are associated with a hereditary predisposition tumor syndrome or may be sporadic; these tumors have an epithelioid morphology and make part of atypical Spitz tumors.	The risk of melanoma transformation for Spitz nevi is very low. The risk of melanoma transformation for atypical Spitz tumors is low.	The cellular origin is similar to AMNs.

BAP1: BRCA1 Associated Protein 1; CYSLTR2: Cysteinyl Leukotrien Receptor 2; SF3B1: Splicing Factor 3b Subunit 1; EIF1AX: Eukaryotic Initiation Factor 1A, X-Chromosomal.

**Table 2:** Major molecular groups of melanomas defined according to the presence of a main driver mutation.

Molecular Subtype	Frequency	Co-Occurrence of Other Mutations or Copy Number Alterations (CNAs)	Activated Signaling Pathways
**BRAF** V600E in 75% V600K in 11% V600R in 2% K601 in 3% Exon 11 mutations in 6%	50–55%	V600 and K601 hot-spot mutations are mutually exclusive with *NRAS* mutations. *BRAF* non-hot-spot mutations (exon 11) co-occurred with *RAS* mutations. About 10% of cases display NF1 mutations. *CDK2NA* mutations in 15%. *TERT* promoter mutations in 75%. Various CNAs, particularly of *BRAF, MITF* and *TERT* genes.	MAPK→BRAF mut, MEK1 Cell cycle: CDKN2A mut/del/h-meth (60%) DNA damage: TP53 mut (10%) PI3K/AKT: PTEN mut/del (20%) Epigenetics: IDH1 mut, ARID2 mut (15%) Telomerase: Prom mut (75%)
**RAS** NRAS mutations (28 of melanomas): the most frequent mutations are Q61R, Q61K, Q61L, Q61H, G12R/D/A and G13R/D. HRAS mutations (1.5% of melanomas). KRAS mutations (1% of melanomas).	30–32%	RAS mutations are almost exclusive with BRAF mutations. A minority displayed *NF1* mutations. *TP53, CDKN2A, ARID2*, and *PPP6C* were detectable in a minority of cases. *TERT* promoter mutations in 72% of cases. Various CNAs, none frequent.	MAPK→RAS mut MEK1, ERK1/2 Cell cycle: CDKN2A mut/del/h-meth (70%); CCND1 ampl (10%) DNA damage: TP53 mut (20%) PI3K/AKT: AKT3 overexpression (40%) Epigenetics: IDH1 mut, ARID2 mut (15%) Telomerase: Prom mut (70%)
**NF1** Inactivating or damaging mutations, mostly nonsense, splice-site and frame-shift indels. NF1 mutations determine MAPK activation.	14–15%	This subtype had the highest mutation prevalence (39 mutations/Mb). Rarely display *RAS* mutations; about 15% display *BRAF* mutations. Mutations in melanoma-driver genes affecting cell proliferation and survival, such as TP53, *RAC1, PTEN, CDKN2A, MAP2K*, and *RB1*. 60% of *NF1* mutant (BRAF and RAS-WT) melanomas display mutations in RASopathy genes (*RASA2, SOS1, RAF1, SPRED1*). Frequent (83%) *TERT* promoter mutations.	MAPK→NF1 low function mut CRAF Cell cycle: CDKN2A mut/del/h-meth (70%); RB1 mut (10%) DNA damage: TP53 mut (30%) PI3K/AKT: AKT3 overexpression (30%) Epigenetics: IDH1 mut; EZH2 mut; ARID2 mut (30%) Telomerase: TERT Prom mut (83%)
**TRIPLE WILD-TYPE** A heterogeneous group characterized by a lack of hot-spot BRAF, RAS and NF1 mutations.	12–14%	Frequent are *Kit* mutations (12%) and *CTNNB1* (6%); *GNA11* (4%), *GNAQ* (2%) and *EZH2* mutations (2%) are also observed. *TERT* promoter mutations are less frequent (7%) than in other melanoma subtypes. CNAs are frequent, particularly involving *Kit, PDGFRA, CDK4, CCND1, MDM2,* and *TERT*.	MAPK→Kit mut/amp, KDR amp, PDGFRA amp, GNA11 mut KIT and BCL-2 overexpression Cell cycle: CDKN2A mut/del/h-meth (40%); CCDN1 amp (10%); CDK4 amp (15%) DNA damage: MDM2 amp PI3k/AKT: AKT3 overexpression (30%) Epigenetics: IDH1 mut Telomerase: TERT Promoter mut (10%), TERT amp (15%)

**Table 3:** Recurrent hot spot mutations in uveal melanoma.

Gene	Codon	Mutation	Frequency of Mutation
*GNA11*	Gln209	Gln209Leu	40–45
*GNAQ*	Gln209	Gln209Pro Gln209Leu	50–55
*SF3B1*	Arg625	Arg625His Arg625	15–18
*SF3B1*	Lys666	Lys666Thr	3–5
*EIF1AX*	Gly6	Gly6Asp Gly6Val	13–16
*PLCB4*	Asp630	Asp630Tyr Asp630Phe Asp630Asn	5
*CYSLTR2*	Leu126	Leu129Gln	4
*BAP1*	Different		35–40

PLCB4: Phospholipase C Beta 4.

## References

[1] Mitra D., Luo X., Morgan A., Wang J., Hoang M.P., Lo J., Guerrero C.R., Lennerz J.K., Mihm M.C., Wargo J.A. (2012). An ultraviolet-radiation-independent pathway to melanoma carcinogenesis in the red hair/fair skin background. Nature.

[2] Noonan F.P., Zaidi M.R., Wolnicka-Glubisz A., Anver M.R., Bahn J., Wielgus A., Cadet J., Douki T., Mouret S., Tucker M.A. (2012). Melanoma induction by ultraviolet A but not ultraviolet B radiation requires melanin pigment. Nat. Commun..

[3] Sommer L. (2011). Generation of melanocytes from neural crest cells. Pigment Cell Melanoma Res..

[4] Nishimura E.K., Suzuki M., Igras Y., Du J., Lonning S., Miyachi Y., Roes J., Beermann F., Fisher D.E. (2010). Key roles for transforming growth factor β in melanocyte stem cell maintenance. Cell Stem Cell.

[5] Hari L., Miescher I., Shakhova O., Suter U., Taketo M., Richardson W.B., Kessaris N., Sommer L. (2012). Temporal control of neural crest lineage generation by Wnt/β-catenin signaling. Development.

[6] Rabbani P., Takeo M., Chou W., Myung P., Bosenberg M., Chin L., Taketo M.M., Ito M. (2011). Coordinated activation of Wnt in epithelial and melanocyte stem cells initiates pigmented hair regeneration. Cell.

[7] Liao C.P., Booker R.C., Morrison S.J., Le L.Q. (2017). Identification of hair shaft progenitors that create a niche for hair pigmentation. Genes Dev..

[8] Nitzan E., Platzgraff E.R., Labosky P.A., Kalcheim C. (2013). Neural crest and Schwann cell progenitor-derived melanocytes are two spatially segregated populations similarly regulated by Foxd3. Proc. Natl. Acad. Sci. USA.

[9] Clark W.H., Elder D.E., Guerry M.N., Epstein M.N., Green M.H., Van Horm M. (1984). A study of tumor progression: The precursor lesions of superficial spreading and nodular melanomas. Hum. Pathol..

[10] Muller A.J., Mihme M.C. (2006). Melanoma. N. Engl. J. Med..

[11] Van den Hurk K., Niessen H., Veek J., van den Oord J.J., van Steensel M.A., Zur Hausen A., van Engeland M., Winnepenninckx V.J. (2012). Genetics and epigenetics of cutaneous malignant melanoma: A concert out of tune. Biochim. Biophys. Acta.

[12] Vidwans S.J., Flaherty K.T., Fisher D.E., Tenenbaum J.M., Travers M.D., Shrager J. (2011). A melanoma molecular disease model. PLoS ONE.

[13] Heidom S.J., Milagre C., Whittaker S., Nourry A., Niculescu-Duvas I., Dhomen N., Hussain J., Reis-Filho J.S., Springer C.J., Pritchard C. (2010). Kinase-dead BRAF and oncogenic RAS cooperate to drive tumor progression through CRAF. Cell.

[14] Damsky W.E., Curley D.P., Santhanakrisnan M., Rosenbaum L.E., Platt J.T., Rothberg B.E.G., Taketo M.M., Dankort D., Rimm D.L., McMahon M. (2011). β-catenin signaling controls metastasis in BRAF-activated PTEN-deficient melanomas. Cancer Cell.

[15] Sosman J.A., Kim K.B., Schuschter L., Gonzales R., Pavlick A.C., Weber J.S., McArthur G.A., Huston T.E., Moschos S.J., Flaherty K.T. (2012). Survival in BRAF V600-mutant advanced melanoma treated with vemurafenib. N. Engl. J. Med..

[16] Mc Clenahan P., Lin L.L., Tan J.M., Flewell-Smith R., Schaider H., Jagirdar K., Atkinson V., Lambie D., Prow T.W., Sturm R.A. (2014). BRAF^V600E^ mutation status of involuting and stable nevi in dabrafenib therapy with or without trametinib. JAMA Dermatol..

[17] Hatzivassiliou G., Song K., Yen I., Brandhuber B.J., Anderson D.J., Alvarado R., Ludlam M.J., Stokoe D., Gloor S.L., Vigers G. (2010). RAF inhibitors prime wild-type RAF to activate the MAPK pathway and enhance growth. Nature.

[18] Colombino M., Capone M., Lissia A., Cossu A., Rubino C., De Giorgi V., Massi D., Fonsatti E., Staibano S., Nappi O. (2012). BRAF/NRAS mutation frequencies among primary tumors and metastases in patients with melanoma. J. Clin. Oncol..

[19] Colombino M., Lissia A., Capone M., De Giorgi V., Massi D., Stanganelli I., Fonsatti E., Maio M., Botti G., Caracò C. (2013). Heterogeneous distribution of BRAF/NRAS mutations among Italian patients with advanced melanoma. J. Transl. Med..

[20] Liu J., Takata M., Munata H., Goto Y., Kido K., Ferrone S., Saida T. (2009). Polyclonality of BRAF mutations in acquired melanocytic nevi. J. Natl. Cancer Inst..

[21] Liu J., Goto Y., Munata H., Sakaizawa K., Uchiyama A., Saida T., Takata M. (2011). Polyclonality of BRAF mutations in primary melanoma and the selection of mutant alleles during progression. Br. J. Cancer.

[22] Heinzerling L., Baiter M., Kuhnapfel S., Schuler G., Keikavoussi P., Agaimy A., Kiesewetter F., Hatrmann A., Schneider-Stock R. (2013). Mutation landscape in melanoma patients: Clinical implications of heterogeneity of BRAF mutations. Br. J. Cancer.

[23] Menzies A.M., Lum T., Wilmott J.S., Hyman J., Kefford R.F., Thompson J.F., O’Toole S., Long G.V., Scolyer R.A. (2014). Intrapatient homogeneity of BRAFV600E expression in melanoma. Am. J. Surg. Pathol..

[24] Shen C.H., Kim S.H., Trousil S., Frederick D.T., Piris A., Yuan P., Cai L., Gu L., Li M., Lee J.H. (2016). Loss of cohesion compolex components STAG2 or STAG3 confers resistance to BRAF inhibitioin in melanoma. Nat. Med..

[25] Jalili A., Wagner C., Pashenkov M., Pathria G., Mertz K.D., Widlund H.R., Lupien M., Brunet J.P., Golub T.R., Stingl G. (2012). Dual suppression of the cyclin-dependent kinase inhibitors CDKN2C and CDKN1A in human melanoma. J. Natl. Cancer Inst..

[26] Falchook G.S., Lewis K.D., Infante J.R., Gordon M.S., Vogelzang N.J., DeMarini D.J., Sun P., Moy C., Szabo S.A., Roadcap L.T. (2012). Activity of the oral trametinib in patients with advanced melanoma: A phase 1 dose-escalation trial. Lancet Oncol..

[27] Wagle N., Van Allen E., Treacy D.J., Frederick T., Cooper Z.A., Taylor-Weiner A., Rosenberg M., Goetz E.M., Sullivan R.J., Farlow D.N. (2014). MAP Kinase pathway alterations in BRAF-mutant melanoma patients with acquired resistance to combined RFA/MEK inhibition. Cancer Discov..

[28] Flaherty K.T., Infante J.R., Dande A., Gonzalez R., Kefford R.F., Sosman J., Hamid O., Schuchter L., Cebon J., Ibrahim N. (2012). Combined BRAF and MEK inhibition in melanoma with BRAF V600 mutations. N. Engl. J. Med..

[29] Shi H., Hugo W., Kong X., Hong A., Koya R.C., Moriceau G., Chodon T., Guo R., Johnson D.B., Dahlman K.B. (2014). Acquired resistance and clonal evolution in melanoma during BRAF inhibitor therapy. Cancer Discov..

[30] Van Allen E.M., Wagel N., Sucker A., Treacy D.J., Johannessen C.M., Goetz E.M., Place C.S., Taylor-Weinr A., Whittaker S., Kryukov G.V. (2014). The genetic landscape of clinical resistance to RAF inhibition in metastatic melanoma. Cancer Discov..

[31] Raaijmakers M., Widmer D., Narechania A., Eichoff O., Freiberger S., Wenzina J., Cheng P., Mihic-Probst D., Desalle R., Dummer R. (2016). Co-existence of BRAF and NRAS driver mutations in the same melanoma cells results in heterogeneity of targeted therapy resistance. Oncotarget.

[32] Fallahi-Sichani M., Becker V., Izar B., Baker G., Lin J.R., Boswell S., Shah P., Rotem A., Garraway L., Sorger P.K. (2017). Adaptive resistance of melanoma cells to RAF inhibition via reversible induction of a slowly dividing de-differentiated state. Mol. Syst. Biol..

[33] Xue Y., Martellotto L., Baslan T., Vides A., Solomon M., Mai T.T., Chaudhary M., Riely G., Li B., Scott K. (2017). An approach to suppress the evolution of resistance in BRAF^V600E^-mutant cancer. Nat. Med..

[34] Lu H., Liu S., Zhang G., Wu B., Zhu Y., Frederick D., Hu Y., Zhong W., Randell S., Sadek N., Zhang W. (2017). PAK signaling drives acquired drug resistance to MAPK inhibitors in BRAF-mutant melanomas. Nature.

[35] Hachey S.J., Boiko A.D. (2016). Therapeutic implications of melanoma heterogeneity. Exp. Dermatol..

[36] Tirosh I., Izar B., Prakadan S., Wadsworth M., Treacy D., Trombetta J., Rotem A., Rodman C., Lian C., Murphy G. (2016). Dissecting the multicellular ecosystem of metastatic melanoma by single-cell RNA-seq. Science.

[37] Manca A., Lissia A., Capone M., Ascierto P.A., Botti G., Caracò C., Stanganelli I., Colombino M., Sini M.C., Cossu A. (2015). Activating PIK3CA mutations coexist with BRAF or NRAS mutations in a limited fraction of melanomas. J. Transl. Med..

[38] Berger M., Hodis E., Hefferman T., Deribe Y.L., Lawrence M.S., Protopopov A., Ivanova E., Watson I.R., Nickerson E., Ghosh P. (2012). Melanoma genome sequencing reveals frequent PREX2 mutations. Nature.

[39] Nikolaev S.I., Rimoldi D., Iseli C., Valsesia A., Robyr D., Gehrig C., Harshman K., Guipponi M., Bukach O., Zoete V. (2012). Exome sequencing identifies recurrent somatic MAP2K1 and MAP2K2 mutations in melanoma. Nat. Genet..

[40] Stark M.S., Woods S.L., Gartside M., Bonazzi V.F., Dutton-Regester K., Aoude L.G., Chow D., Sereduk C., Niemi N.M., Tang N. (2012). Frequent somatic mutations in MAP3K5 and PAP3K9 in metastatic melanoma identified by exome sequencing. Nat. Genet..

[41] Wei X., Walla V., Lin J.C., Teer J.K., Prickett T.D., Gartner J., Davis S., Stemke-Hale K., Davies M.A., Gershenwald G.E. (2011). Exome sequencing identifies GRIN2A as frequently mutated in melanoma. Nat. Genet..

[42] Ceol C.J., Houvras Y., Jane-Valbuena J., Bilodeau S., Orlando D.A., Battisti V., Fritsch L., Lin W.M., Hollmann T.J., Ferré F. (2011). The histone methyltransferase SETDB1 is recurrently amplified in melanoma and accelerates its onset. Nature.

[43] Macgregor S., Montgomery G.M., Liu J.Z., Zhao Z.Z., Henders A.K., Stark M., Schmid H., Holland E.A., Duffy D.L., Zhang M. (2011). Genome-wide association study identifies a new melanoma susceptibility locus at 1q21.3. Nat. Genet..

[44] Miura S., Maesawa C., Shibazaki M., Yasuhira S., Kasai S., Tsunoda K., Maeda F., Takahashi K., Akasaka T., Masuda T. (2014). Immunohistochemistry for histone H3 lysine 9 methyltransferase and demethylase proteins in human melanomas. Am. J. Dermatopathol..

[45] Prickett T., Agrawal N., Wei X., Yates K.E., Lin J.C., Wunderlich J., Cronin J.C., Cruz P., Rosenberg S.A., Samuels Y. (2009). Analysis of the tyrosine kinase in melanoma reveals recurrent mutations in ERBB4. Nat. Genet..

[46] Wong S.Q., Behren A., Mar V.J., Woods K., Li J., Martin C., Sheppard K.E., Wolfe R., Kelly J., Cebon J. (2014). Whole genome sequencing identifies a recurrent RQCD1 P131L mutation in cutaneous melanoma. Oncotarget.

[47] Pleasance E.D., Cheetham R.K., Stephens P.J., McBride D.J., Humphray S.J., Greenman C.D., Varela I., Lin M.L., Ordóñez G.R., Bignell G.R. (2010). A comprehensive catalogue of somatic mutations from a human cancer genome. Nature.

[48] Hodis E., Watson I.R., Kryukov G.V., Arold S.T., Imielinski M., Theurillat J.P., Nickerson E., Auclair D., Li L., Place C., Dicara D. (2012). A landscape of driver mutations in melanoma. Cell.

[49] Krauthammer M., Kong Y., Ha B.H., Evans P., Bacchiocchi A., McCusker J.P., Cheng E., Davis M.J., Goh G., Choi M. (2012). Exome sequencing identifies recurrent somatic RAC1 mutations in melanoma. Nat. Genet..

[50] Aydin I.T., Melamed R.D., Adams S.J., Castillo-Martin M., Demir A., Bryk D., Brunner G., Cordon-Cardo C., Osman I., Rabadan R. (2014). FBXW7 mutations in melanoma and a new therapeutic paradigm. J. Natl. Cancer. Inst..

[51] Cao J., Ge M.H., Ling Z.Q. (2016). Fbxw7 tumor suppressor: A vital regulator contributes to human tumorigenesis. Medicine.

[52] Kourtis N., Moubarak R.S., Aranda-Rrgille B., Lui K., Aydin I.T., Trimarchi T., Darvishian F., Salvaggio C., Zhong J., Bhatt K. (2015). FBXW7 modulates cellular stress response and metastatic potential through HSF1 post-translational modification. Nat. Cell Biol..

[53] Aydin I.T., Abbate F., Rajan G.S., Badal B., Aifantis I., Desman G., Celebi J.T. (2017). FBXW7 inactivation in a Braf^V600E^-driven mouse model leads to melanoma development. Pigment Cell Melanoma Res..

[54] Lovly C.M., Dahlman K.B., Fohn L.E., Su Z., Dias-Santagata D., Hicks D.J., Hucks D., Berry E., Terry C., Duke M. (2012). Routine multiplex mutational profiling enables enrollment in genotype-driven therapeutic trials. PLoS ONE.

[55] Dahlman K.B., Xia J., Hutchinson K., Ng C., Hucks D., Jia P., Atefi M., Su Z., Branch S., Lyle P.L. (2012). BRAFL597 mutations in melanoma are associated with sensitivity to MEK inhibitors. Cancer Discov..

[56] Utchinson K.E., Lipson D., Stephens P.J., Otto G., Lehmann B.D., Lyle P.L., Vnencak-Jones C.L., Ross J.S., Pietenpol J.A., Sosman J.A. (2013). BRAF fusions define a distinct molecular subset of melanomas with potential sensitivity to MEK inhibition. Clin. Cancer Res..

[57] Xia J., Jia P., Hutchinson K., Dahlman K.D., Johnson D., Sosman J., Pao W., Zhao Z. (2014). A meta-analysis of somatic mutations from next generation sequencing of 241 melanomas: A road map for the study of genes with potential clinical relevance. Mol. Cancer Ther..

[58] Mar V.J., Wong S.Q., Li J., Scolyer R.A., McLean C., Papenfuss A.T., Tothill R.W., Kakavand H., Mann G.J., Thompson J.F. (2013). BRAF/NRAS wild-type melanomas have a high mutation load correlating with histologic and molecular signatures of UV damage. Clin. Cancer Res..

[59] Kwong L.N., Costello J.C., Liu H., Jiang S., Helms T.L., Langsdorf A.E., Jakubosky D., Genovese G., Muller F.L., Jeong J.H. (2012). Oncogenic NRAS signaling differentially regulates survival and proliferation in melanoma. Nat. Med..

[60] Dai D.L., Martinka M., Li G. (2005). Prognostic significance of activated Akt expression in melanoma: A clinopathologic study of 292 cases. J. Clin. Oncol..

[61] Vredeveld L., Possi K.P., Smit M., Meissl K., Michaloglou C., Horlings M.H., Ajouaou A., Kortman P.C., Dankort D., McMahon M. (2012). Abrogation of BRAF^V600E^-induced senescence by PI3K pathway activation contributes to melanomagenesis. Genes. Dev..

[62] Karreth F.A., Tay Y., Perna D., Ala U., Tan S.M., Rust A.G., DeNicola G., Webster K.A., Weiss D., Perez-Mancera P.A. (2011). In vivo identification of tumor-suppressive PTEN ceRNAs in an oncogenic BRAF-induced mouse model of melanoma. Cell.

[63] Namiki T., Yaguchi T., Nakamura K., Valencia J., Coelho S., Yin L., Kawaguchi M., Vieira W., Keneko Y., Tanemura A. (2015). NUAK2 amplification coupled with PTEN deficiency promotes melanoma development via CDK activation. Cancer Res..

[64] Beadling C., Jacobson-Dunlop E., Hodi F.S., Le C., Warrick A., Patterson J., Town A., Harlow A., Cruz F., Azar S. (2008). KIT gene mutations and copy number in melanoma subtypes. Clin. Cancer Res..

[65] Vayssa C., Pautier C., Fillerou T., Maisongrosse V., Rodier J.F., Lavoue V., Reyal F., Thomas L., de la Fouchardière A., Delannes M. (2013). A large retrospective multicenter study of vaginal melanomas: Implications for new management. Melanoma Res..

[66] Kong Y., Si L., Zhu Y., Xu X., Corless C.L., Flaherty K.T., Li L., Li H., Sheng X., Cui C. (2011). Large-scale analysis of KIT aberrations in chinese patients with melanoma. Clin. Cancer Res..

[67] Minor D.R., Kashani-Sahet M., Garrido M., O’Day S.J., Hamid O., Bastian B.C. (2012). Sunitinib therapy for melanoma patients with kit mutations. Clin. Cancer Res..

[68] Hansen R., Abildgaard C., Riber-Hansen R., Steiniche T., Lade-Keller J., Gulberg P. (2015). KIT is a frequent target for epigenetic silencing in cutaneous melanoma. J. Investig. Dermatol..

[69] Neiswender J., Kortum R., Bourque C., Kasheta M., Zon L., Morrison D., Ceol C. (2017). KIT suppresses BRAF^V600E^-mutant melanoma by attenuating oncogenic RAS/MAPK signaling. Cancer Res..

[70] Puig-Butille J.A., Badens C., Ogbah Z., Carrera C., Aguilera P., Malvehy J., Puig S. (2013). Genetic regulation in Ras-regulated pathways in acral lentiginous melanoma. Exp. Dermatol..

[71] Liang W.S., Hendricks W., Kiefer J., Schmidt J., Sekar S., Carpten J., Craig D., Adkins J., Cuyugan L., Manojlovic Z. (2017). Integrated genomic analyses reveal frequent TERT aberrations in acral melanoma. Gen. Res..

[72] Kang H.J., Cui Y., Yin H., Scheid A., Hendricks W.P., Schmidt J., Sekulic A., Kong D., Trent J.M., Gokhale V. (2017). A pharmacological chaperone molecule induces cancer cell death by restoring tertiary DNA structures in mutant hTERT promoters. J. Am. Chem. Soc..

[73] Huang F., Hodis E., Xu M., Kryukov G.V., Chin L., Garraway L.A. (2013). Highly recurrent TERT promoter mutations in human melanoma. Science.

[74] Horn S., Figl A., Rachakonda S., Fischer C., Sucker A., Gast A., Kadel S., Moll I., Nagore E., Hemminki K. (2013). TERT promoter mutations in familial and sporadic melanoma. Science.

[75] Ofner R., Ritter C., Heidenreich B., Kumar R., Ugurel S., Schrama D., Becker J.C. (2017). Distribution of TERT promoter mutations in primary and metastatic melanomas in Austrian patients. J. Cancer Res. Clin. Oncol..

[76] Griewank K.G., Murali R., Puig-Batille J.A., Schilling B., Livingstone E., Potrony M., Carrera C., Schimming T., Möller I., Schwamborn M. (2014). TERT promoter mutation status as an independent prognostic factor in cutaneous melanoma. J. Natl. Cancer Inst..

[77] Seynnaeve B., Lee S., Borah S., Park Y., Pappo A., Kirkwood J.M., Bahrami A. (2017). Genetic and epigenetic alterations of TERT are associated with inferior outcome in adolescent and young adult patients with melanoma. Sci. Rep..

[78] Werhold N., Jacobsen A., Schultz N., Sander C., Lee W. (2014). Genome-wide analysis of noncoding regulatory mutations in cancer. Nat. Genet..

[79] Li Y., Cheng H.S., Chng W.J., Tergaonkar V. (2016). Activation of mutant TERT promoter by RAS-ERK signaling is a key step in malignant progression of BRAF-mutant human melanomas. Proc. Natl. Acad. Sci. USA.

[80] Lian C.G., Xu Y., Ceol C., Wu F., Larson A., Dresser K., Xu W., Tan L., Hu Y., Zhan Q. (2012). Loss of 5-Hydroxymethyl-cytosine is an epigenetic hallmark of melanoma. Cell.

[81] Pan W., Zhu S., Qu K., Meeth K., Cheng J., He K., Ma H., Liao Y., Wen X., Roden C. (2017). The DNA methylcytosine dioxygenase Tet2 sustains immunosuppressive function of tumor-infiltrating myeloid cells to promote melanoma progression. Immunity.

[82] Gembarska A., Luciani F., Fedele C., Russell E.A., Dewaele M., Villar S., Zwolinska A., Haupt S., de Lange J., Yip D., Goydos J. (2012). MDM4 is a key therapeutic target in cutaneous melanoma. Nat. Med..

[83] Dewaele M., Tabaglio T., Willekens K., Bezzi M., Teo S.X., Low D., Koh C., Rambow F., Fiers M., Rogiers A. (2016). Antisense oligonucleotide-mediated MDM4 exon 6 skipping impairs tumor growth. J. Clin. Investig..

[84] Dar A., Majid S., Rittstenter C., de Semir D., Bezrookove V., Tong S., Nosrati M., Sagebiel R., Miller J.R., Kashani-Sabet M. (2013). The role of miR-18b in MDM2-p53 pathway signaling and melanoma progressing. J. Natl. Cancer. Inst..

[85] De Polo A., Luo Z., Gerarduzzi C., Chen X., Little J.B., Yuan Z.M. (2017). AXL receptor signalling suppresses p53 in melanoma through stabilization of the MDMX-MDM2 complex. J. Mol. Cell. Biol..

[86] The Cancer Genome Atlas Network (2015). Genomic classification of cutaneous melanoma. Cell.

[87] Krauthammer M., Kong Y., Bacchiocchi A., Evans P., Pornputtapong N., Wu C., McCusker J.P., Ma S., Cheng E., Straub R. (2015). Exome sequencing identifies recurrent mutations in NF1 and RASopathy genes in sun-exposed melanomas. Nat. Genet..

[88] Maertens O., Johnson B., Hollstein P., Frederick D.T., Cooper Z.A., Messiaen L., Bronson R.T., McMahon M., Granter S., Flaherty K. (2013). Elucidating distinct roles for NF1 in melanomagenesis. Cancer Discov..

[89] Whittaker S.R., Theurillat J.P., Van Allen E., Wagle N., Hsiao J., Cowley G.S., Schadendorf D., Root D.E., Garraway L.A. (2013). A genome scale RNA interference screen implicates NF1 loss in resistance to RAF inhibition. Cancer Discov..

[90] Cirenajwis H., Lauss M., Ekedahl H., Torngren T., Kvist A., Saal L., Olsson H., Staaf J., Carneiro A., Ingvar C. (2017). NF1-mutated melanoma tumors harbor distinct clinical and biological characteristics. Mol. Oncol..

[91] Hayward N.K., Wilmott J.S., Waddell N., Johansson P.A., Field M.A., Nones K., Patch A.M., Kakavand H., Alexandrov L., Burke H. (2017). Whole-genome landscapes of major melanoma subtypes. Nature.

[92] Kong Y., Sheng X., Wu X., Yan J., Ma M., Yu J., Si L., Chi Z., Cui C., Dai J. (2017). Frequent genetic aberrations in the CDK4 pathway in acral melanoma indicate the potential for CDK4/6 inhibitors in targeted therapy. Clin. Cancer Res..

[93] Lu C., Zhang J., Nagahawatte P., Easton J., Lee S., Liu Z., Ding L., Wyczalkowski M., Valentine M., Navid F. (2015). The genomic landscape of childhood and adolescent melanoma. J. Investig. Dermatol..

[94] Seab J.A., Graham J.H., Helwig E.B. (1989). Deep penetrating nevus. Am. J. Surg. Pathol..

[95] Yeh I., Lang U., Durieux E., Tee M.K., Jorapur A., Shain H., Hadda V., Pissaloux D., Chen X., Cerroni L. (2017). Combined activation of MAP kinase pathway and β-catenin signaling cause deep penetrating nevi. Nat. Commun..

[96] Poollock P.M., Harper U.L., Hausen K.S., Yudt L.M., Stark M., Robbins C.M., Moses T.Y., Hostetter G., Wagner U., Kakareka J. (2003). High frequency of BRAF mutations in nevi. Nat. Genet..

[97] Shain H., Yeh I., Kovalyshyn I., Sriharan A., Talevich E., Gagnon A., Dummer R., North J., Pincus L., Ruben B. (2015). The genetic evolution of melanoma from precursor lesions. N. Engl. J. Med..

[98] Chiba K., Lorbeer F., Shain H., McSwiggen D., Schruf E., Oh A., Ryu J., Darzacq X., Bastian B., Hockemeyer D. (2017). Mutations in the promoter of the telomerase gene TERT contribute to tumorigenesis by a two-step mechanism. Science.

[99] Emirogly N., Yildiz P., Biyik Ozkaya D., Bahali A.G., Su O., Ousun N. (2017). Evolution of Spitz nevi. Pediatr. Dermatol..

[100] Wiesner T., He J., Yelensky R., Esteve-Puig R., Botton T., Yeh I., Lipson D., Otto G., Brennan K., Murali R. (2014). Kinase fusions are frequent in Spitz tumors and spitzoid melanomas. Nat. Commun..

[101] Yeh I., Tee M.K., Botton B., Shain A.H., Sparatta A.J., Gagnon A., Vemula S.S., Garrido M.C., Nakamaru K., Isoyama T. (2016). NTRK3 kinase fusions in Spitz tumours. J. Pathol..

[102] Amin S.M., Haugh A.M., Lee C.Y., Zhang B., Bubley J.A., Merkel E.A., Verzi A.E., Gerami P. (2017). A comparison of morphologic and molecular features of BRAF, ALK, and NTRK1 fusion spitzoid neoplasms. Am. J. Surg. Pathol..

[103] Lazova R., Pornputtapong N., Halaban R., Bosenberg M., Bai Y., Chai H., Krauthammer M. (2017). Spitz nevi and spitzoid melanomas: Exome sequencing and comparison with conventional melanocytic nevi and melanomas. Mod. Pathol..

[104] Yeh I., Botton T., Talevich E., Shain A.H., Sparatta A.J., de la Fouchardiere A., Mully T.W., North J.P., Garrido M.C. (2016). Activating MET kinase rearrangements in melanoma and Spitz tumors. Nat. Commun..

[105] Lee S., Barnhill R.L., Dummer R., Dalton J., Wu J., Pappo A., Bahrami A. (2015). TERT promoter mutations are predictive of aggressive clinical behavior in patients with spitzoid melanocytic neoplasms. Sci. Rep..

[106] Wu G., Barnhill R.L., Lee S., Li Y., Shao Y., Easton Y., Dalton J., Zhang J., Pappo A., Bahrami A. (2016). The landscape of fusion transcripts in spitzoid melanoma and biologically indeterminate spitzoid tumors by RNA sequencing. Mod. Pathol..

[107] Busam K.J., Vilain R.E., Lum T., Busam J.A., Hollmann T.J., Saw R.P., Coit D.C., Scollyer R.A., Wiesner T. (2016). Primary and metastatic cutaneous melanomas express ALK through alternative transcriptional initiation. Am. J. Surg. Pathol..

[108] Wiesner T., Lee W., Obenauf A.C., Ran L., Murali R., Zhang Q.F., Wong E.W., Hu W., Scott S.N., Shah R.H. (2015). Alternative transcription initiation leads to expression of a novel ALK isoform in cancer. Nature.

[109] Tetzlaff M.T., Reuben A., Billings S.D., Prieto V.G., Curry J.L. (2017). Toward a molecular-genetic classification of Spitzoid neoplasms. Clin. Lab. Med..

[110] Wiesner T., Kutzner H., Cerroni L., Mihm M.C., Busam K.J., Murali R. (2016). Genomic aberrations in spitzoid melanocytic tumors and their implications for diagnosis, prognosis and therapy. Pathology.

[111] Shalin S. (2017). A review of kinase fusions in melanocytic tumors. Lab. Investig..

[112] Yeh I., de la Fouchardiere A., Pissaloux D., Mully T.W., Garrido M.C., Vemula S.S., Busam K.J., LeBoit P.E., McCalmont T.H. Clinical, histopathologic, and genomic features of Spitz tumors with ALK fusions. Am. J. Surg. Pathol..

[113] Hewkes J.E., Truong A., Meyer L. (2016). Genetic predisposition to melanoma. Semin. Oncol..

[114] Mangas C., Potrany M., Mainetti C., Bianchi E., Carozza Merlani P., Mancarella Eberhardt A., Maspoli-Postizzi E., Marazza G., Marcollo-Pini A. (2016). Genetic susceptibility to cutaneous melanomas in southern Switzerland: Role of CDKN2A, MCIR and MTIF. Br. J. Dermatol..

[115] Law M.H., Bishop D.T., Lee J.E., Beassard M., Martin N.G., Moses E.K., Song F., Barrett J.H., Kimar R., Easton D.F. (2016). Genome-wide meta-analysis identifies five new susceptibility loci for cutaneous malignant melanoma. Nat. Genet..

[116] Choi J., Xu M., Makowski M., Zhang T., Law M.H., Kovacs M.A., Granzhan A., Kim W.J., Parikh H., Gartside M. (2017). A common intronic variant of PARP1 confers melanoma risk and mediates melanocyte growth via regulation of MITF. Nat. Genet..

[117] Valverde P., Healy E., Jackson I., Rees J.L., Thody A.J. (1995). Variants of the melanocyte-stimulating hormone receptor gene are associated with red hair and fair skin in humans. Nat. Genet..

[118] Beaumont K.A., Shekar S.N., Cook A.L., Duffy D.L., Sturm R.A. (2008). Red hair is the null phenotype of MC1R. Hum. Mutat..

[119] Healy E., Flannagan N., Ray A., Todd C., Jackson I.J., Matthews J.N.S., Birch-Machin M.A., Rees J.L. (2000). Melanocortin-1-receptor gene and sun sensitivity in individuals without red hair. Lancet.

[120] Swope V., Abdel-Malek Z.A. (2014). Significance of the melanocortin 1 receptor in the DNA damage response of human melanocytes to ultraviolet radiation. Pigment Cell Melanoma Res..

[121] Bishop D.T., Demenais F., Iles M.M., Harland M., Taylor J.C., Corda E., Randerson-Moor J., Aitken J.F., Avril M.F., Azizi E. (2009). Genome-wide association study identifies three loci associated with melanoma risk. Nat. Genet..

[122] Robles-Espinoza C.D., Roberts N.D., Chen S., Leacy F.P., Alexandrov L.B., Pornputtapong N., Halaban R., Krauthammer M., Cui R., Timothy Bishop D. (2016). Germline MC1 status influences somatic mutations burden in melanoma. Nat. Commun..

[123] Chen S., Zhu B., Liu W., Han C., Chen B., Liu T., Li X., Chen X., Li C., Hu L. (2017). Palmitoylation-dependent activation of MC1R prevents melanomagenesis. Nature.

[124] Tailor N.T., Mitra N., Goldstein A.M., Tucker M.A., Aviril M.F., Azizi E., Bergman W., Bishop D.T., Bressac-de-Pailleretes S., Bruno W. (2017). Germline variation at CDKN2A and associations with nevus phenotypes among members of melanoma families. J. Investig. Dermatol..

[125] Helgadottir H., Touminen R., Olsson H., Hansson J., Hoiom V. (2017). Cancer risks and survival in patients with multiple primary melanomas: Association with family history of melanoma and germline CDKN2A mutation status. J. Am. Acad. Dermatol..

[126] Bonet C., Luciani F., Ottavi J.F., Leclerc J., Jouenne F.M., Boncompagni M., Bille K., Hofman V., Bossis G., Marco de Donatis G. (2017). Deciphering the role of oncogenic MITF^E318K^ in senescence delay and melanoma progression. J. Natl. Cancer Inst..

[127] Van Reamsdonk C.D., Berrookove V., Green G., Bauer J., Gaugler L., O’Brien J.M., Simpson E.M., Barsh G.S., Bastian B.C. (2009). Frequent somatic mutations of GNAQ in uveal melanoma and blue naevi. Nature.

[128] Yoo J.H., Shi D.S., Groissmann A.H., Sorensen L.K., Tong Z., Mleynek T.M., Rogers A., Zhu W., Richards J.R., Winter J.M. (2016). ARF6 is an actionable node that orchestrates oncogenic GNAQ signaling in uveal melanoma. Cancer Cell.

[129] Van Raamsdonk C.D., Griewank K.G., Crosby M.B., Garrido M.C., Vemula S., Wiesner T., Obenauf A.C., Wackernagel W., Green G., Bouvier N. (2010). Mutations in GNA11 in uveal melanoma. N. Engl. J. Med..

[130] Harbour J.W., Onken M.D., Robinson E.D., Duan S., Cao L., Worley L.A., Council M.L., Matatall K.A., Helms C., Bowcock A.M. (2010). Frequent mutation of BAP1 in metastasizing uveal melanomas. Science.

[131] Wiesner T., Obenauf A., Murali R., Fried I., Griewank K.G., Ulz P., Windpassinger C., Wackernagel W., Loy S., Wolf I. (2011). Germline mutations in BAP1 predispose to melanocytic tumors. Nat. Genet..

[132] Abdel-Mahuran M.H., Pilarski R., Cebulla C.M., Massenill J.B., Christopher B.N., Boru G., Hovalnd P., Davidorf F.H. (2011). Germline BAP1 mutation predispose sto uveal melanoma, lung adenocarcinoma, meningioma, and other cancers. J. Med. Genet..

[133] Harbour J.W. (2012). The genetics of uveal melanoma: An emerging framework for targeted therapy. Pigment Melanoma Cell Res..

[134] Royer-Bertrand B., Torsello M., Rimoldi D., El Zaoui I., Cisarova K., Pescin-Gobert R., Raynaud F., Zografos L., Schalenbourg A., Speiser D. (2016). Comprehensive genetic landscape of uveal melanoma by whole-genome sequencing. Am. J. Hum. Gen..

[135] Prescher G. (1996). Prognostic implications of monosomy 3 in uveal melanoma. Lancet.

[136] Martin M., MaBhofer L., Temming P., Rahmann S., Metz C., Bornfeld N., van de Nes J., Klein-Hitpass L., Hinnebusch A.G., Horsthemke B. (2013). Exome sequencing identifies recurrent somatic mutations in EIF1AX and SF3B1 in uveal melanoma with disomy 3. Nat. Genet..

[137] Harbour J.W., Roberson E., Anbunathan H., Onken M.D., Worley L.A., Bowcock A.M. (2013). Recurrent mutations at codon 625 of the splicing factor SF3B1 in uveal melanoma. Nat. Genet..

[138] Alsafadi S., Houy A., Battistella A., Popova T., Wassef M., Henry E., Tirode F., Constantinou A., Piperno-Neumann S., Roman-Roman S. (2016). Cancer-associated SF3B1 mutations affect alternative splicing by promoting alternative branchpoint usage. Nat. Commun..

[139] Yavuzygitoglu S., Koopmans A.E., Verdijk R.M., Vaarwater J., Eussen B., van Bodegom A., Paridaens D., Kiliç E., de Klein A. (2016). Uveal melanomas with SF3B1 mutations: A distinct subclass associated with late-onset metastases. Ophtalmology.

[140] Kusters-Vandelvelde H.V., Creytens D., van Engen-van Grunvsen A.C., Jeunink M., Winnepenninckx V., Groenen P.J., Küsters B., Wesseling P., Blokx W.A., Prinsen C.F. (2016). S3BF1 and EIF1AX mutations occur in primary leptomeningeal melanocytic neoplasms. Arch. Neuropathol. Commun..

[141] Moore A.R., Ceraudo E., Sher J.J., Guan Y., Shoushtari A.N., Chang M.T., Zhang J.Q., Walczak E.G., Kazmi M.A., Taylor B.S. (2016). Recurrent activating mutations of G-protein-coupled receptor CYSLTR2 in uveal melanoma. Nat. Genet..

[142] Johansson D., Aoude L.G., Wadt K., Glasson W.J., Warrier S.K., Hewitt A.W., Kiilgaard J.F., Heegaard S., Isaacs T., Franchina M. (2015). Deep sequencing of uveal melanoma identifies a recurrent mutation in PLCB4. Oncotarget.

[143] Decatur C.L., Ong E., Garg N., Anbunathan H., Bowocock A.M., Field M.G., Harbour J.W. (2016). Driver mutations in uveal melanoma: Associations with gene expression profile and patient outcomes. JAMA Ophtalmol..

[144] Field M.G., Decatur C.L., Kurtenbach S., Gezgin G., van der Velden P.A., Jager M.J., Kozak K.N., Harbour J.W. (2016). PRAME as an independent biomarker for metastasis in uveal melanoma. Clin. Cancer. Res..

[145] Robertson A.G., Shih J., Yau C., Gibb E., Oba J., Mungall K., Hess J., Uzunangelov V., Walter V., Danilova L. (2017). Integrative analysis identifies four molecular and clinical subsets in uveal melanoma. Cancer Cell.

[146] Pandiani C., Beranger G., Leclerc J., Ballotti R., Bertolotto C. (2017). Focus on cutaneous and uveal melanoma specificities. Genes Dev..

[147] Cosgarea I., Ugurel S., Sucker A., Livingstone E., Zimmer L., Ziemer M., Utikal J., Mohr P., Pfeiffer C., Pfohler C. (2017). Targeted next generation sequencing of mucosal melanomas identifies frequent NF1 and RAS mutations. Oncotarget.

[148] Griewank K.G., Westekemper H., Murali R., Mach M., Schilling B., Wiesner T., Schimming T., Livingstone E., Sucker A., Grabellus F., Metz C. (2013). Conjunctival melanomas harbour BRAF and NRAS mutations and copy number changes similar to cutaneous melanomas. Clin. Cancer Res..

[149] Hintzsche J.D., Garden N.T., Amato C.M., Kim J., Wuensch K.E., Robinson S.E., Applegate A.J., Couts K.L., Medina T.M., Wells K.R., Wisell J.A. (2017). Whole-exome sequencing identifies recurrent SF3B1 R625 mutation and mutations of NF1 and KIT in mucosal melanoma. Melanoma Res..

[150] Griewank K.G., Murali R., Schilling B., Scholz S., Sucker A., Song M., Susskind D., Grabelluls F., Zimmer L., Hillen U. (2013). TERT promoter mutations in ocular melanoma distinguish between conjunctival and uveal tumors. Br. J. Cancer.

[151] Hou J., Baptiste C., Hombalegowda R.B., Tergas A., Feldman R., Jones N., Chetterjee-Paer S., Bus-Kwoflski A., Wright J.D., Burke W.M. (2017). Vulvar and vaginal melanoma: A unique subclass of mucosal melanoma based on a comprehensive molecular analysis of 2253 cases of nongynecological melanoma. Cancer.

[152] Shain A.H., Garrido M., Botton T., Tavelich E., Yeh I., Sanborn J.Z., Chung J., Wang N.J., Kalavand H., Mann G. (2015). Exome sequencing of desmoplastic melanoma identifies recurrent NFKBIE promoter mutations and diverse activating mutations in the MAPK pathway. Nat. Genet..

[153] Eroglu Z., Kim D.W., Johnson D.B. (2015). Response to anti-PD1/PDL1 therapy in patients with metastatic desmoplastic melanoma. J. Clin. Oncol..

[154] Frydenlund N., Leone D., Yang S., Hoang M.P., Deng A., Hernandez-Perez M., Singh R., Biswas A., Yaar R., Mahalingam M. (2017). Tumoral PD-L1 expression in desmoplastic melanoma is associated with depth invasion, tumor-infiltrating CD8 cytotoxic lymphocytes and mixed cytomorphological variant. Mod. Pathol..

[155] Kadokoura A., Frydenlund N., Leone D.A., Yang S., Hoang M.P., Hernandez-Perez M., Biswas A., Singh R., Yaar R., Mahalingam M. (2016). Neurofibromin protein loss in desmoplastic melanoma subtypes: Implicating NF1 allelic loss as a distinct genetic driver?. Hum. Pathol..

[156] Yang S., Leone D., Frydenlund N., Hoang M., Derng A., Hernandez-Perez M., Biswas A., Singh R., Yaar R., Mahalingam M. (2016). Frequency of telomerase reverse transcripter promoter mutations in desmoplastic melanoma subtypes: Analyses of 76 cases. Melanoma Res..

[157] Kiuru M., Busam K.J. (2017). The NF1 gene in tumor syndromes and melanoma. Lab. Investig..

[158] Halaban R., Krauthammer M. (2016). Rasopathy gene mutations in melanoma. J. Investig. Dermatol..

[159] Palla B., Su A., Binder S., Dry S. (2013). SOX10 expression distinguishes desmoplastic melanoma from its histologic mimics. Am. J. Dermopathol..

[160] Tacha D., Qi W., Ra S., Bremer R., Yu C., Chu J., Hoang L., Robbins B. (2015). A newly developed mouse monoclonal SOX10 antibody is a highly sensitive and specific marker for malignant melanoma, including spindle cell and desmoplastic melanomas. Arch. Pathol. Lab. Med..

[161] Pavlova O., Fraitag S., Hohl D. (2016). 5-hydroxymethylcytosine expression in proliferative nodules arising within congenital nevi allows differentiation from malignant melanoma. J. Investig. Dermatol..

[162] Gong F., Guo Y., Niu Y., Jin J., Zhang X., Shi X., Zhang L., Li R., Chen L., Ma R.Z. (2017). Epigenetic silencing of TET2 and TET3 induces an EMT-like process in melanoma. Oncotarget.

[163] Micevic G., Theodosakis N., Bosenberg M. (2017). Aberrant DNA methylation in melanoma: Biomarker and therapeutic opportunities. Clin. Epigenetics.

[164] Lauss M., Haq R., Cirenajwis H., Phung B., Harbst K., Staaf J., Rosengren F., Holm K., Aine M., Jistrom K. (2015). Genome-wide DNA methylation analysis in melanoma reveals the importance of CpG methylation in MITF regulation. J. Investig. Dermatol..

[165] Wouters J., Vizoso M., Martinez-Cardus A., Carmona F.J., Govaere O., Laguna T., Joseph J., Dynoodt P., Aura C., Foth M. (2017). Comprehensive DNA methylation study identifies novel progression-related and prognostic markers for cutaneous melanoma. BMC Med..

[166] Fiziev P., Akdemir K.C., Miller J.P., Keung E., Samant N., Sharma S., Natale C., Terranoiva C., Maitituoheti M., Amin S. (2017). Systemic epigenomic analysis reveals chromatic states associated with melanoma progression. Cell Rep..

[167] Badal B., Solovyov A., Di Cecilia S., Chan J.M., Chang L.W., Iqbal R., Aydin I., Rayan G., Chen C., Abbate F. (2017). Transcriptional dissection of melanoma identifies a high-risk subtype underlying TP53 family genes and epigenome deregulation. JCI Insight.

[168] Jonsson G., Busch C., Knappskog S., Geisler J., Miletic H., Ringner M., Lillehaug J.R., Borg A., Lonning P.E. (2010). Gene expression profiling-based identification of molecular subtypes in stage IV melanomas with different clinical outcome. Clin. Cancer Res..

[169] Harbst K., Staaf J., Lauss M., Karlsson A., Masback A., Johasson I., Bendahl P.O., Vallon-Christersson J., Tomgren T., Ekedahl H. (2012). Molecular profiling reveals low- and high-grade forms of primary melanoma. Clin. Cancer Res..

[170] Nsengimana J., Laye J., Filia A., Walker C., Jewell R., Van den Oord J.J., Wolter P., Patel P., Sucker A., Schadendorf D. (2015). Independent replication of a melanoma subtype gene signature and evaluation of its prognostic value and biological correlates in a population cohort. Oncotarget.

[171] Cirenajwis H., Ekedahl H., Lauss M., Harbst K., Carneiro A., Enoksson J., Rosengren F., Werner-Hartman L., Torngren T., Kvist A. (2015). Molecular stratification of metastatic melanoma using gene expression profiling: Prediction of survival outcome and benefit from molecular targeted therapy. Oncotarget.

[172] Lauss M., Nsengimana J., Staaf J., Newton-Bishop J., Jonsson G. (2016). Consensus of melanoma gene expression subtypes converges on biological entities. J. Investig. Dermatol..

[173] Lardone R., Plaisier S., Navarrete M., Shamonki J., Jalas J., Sieling P., Lee D. (2016). Cross-platform comparison of independent datasets identifies an immune signature associated with improved survival in metastatic melanoma. Oncotarget.

[174] Wongchenko M., McArthur G., Dreno B., Larkin J., Ascierto P., Sosman J., Andries L., Kockx M., Hurst S., Caro I. (2017). Gene expression profiling in BRAF-mutated melanoma patients reveals patient subgroups with poor outcomes to Vemurafenib that may be overcome by Cobimetinib plus Vemurafenib. Clin. Cancer Res..

[175] Hugo W., Zaretsky J.M., Sun L., Song C., Moreno B.H., Hu-Lieskovan S., Berent-Maoz B., Pang J., Chmielowski B., Cherry G. (2016). Genomic and transcriptomic features of response to anti-PD-1 therapy in metastatic melanoma. Cell.

[176] Lauss M., Ringner M., Karlsson A., Harbst K., Busch C., Geisler J., Lonning P.E., Staaf J., Jonsson G. (2015). DNA methylation subgroups in melanoma are associated with proliferative and immunological processes. BMC Med. Genom..

[177] Morab B., Silva R., Perry A.S., Gallagher W.M. (2017). Epigenetics of malignant melanoma. Semin. Cancer Biol..

[178] Harbst K., Staaf J., Masback A., Olsson H., Ingvar C., Vallon-Christersson J., Ringner M., Borg A., Jonsson G. (2010). Multiple metastases from cutaneous malignant melanoma patients may display heterogeneous genomic and epigenomic patterns. Melanoma Res..

[179] Harbst K., Lauss M., Crenajwis H., Winter C., Howlin J., Tomgren T., Kvist A., Nodin B., Olsson E., Hakkinen J. (2014). Molecular and genetic diversity in the metastatic process of melanoma. J. Pathol..

[180] Sanborn J.Z., Chung J., Purdom E., Wang N.J., Lakavand H., Wilmott J., Butler T., Thompson J., Mann G., Haydu L. (2015). Phylogenetic analyses of melanoma reveal complex patterns of metastatic dissemination. Proc. Natl. Acad. Sci. USA.

[181] Harbst K., Lauss M., Cirenajwis H., Isaksson K., Rosengren F., Torngren T., Kvist A., Johansson M., Vallon-Christersson J., Baldetorp B. (2016). Multiregion whole-exome sequencing uncovers the genetic evolution and mutational heterogeneity of early-stage metastatic melanoma. Cancer Res..

[182] Muller J., Krijgsman O., Tsoi J., Robert L., Song C., Kong X., Possik P.A., Cornelissen-Steijger P.D., Geukes Foppen M.H., Kemper K. (2014). Low MITF/AXL ratio predicts early resistance to multiple targeted drug in melanoma. Nat. Commun..

[183] Konieczkowski D.J., Johannessen C.M., Abudayyeh O., Kim J.W., Copper Z.A., Piris A., Frederick D.T., Barzily-Rokni M., Starussman R., Haq R. (2014). A melanoma cell state distinction influences sensitivity to MAPK pathway inhibitors. Cancer Discov..

[184] Ennen M., Keime C., Gambi G., Kieny A., Coassolo S., Thibault-Carpentier C., Margerin-Schaller F., Davidson G., Vagne C., Lipsker D. (2017). MITF-high and MITF-low cells and a novel subpopulation expressing genes of both cell states contribute to intra and inter-tumoral heterogeneity of primary melanoma. Clin. Cancer Res..

[185] Li J., Yan S., Liu Z., Zhou Y., Pan Y., Yuan W., Liu M., Tan Q., Tian G., Dong B. (2017). Multiregional sequencing reveals genomic alterations and clonal dynamics in primary malignant melanoma of the esophagus. Cancer Res..

[186] Shaffer S.M., Dunagin M.C., Torborg S.R., Torre E.A., Emert B., Krepler C., Beqiri M., Sproesser K., Brafford P.A., Xiao M. (2017). Rare cell variability and drug-induced reprogramming as a mode of cancer drug resistance. Nature.

[187] Perez-Guijarro E., Day C.P., Merlino G., Zaidi M.R. (2017). Genetically engineered mouse models of melanoma. Cancer.

[188] Day C.P., Marchalik R., Merlino G., Michael H. (2017). Mouse models of UV-induced melanoma: Genetics, pathology, and clinical relevance. Lab. Investig..

[189] Hacker E., Irwin N., Muller H.K., Powell M.B., Kay G., Hayward N., Walker G. (2005). Neonatal ultraviolet radiation exposure is critical for malignant melanoma induction in pigmented Tpras transgenic mice. J. Investig. Dermatol..

[190] Ackermann J., Frutschi M., Kaloulis K., McKee T., Trumpp A., Beermann F. (2005). Metastasizing melanoma formation caused by expression of activated N-RasQ61K on an INK4a-deficient background. Cancer Res..

[191] Dhomen N., Reis-Filho J.S., da Rocha-Dias S., Hayward R., Savage K., Delmas V., Larue L., Pritchard C., Marais R. (2009). Oncogenic Braf induces melanocyte senescence and melanoma in mice. Cancer Cell.

[192] Goel V.K., Ibrahim N., Jiang G., Singhal M., Fee S., Flotte T., Westmoreland S., Haluska F.S., Hinds P.W., Haluska F.G. (2009). Melanocytic nevus-like hyperplasia and melanoma in transgenic BRAFV600E mice. Oncogene.

[193] Dankort D., Curley D.P., Cartlidge R.A., Nelson B., Karnezis A.N., Damsky W.E., You M.J., DePinho R.A., McMahon M., Bosenberg M. (2009). BRAF^V600E^ cooperates with Pten loss to induce metastatic melanoma. Nat. Genet..

[194] Kaufman C.K., Mosimann C., Fan Z.P., Yang S., Thomas A.J., Ablain J., Tan J.L., Fogley R.D., van Rooijen E., Hagedorn E.J. (2016). A zebrafish melanoma model reveals emergence of neural crest identity during melanomas initiation. Science.

[195] Mort R.L., Jackson I.J., Patton E.E. (2015). The melanocyte lineage in development and disease. Development.

[196] Glover J.D., Knolle S., Wells K.L., Liu D., Jackson I.J., Mort R.L., Headon D.J. (2015). Maintenance of distict melanocyte populations in the interfollicular epidermis. Pigment Cell Melanoma Res..

[197] Kohler C., Nittner D., Rambow F., Radaelli E., Stanchi F., Vandamme N., Baggiolini A., Sommer L., Berx G., van den Oord J.J. (2017). Mouse cutaneous melanoma induced by mutant BRaf arises from expansion and dedifferentiation of mature pigmented melanocytes. Cell Stem Cell.

[198] Moon H., Donahue L.R., Choi E., Scumpia P.O., Lowry W.E., Grenier J.K., Zhu J., White A.C. (2017). Melanocyte stem cell activation and translocation initiate cutaneous melanoma in response to UV exposure. Cell Stem Cell.

[199] Mohania D., Chendel S., Kumar P., Verma V., Digvijak K., Tripathi D., Choudhury K., Mitten S.K., Shah D. (2017). Ultraviolet radiations: Skin defense-damage mechanism. Adv. Exp. Med. Biol..

[200] Harris A.L., Joseph R.W., Copland J.A. (2016). Patient-derived tumor xenograft models for melanoma drug discovery. Exp. Opin. Drug. Discov..

[201] Einarsdottir B.O., Bagge R.O., Bhadury J., Jespersen H., Mattsson J., Nilsson L.M., Truvé K., López M.D., Naredi P., Nilsson O. (2014). Melanoma patient-derived xenografts accurately model the disease and develop fast enough to guide treatment decisions. Oncotarget.

[202] Flaherty K.T., Puzanov I., Kim K.B., Ribas A., McArthur G.A., Sosman J.A., O’Dwyer P.J., Lee R.J., Grippo J.F., Nolop K. (2010). Inhibition of mutated, activated BRAF in metastatic melanoma. N. Engl. J. Med..

[203] Das Thakur M., Salangsang F., Landman A.S., Sellers W.R., Pryer N.K., Levesque M.P., Dummer R., McMahon M., Stuart D.D. (2013). Modelling vemurafenib resistance in melanoma reveals a strategy to forestall drug resistance. Nature.

[204] Bollag G., Hirth P., Tsai J., Zhang J., Ibrahim P.N., Cho H., Spevak W., Zhang C., Zhang Y., Habets G. (2010). Clinical efficacy of a RAF inhibitor need broad target blockade in BRAF-mutated melanoma. Nature.

[205] Fang D., Nguyen T.K., Leishear K., Finko R., Kulp A.N., Hotz S., Van Belle P.A., Xu X., Elder D.E., Herlyn M. (2005). A tumorigenic subpopulation with stem cell properties in melanomas. Cancer Res..

[206] Schmidt P., Kopecky C., Hombach A., Zigrino P., Mauch C., Abken H. (2011). Eradication of melanomas by targeted elimination of a minor subset of tumor cells. Proc. Natl. Acad. Sci. USA.

[207] Schmidt P., Abken K. (2011). The beating heart of melanomas: A great minor subset of cancer cells sustains tumor growth. Oncoterget.

[208] Pinc A., Somasundaram R., Wagner C., Hörmann M., Karanikas G., Jalili A., Bauer W., Brunner P., Grabmeier-Pfistershammer K., Gschaider M. (2012). Targeting CD20 in melanoma patients at high risk of disease recurrence. Mol. Ther..

[209] Schlaak M., Schmidt P., Bangard C., Kurschat P., Mauch C., Abken H. (2012). Regression of metastatic melanoma by targeting cancer stem cells. Oncotarget.

[210] Song H., Su X., Yang K., Niu F., Li J., Song J., Chen H., Li B., Li W., Qian W. (2015). CD20 antibody-conjugated immunoliposomes for targeted chemotherapy of melanoma cancer initiating cells. J. Biomed. Nanothechnol..

[211] Somasundaram R., Zhang G., Fukunaga-Kalabis M., Perego M., Krepler C., Xu X., Wagner C., Hristova D., Zhang J., Tian T. (2017). Tumor-associated B-cells induce tumor heterogeneity and therapy resistance. Nat. Commun..

[212] Monzani E., Facchetti F., Galmozzi E., Corsini E., Benetti A., Cavazzin C., Gritti A., Piccinini A., Porro D., Santinami M. (2007). Melanoma contains CD133 and ABCG2 positive cells with enhanced tumorigenic potential. Eur. J. Cancer.

[213] Klein W.M., Wu B.P., Zhao S., Wu H., Klein-Szanto A.J., Tahan S.R. (2007). Increased expression of stem cell markers in malignant melanoma. Mod. Pathol..

[214] Rappa G., Fodstad O., Lorico A. (2008). The stem cell-associated antigen CD133 (Prominin-1) is a molecular therapeutic target for metastatic melanoma. Stem Cells.

[215] Schatton T., Murphy G.F., Frank N.Y., Yamaura K., Waaga-Gasser A.M., Gasser M., Zhan Q., Jordan S., Duncan L.M., Weishaupt C. (2008). Identification of cells initiating melanomas. Nature.

[216] Botelho M.G., Wang X., Arndt-Jovin D.J., Becker D., Jovin T.M. (2010). Induction of terminal differentiation in melanoma cells on downregulation of beta-amyloid precursor protein. J. Investig. Dermatol..

[217] Keshet G.I., Goldstein I., Itzhaki O., Cesarkas K., Shenhav L., Yakirevitch A., Treves A.J., Schachter J., Amariglio N., Rechavi G. (2008). MDR1 expression identifies human melanoma stem cells. Biochim. Biophys. Res. Commun..

[218] Chartrain M., Riond J., Stennevin A., Vandenberghe I., Gomes B., Lamant L., Meyer N., Gairin J.E., Guilbaud N., Annereau J.P. (2012). Melanoma chemotherapy leads to the selection of ABCB5-expressing cells. PLoS ONE.

[219] Frank N.Y., Mangaryan A., Huang Y., Schatton T., Waaga-Gasser A.M., Gasser M., Sayegh M.H., Sadee W., Frank M.H. (2005). ABCB5-mediated doxorubicin transport and chemoresistance in human malignant melanoma. Cancer Res..

[220] Lai C.Y., Schwartz B.E., Hsu M.Y. (2012). CD133^+^ melanoma subpopulations contribute to perivascular niche morphogenesis and tumorigenesis through vasculogenic mimicry. Cancer Res..

[221] Frank N., Schatton T., Kim S., Zhan Q., Wilson B.J., Ma J., Saab K.R., Osherov V., Widlund H.R., Gasser M. (2011). VEGF-R1 expressed by malignant melanoma-initiating cells is required for tumor growth. Cancer Res..

[222] Kupas V., Weishaupt C., Siepmann D., Kaserer M.L., Eickelmann M., Metze D., Luger T.A., Beissert S., Loser K. (2011). RANK is expressed in metastatic melanoma and highly upregulated on melanoma-initiating cells. J. Investig. Dermatol..

[223] Touil Y., Zuliani T., Woloczuk I., Kuranda K., Prochazkova J., Andrieux J., Le Roy H., Mortier L., Vandomme J., Jouy N. (2013). The PI3K/AKT signaling pathway controls the quiescence of the low-rhodamine 123-retention cells compartment essential for melanoma stem cells. Stem Cells.

[224] Setia N., Abbas O., Sousa Y., Garb J.L., Mahalingam M. (2012). Profiling of ABC transporters ABCB5, ABCF2 and enstin-positive stem cells in nevi, in situ and invasive melanoma. Mod. Pathol..

[225] Lin J.Y., Zhang M., Schatton T., Wilson B.J., Alloo A., Ma J., Qureshi A.A., Frank N.Y., Han J., Frank M.K. (2013). Genetically determined ABCB5 functionality correlates with pigmentation phenotype and melanoma risk. Biochem. Biophys. Res. Commun..

[226] Wilson B.J., Saab K.R., Ma J., Schatton T., Pütz P., Zhan Q., Murphy G.F., Gasser M., Waaga-Gasser A.M., Frank N.Y. (2014). ABCB5 maintains melanoma-initiating cells through a pro-inflammatory cytokine signaling circuit. Cancer Res..

[227] Wang S., Tang L., Lin J., Shen Z., Yao Y., Wang W., Tao S., Gu C., Ma J., Xie Y., Liu Y. (2017). ABCB5 promotes melanoma metastasis through enhancing NF-kB p65 protein stability. Biochem. Biophys. Res. Commun..

[228] Quintana E., Shackleton M., Sabel M.S., Fullen D.R., Johnson T.M., Morrison S.J. (2008). Efficient tumor formation by single human melanoma cells. Nature.

[229] Prasmickaite L., Skrbo N., Hoifodt H.K., Suo Z., Engebråten O., Gullestad H.P., Aamdal S., Fodstad Ø., Mælandsmo G.M. (2010). Human malignat melanoma harbours a large fraction of highly clonogenic cells that do not express markers associated with cancer stem cells. Pigment Cell Melanoma Res..

[230] Zhong Y., Guan K., Zhou C., Ma W., Wang D., Zhang Y., Zhang S. (2010). Cancer stem cells sustaining the growth of mouse melanoma are not rare. Cancer Lett..

[231] Boiko A.D., Razorenova O.V., van de Rijn M., Swetter S.M., Johnson D.L., Ly D.P., Butler P.D., Yang G.P., Joshua B., Kaplan M.J. (2010). Human melanoma-initiating cells express neural crest nerve growth factor receptor CD271. Nature.

[232] Civenni G., Walter A., Kobert N., Mihic-Probst D., Zipser M., Belloni B., Seifert B., Moch H., Dummer R., van den Broek M. (2011). Human CD271-positive melanoma stem cells associated with metastasis establish tumor heterogeneity and long-term growth. Cancer Res..

[233] Valyi-Nagi K., Kormos B., Ali M., Shulka D., Valyi-Nagy T. (2012). Stem cell marker CD271 is expressed by vascular mimicry-priming uveal melanoma cells in three-dimensional cultures. Mol. Vis..

[234] Kumar S.M., Liu S., Lu H., Zhang H., Gimotty P.A., Guerra M., Guo W., Xu X. (2012). Acquired cancer stem cell phenotypes through Oct4-mediated dedifferentiation. Oncogene.

[235] Redmer T., Welte Y., Behrens D., Fichtner I., Przybilla D., Wruck W., Yaspo M.L., Lehrach H., Schäfer R., Regenbrecht C.R. (2014). The nerve growth factor receptor CD271 is crucial to maintain tumorigenicity and stem-like properties of melanoma cells. PLoS ONE.

[236] Bayle S.E., Fedele C.G., Corbin V., Wybacz E., Szeto P., Lewin J., Young R.J., Wong A., Fuller R., Spillane J. (2016). CD271 expression on patient melanoma cells is unstable and unlinked to tumorigenicity. Cancer Res..

[237] Ngo M., Han A., Lakatos A., Sahoo D., Hachey S.J., Weiskopf K., Beck A.H., Weissman I.L., Boiko A.D. (2016). Antibody therapy targeting CD47 and CD271 effectively suppresses melanoma metastasis in patient-derived xenografts. Cell Rep..

[238] Cheli Y., Giuliano S., Botton T., Rocchi S., Hofman V., Bahadoran P., Bertolotto C., Ballotti R. (2011). Mitf is the key molecular switch between mouse or human melanoma initiating cells and their differentiated progeny. Oncogene.

[239] Cheli Y., Bonnazi V., Jacquel A., Allegra M., De Donatis G.M., Bahadoran P., Bertolotto C., Ballotti R. (2014). CD271 is an imperfect marker for melanoma initiating cells. Oncotarget.

[240] Radke J., Robner F., Redmer T. (2017). CD271 determines migratory properties of melanoma cells. Sci. Rep..

[241] Redmer T., Walz I., Kliunger B., Khouja S., Welte Y., Schafer R., Regenbrecht C. (2017). The role of the cancer stem cell marker CD271 in DNA damage response and drug resistance of melanoma cells. Oncogenesis.

[242] Roesch A., Fukunaga-Kabalis M., Schmidt E., Zabierowski S.E., Brafford P.A., Vultur A., Basu D., Gimotty P., Vogt T., Herlyn M. (2010). A temporally distinct subpopulation of slow-cycling melanoma cells is required for continuous tumor growth. Cell.

[243] Quintana E., Schackleton M., Foster H.R., Fullen D.R., Sabel M.S., Johnson T.M., Morrison S.J. (2010). Phenotypic heterogeneity among tumorigenic melanoma cells from patients that is reversible and nor hierarchically organized. Cancer Cell.

[244] Prasmiekhaite L., Engesaeter B.O., Skrbo N., Hellenes T., Kristian A., Oliver N.K., Suo Z., Mælandsmo G.M. (2010). Aldehyde dehydrogenase (ALDH) activity does not select for cells with enhanced aggressive properties in malignant melanoma. PLoS ONE.

[245] Amann P.M., Hofmann C., Freudenberger M., Holland-Cunz S., Eichmüller S.B., Bazhin A.V. (2012). Expression and activity of alcohol and aldehyde dehydrogenases in melanoma cells and in melanocytes. J. Cell. Biochem..

[246] Boonyaratanakornkit J.B., Yue L., Strachan L.R., Scalapino K.J., LeBoit P.E., Lu Y., Leong S.P., Smith J.E., Ghadially R. (2010). Selection of melanoma tumorigenic cells using ALDH. J. Investig. Dermatol..

[247] Santini R., Vinci M.C., Pandolfi S., Penachioni J.Y., Montagnani V., Olivito B., Gattai R., Pimpinelli N., Gerlini G., Borgognoni L. (2012). HEDGEHOG-GLI signaling drives self-renewal and tumorigenicity of human melanoma-initiating cells. Stem Cells.

[248] Luo Y., Dallaglio K., Chen Y., Robinson S.E., McCarter M.D., Wang J., Gonzalez R., Thompson D.C., Norris D.A. (2012). ALDH1A isozymes are markers of human melanoma stem cells and potential therapeutic targets. Stem Cells.

[249] Perez-Alea M., McGrail K., Sanchez-Redondo S., Ferrer B., Fournet G., Cortés J., Munoz E., Hernandez-Losa J., Tenbaum S., Martin G. (2017). ALDH1A3 is epigenetically regulated during melanocyte transformation and is a target for melanoma treatment. Oncogene.

[250] Taghizadeh R., Noh M., HuH Y., Ciusani E., Sigalotti L., Maio M., Arosio B., Nicotra M.R., Natali P., Sherley J.L. (2010). CXCR6, a newly defined biomarker of tissue-specific stem cell asymmetric self-renewal, identifies more aggressive human melanoma cancer stem cells. PLoS ONE.

[251] Kalabis M.F., Martinez G., Nguyen T.K., Kim D., Santiago-Walker A., Roesch A., Herlyn M. (2010). Tenascin-C promotes melanoma progression by maintaining the ABCB5-positive side population. Oncogene.

[252] Shakhova O., Ziugg D., Schaefer S.M., Hari L., Civenni G., Blunschi J., Claudinot S., Okoniewski M., Beermann F., Mihic-Probst D. (2012). Sox10 promotes the formation and maintenance of giant congenital naevi and melanoma. Nat. Cell. Biol..

[253] Shakhova O., Cheng P., Mishra P.J., Zingg D., Schaefer S.M., Debbache J., Hausel J., Matter C., Guo T., Davis S. (2015). Antagonistic cross-regulation between Sox9 and Sox10 controls an anti-tumorigenic program in melanoma. PLoS Genet..

[254] Alonso-Curbelo D., Riveiro-Falkenbach E., Perez-Guijarro E., Cifdaloz M., Karras P., Osterloh L., Megías D., Cañón E., Calvo T.G., Olmeda D. (2014). RAB7 controls melanoma progression by exploiting a lineage-specific wiring of the endolysosomal pathway. Cancer Cell.

[255] Santini R., Pietrobono S., Pandolfi S., Montagnani V., D’Amico M., Penachioni J.Y., Vinci M.C., Borgognoni L., Stecca B. (2014). SOX2 regulates self-renewal and tumorigenesis of human melanoma-initiating cells. Oncogene.

[256] Hoek K., Eichoff D.M., Schlegel M.C., Döbbeling U., Kobert N., Schaerer L., Hemmi S., Dummer R. (2008). In vivo switching of human melanoma cells between proliferative and invasive states. Cancer Res..

[257] Goodall J., Carreira S., Denat L., Kobi D., Davidson L., Nuciforo P., Sturm R.A., Larue L., Goding C.R. (2008). Brn-2 represses microphtalmia-associated transcription factor expression and marks a distinct subpopulation of microphtalmia-associated transcription factor-negative melanoma cells. Cancer Res.

[258] Thurber A.E., Douglas G., Sturm E.C., Zabierowski S.E., Smit D.J., Ramakrishnan S.N., Hacker E., Leonard J.H., Herlyn M., Sturm R.A. (2011). Inversion expression states of the BRN2 and MITF transcription factors in melanoma spheres and tumor xenografts regulate NOTCH pathway. Oncogene.

[259] Pinner S., Jordan P., Sharrock K., Bazley L., Collinson L., Marais R., Bonvin E., Goding C., Sahai E. (2009). Intravital imaging reveals transient changes in pigment production and Brn2 expression during metastatic melanoma dissemination. Cancer Res..

[260] Arozarena I., Sanchez-Laorden B., Packer L., Hidalgo-Carcedo C., Hayward R., Viros A., Sahai E., Marais R. (2011). Oncogenic BRAF induces melanoma cell invasion by downregulating the cGMP-specific phosphodiesterase PDE5A. Cancer Cell.

[261] Ahn A., Chatterjee A., Eccles M.R. (2017). The slow cycling phenotype: A growing problem for treatment resistance in melanoma. Mol. Cancer Ther..

[262] Perego M., Maurer M., Wang J.K., Shaffer S., Muller A.C., Parapatics K., Li L., Hristova D., Shin S., Keeney F. (2017). A slow-cycling subpopulation of melanoma cells with highly invasive properties. Oncogene.

[263] Pascual G., Avgustinova A., Mejetta S., Martin M., Castellanos A., Attolini C.S., Berenguer A., Prats N., Toll A., Bescos C. (2017). Targeting metastasis-initiating cells through the fatty acid receptor CD36. Nature.

[264] Bonvin E., Falletta P., Show H., Delmas V., Goding C.R. (2012). A PI3K-Pax3 axis regulates BRN-2 expression in melanoma. Mol. Cell. Biol..

[265] Cheli Y., Giuliano S., Fenouille N., Allegra M., Hofman V., Hofman P., Bahadoran P., Lacour J.P., Tartare-Deckert S., Bertolotto C. (2012). Hypoxia and MITF control metastatic behaviour in mouse and human melanoma cells. Oncogene.

[266] Romano S., Staibano S., Greco A., Brunetti A., Nappo G., Ilardi G., Martinelli R., Sorrentino A., Di Pace A., Mascolo M. (2013). FK506 binding protein 51 positively regulates melanoma stemness and metastatic potential. Cell Death Dis..

[267] Strizzi L., Margaryan N.V., Gilgur A., Hardy K.M., Normanno N., Salomon D.S., Hendrix M.J.C. (2013). The significance of a Cripto-1-positive subpopulation of human melanoma cells exhibiting stem cell-like characteristics. Cell Cycle.

[268] Kleffel S., Posch C., Barthel S., Mueller H., Schlapbach C., Guenova E., Elco C., Lee N., Juneja V., Zhan Q. (2015). Melanoma cell-intrinsic PD-1 receptor functions promote tumor growth. Cell.

[269] Quintana E., Piskounova E., Shackleton M., Weinberg D., Eskiocak U., Fullen D.R., Johnson T.M., Morrison S.J. (2012). Human melanoma metastasis in NSG mice correlates with clinical outcome in patients. Sci. Transl. Med..

[270] Held M.A., Curley D.P., Dankort D., McMahon M., Muthusamy V., Bosenberg M.W. (2010). Characterization of melanoma cells capable of propagating tumors from a single cell. Cancer Res..

[271] Fukunaga-Kalabis M., Roesch A., Herlyn M. (2011). From cancer stem cells to tumor maintenance in melanoma. J. Investig. Dermatol..

[272] Landsberg J., Kohlmeyer J., Renn M., Bald T., Rogava M., Cron M., Fatho M., Lennerz V., Wölfel T., Hölzel M. (2012). Melanomas resist T-cell therapy through inflammation-induced reversible differentiation. Nature.

[273] Gammaitoni L., Giraudo L., Leuci V., Todorovic M., Mesiano G., Picciotto F., Pisacane A., Zaccagna A., Volpe M.G., Gallo S. (2013). Effective activity of cytokine-induced killer cells against autologous metastatic melanoma, including cells with stemness features. Clin. Cancer Res..

[274] Ostyn P., El Machhour R., Bagard S., Kotecki N., Vandomme J., Flamenco P., Segard P., Masselot B., Formstecher P., Touil Y. (2014). Transient TNF regulates the self-renewing capacity of stem-like label-retaining cells in sphere and skin equivalent models of melanoma. Cell Commun. Signal..

[275] Alix-Panabieres C., Pantel K. (2014). Challenges in circulating tumour cell research. Nat. Rev. Cancer.

[276] Luo X., Mitra D., Sullivan R.J., Wittner B.S., Kimura A.M., Pan S., Hoang M.P., Brannigan B.W., Lwarence D.P., Flaherty K.T. (2014). Isolation and molecular characterization of circulating melanoma cells. Cell Rep..

[277] Sarioglu A.F., Aceto N., Kojic N., Donalson M.C., Zeinali M., Hanza B., Engstrom A., Zhu H., Sundaresan T.K., Miyamoto D.T. (2015). A microfluidic device for label-free, physical capture of circulating tumor cell clusters. Nat. Methods.

[278] Ozkumur E., Shah A.M., Ciciliano J.C., Emmink B.L., Miyamoto D.T., Brachel E., Yu M., Chen P.I., Morgan B., Trautwein J. (2013). Inertial focusing for tumor-antigen-dependent and-independent sorting of rare circulating tumor cells. Sci. Transl. Med..

[279] Aya-Bonilla C., Marvasela G., Freeman J., Lomma C., Frank M., Khattak M., Meniawy T., Millward M., Warkiani M., Gray E. (2017). Isolation and detection of circulating tumour cells from metastatic melanoma patients using a slanted spiral microfluidic device. Oncotarget.

[280] Girotti M.R., Gremel G., Lee R., Galvani E., Rothwell D., Viros A., Mandal A.K., Lim K.H., Saturno G., Furney S. (2016). Application of sequencing, liquid biopsies, and patient-derived xenografts for personalized medicine in melanoma. Cancer Discov..

[281] Kandhelwal G., Girotti M.R., Smowton C., Taylor S., With C., Dynowki M., Frese K., Brady G., Dive C., Marais R. (2017). Next-generation sequencing analysis and algorithmas for PDX and CDX models. Mol. Cancer Res..

[282] Calapre L., Warburton L., Millward M., Zimn M., Gray E.S. (2017). Circulating tumor DNA (ctDNA) as a liquid biopsy for melanoma. Cancer Lett..

[283] Santiago-Walker A., Gagnon R., Mazumdar J., Casey M., Long G.V., Schadendorf D., Flaherty K., Kefford R., Hauschild A., Hwu P. (2016). Correlation of BRAF mutation status in circulating-free DNA and tumor and association with clinical outcome across four BRAFi and MEKi clinical trials. Clin. Cancer Res..

[284] Gangadhar T.C., Savitch S.L., Yee S.S., Xu W., Huang A.C., Harmon S., Lieberman D.B., Soucier D., Fan R., Black T.A. (2017). Feasibility of monitoring advancer melanoma patients using cell-free DNA from plasma. Cancer Res..

[285] Gremel G., Lee R.J., Girotti M.R., Mandal A.K., Valpione S., Garner G., Ayub M., Wood S., Rothwell D.G., Fusi A. (2016). Distinct subclonal tumour responses to therapy revealed by circulating cell-free DNA. Ann. Oncol..

[286] Luke J.J., Flaherty K.T., Ribas A., Long G.V. (2017). Targeted agents and immunotherapiues: Optimizinbg outcomes in melanoma. Nat. Rev. Clin. Oncol..

[287] Wei S.C., Levine J.H., Cogdill A.P., Zhao Y., Anang N.A., Andrews M.C., Sharma P., Wang J., Wargo J.A., Péer D. (2017). Distinct cellular mechanisms underlie anti-CTLA-4 and anti-PD1 checkpoint blockade. Cell.

[288] Huang A.C., Postow M.A., Orlowski R.T., Mick R., Bengsch B., Manne S., Xu M., Harmon S., Giles J.R., Wenz B. (2017). T cell invigoration to tumor burden ratio associated with anti-PD-1 response. Nature.

[289] Schadendorf D., Hodi F.S., Robert C., Weber J.S., Margolin K., Hamid O., Patt D., Chen T.T., Berman D.M., Wolchok J.D. (2015). Pooled analysis of long-term survival data from phase II and phase III trials of ipilimumab in unresectable or metastatic melanoma. J. Clin. Oncol..

[290] Eggermont A.M., Chiarion-Sileni V., Grob J.J., Dummer R., Wolchok J.D., Schmidt H., Hamid O., Robert C., Ascierto P.A., Richards J.M. (2016). Prolonged survival in stage III melanoma with ipilimumab adjuvant therapy. N. Engl. J. Med..

[291] Coens C., Suciu S., Chiarion-Sileni V., Grob J.J., Dummer R., Wolchok J.D., Schmidt H., Hamid O., Robert C., Ascierto P.A. (2017). Health-related quality of life with adjuvant ipilimumab versus placebo after complete resection of high-risk stage III melanoma (EORTC 18071): Secondary outcomes of a multinational, randomised, double-blind, phase 3 trial. Lancet Oncol..

[292] Zhang S., Liang F., Li W., Wang Q. (2017). Risk of treatment-related mortality in cancer patients treated with ipilimumab: A systematic review and meta-analysis. Eur. J. Cancer.

[293] Ascierto P.A., Del Vecchio M., Robert C., Mackiewicz A., Chiarion-Sileni V., Arance A., Lebbé C., Bastholt L., Hamid O., Rutkowski P. (2017). Ipilimumab 10 mg/kg versus ipilimumab 3 mg/kg in patients with unresectable or metastatic melanoma: A randomised, double-blind, multicentre, phase 3 trial. Lancet Oncol..

[294] Weber J., Mandala M., Del Vecchio M., Gogas H.J., Arance A.M., Cowey C.L., Dalle S., Schenker M., Chiarion-Sileni V., Marquez-Rodas I. (2017). Adjuvant nivolumab bersus ipilimumab in resected stage III or IV melanoma. N. Engl. J. Med..

[295] Weber J.S., D’Angelo S.P., Minor D., Hodi F.S., Gutzmer R., Neyns B., Hoeller C., Khushalani N.I., Miller W.H., Lao C.D. (2015). Nivolumab versus chemotherapy in patients with advanced melanoma who progressed after anti-CTLA-4 treatment (CheckMate 037): A randomised, controlled, open-label, phase 3 trial. Lancet Oncol..

[296] Robert C., Schachter J., Long G.V., Arance A., Grob J.J., Mortier L., Daud A., Carlino M.S., McNeil C., Lotem M. (2015). Pembrolizumab versus ipilimumab in advanced melanoma. N. Engl. J. Med..

[297] Lin Z., Chen X., Li Z., Luo Y., Fang Z., Xu B., Han M. (2016). PD-1 antibody monotherapy for malignant melanoma: A systematic review and meta-analysis. PLoS ONE.

[298] Larkin J., Minor D., D’Angelo S., Neyns B., Smylie M., Miller W.H., Gutzmer R., Lenette G., Chmielowski B., Lao C.D. (2017). Overall survival in aptients with advanced melanoma who received Nivolumab versus investigator’s choice chemotherapy in CheckMate 037: A randomized, controlled, open-label phase III trial. J. Clin. Oncol..

[299] Postow M.A., Chesney J., Pavlick A.C., Robert C., Grossmann K., McDermott D., Linette G.P., Meyer N., Giguere J.K., Agarwala S.S. (2015). Nivolumab and Ipilimumab versus Ipilimumab in untreated melanoma. N. Engl. J. Med..

[300] Postow M., Chesney J., Pavlick A., Robert C., Grossmann K., McDermott D., Linette G., Meyer N., Giguere J., Agarwala S. Initial report of overall survival rates from a randomized phase II trial evaluating the combination of Nivolumab (NIVO) and Ipilimumab (IPI) in patients with advanced melanoma (MEL) [abstract]. Proceedings of the 107th Annual Meeting of the American Association for Cancer Research (AACR).

[301] Wolchok J.D., Chiarion-Sileni V., Gonzalez R., Rutkowski P., Grob J.J., Cowey C.L., Lao C.D., Wagstaff D., Schadendorf D., Ferrucci P.F. (2017). Overall survival with combined nivolumab and ipilimumab in advanced melanoma. N. Engl. J. Med..

[302] Larkin J., Hodi F.S., Wolchok J.D. (2015). Combined nivolumab and ipilimumab or monotherapy in untreated melanoma. N. Engl. J. Med..

[303] Wolchok J.D., Chiarion-Sileni V., Gonzalez R., Rutkowski P., Grob J.J., Cowey L., Lao C., Schadendorf D., Ferrucci P.F., Smyle M. (2016). Updated results from a phase III trial of nivolumab (NIVO) combined with ipilimumab (IPI) in treatment-naïve patients (pts) with advanced melanoma (MEL) (CheckMate 067). J. Clin. Oncol..

[304] Goldstein D.A. (2017). Adjuvant ipilimumab for melanoma-The $1.8 million per patient regimen. JAMA Oncol..

[305] Schadendorf D., Wolchok J.D., Hodi F.S., Chiarion-Sileni V., Gonzalez R., Rutkowski P., Grob J.J., Cowey C.L., Lao C.D., Chesney J. (2017). Efficacy and safety outcomes in patients with advanced melanoma who discontinued treatment with nivolumab and ipilimumab because of adverse events: A pooled analysis of randomized phase II and III trials. J. Clin. Oncol..

[306] Snyder A., Makarov V., Merghoub T., Yuan J., Zaretsky J.M., Desrichard A., Walsh L.A., Postow M.A., Wong P., Ho T.S. (2014). Genetic basis for clinical response to CTLA-4 blockade in melanoma. N. Engl. J. Med..

[307] Van Allen E.M., Miao D., Schilling B., Shukla S.A., Blank C., Zimmer L., Sucker A., Hillen U., Foppen M.H.G., Goldinger S.M. (2015). Genomic correlates of response to CLTA-4 blockade in metastatic melanoma. Science.

[308] Tumeh P.C., Harview C.L., Yearley J.H., Shintaku I.P., Taylor E.J., Robert L., Chmielowski B., Spasic M., Henry G., Ciobanu V. (2014). PD-1 blockade induces responses by inhibiting adaptive immune resistance. Nature.

[309] Taube J.M., Klein A., Brahmer J.R., Xu H., Pan X., Kim J.H., Chen L., Pardoll D.M., Topalian S.L., Anders R.A. (2014). Association of PD-1, PD-1 ligands, and other features of the tumor immune microenvironment with response to anti-PD-1 therapy. Clin. Cancer Res..

[310] Gubin M.M., Zhang X., Schuster H., Caron E., Ward J.P., Noguchi T., Ivanova Y., Hundal J., Arthur C.D., Krebber W.J. (2014). Checkpoint blockade cancer immunotherapy targets tumor-specific mutant antigens. Nature.

[311] Mezzadra R., Sun C., Jae L., Gomez-Eerland R., de Vries E., Wu W., Logtenberg M., Slagter M., Rozeman E., Hofland I. (2017). Identification of CMTM6 and CMTM4 as PD-L1 protein regulators. Nature.

[312] Burr M., Sparbier C., Chan Y.C., Williamson J., Woods K., Beans P.A., Lam E., Henderson M.A., Bell C.C., Stolzenburg S. (2017). CMTM6 maintains the expression of PD-L1 and regulates anti-tumour immunity. Nature.

[313] Das R., Verma R., Sznol M., Boddupalli C.S., Gettinger S.N., Kluger H., Callahan M., Wolchok J.D., Halaban R., Dhodapkar M.V. (2015). Combination therapy with anti-CTLA-4 and anti-PD-1 leads to distinct immunologic changes in vivo. J. Immunol..

[314] Koyama S., Akbay E.A., Li Y.Y., Herter-Sprie G.S., Buczkowski K.A., Richards W.G., Gandhi L., Redig A.J., Rodig S.J., Asahina H. (2016). Adaptive resistance to therapeutic PD-1 blockade is associated with upregulation of alternative immune checkpoints. Nat. Commun..

[315] Riaz N., Havel J., Makarov V., Desrichard A., Urba W., Sims J., Hodi S., Martin-Desrichard A., Mandal R., Sharfman W. (2017). Tumor and microenvironment evolution during immunotherapy with Nivolumab. Cell.

[316] Rooney M.S., Shukla S.A., Wu C.J., Gets G., Hacohen N. (2015). Molecular and genetic properties of tumors associated with local immune cytolytic activity. Cell.

[317] Spranger S., Bao R., Gajewski T.F. (2015). Melanoma-intrinsic beta-catenin signalling prevents anti-tumor immunity. Nature.

[318] Chen P.L., Roh W., Reuben A., Cooper Z.A., Spencer C.N., Prieto P.A., Miller J.P., Bassett R.L., Gopalakrishnan V., Wani K. (2016). Analysis of immune signatures in longitudinal tumor samples yelds insight into biomarkers of response and mechanisms of resistance to immune checkpoint blockade. Cancer Discov..

[319] Sucker A., Zhao F., Pieper N., Heeke C., Maltaner R., Stadtler N., Real B., Bielefeld N., Howe S., Weide B. (2017). Acquired IFNγ resistance impairs anti-tumor immunity and gives rise to T-cell-resistant melanoma lesions. Nat. Commun..

[320] Ayers M., Lunceford J., Nebozhyn M., Murphy E., Loboda A., Kaufman D.R., Albright A., Cheng J.D., Kang S.P., Shankaran V. (2017). IFN-γ-related mRNA profile predicts clinical response to PD-1 blockade. J. Clin. Investig..

[321] Patel S.J., Sanjana N.E., Kishton R.J., Eidaseh A., Veduala S.K., Can M., Gartner J., Jie L., Steinberg S., Yamamoto T. (2017). Identification of essential genes for cancer immunotherapy. Nature.

[322] Zaretsky J.M., Garcia-Diaz A., Shin D.S., Escuin-Ordinay H., Hugo W., Hu-Lieskovan S., Torrejon D.Y., Abril-Rodriguez G., Sandoval S., Barthly L. (2016). Mutations associated with acquired resistance to PD-1 blockade in melanoma. N. Engl. J. Med..

[323] Schumacher T.N., Schreiber R.D. (2017). Neontigens in cancer immunotherapy. Science.

[324] Ott P.A., Hu Z., Kerskin D., Shukla S., Sun J., Bozym D., Zhang W., Luoma A., Giobbie-Hurder A., Peter L. (2017). An immunogenic personal neoantigen vaccine for patients with melanoma. Nature.

[325] Spitzer M.H., Carmi Y., Reticher-Flynn N., Kwek S., Madhireddy D., Martins M., Gherardini P.F., Prestwoood T., Chabon J., Bendall S. (2017). Systemic immunity is required for effective cancer immunotherapy. Cell.

[326] Dummer R., Schadendorf D., Ascierto P.A., Arance A., Dutriaux C., Di Giacomo A.M., Routkowski P., Del Vecchio M., Gutzemer R., Mandala M. (2017). Binimetinib versus dacarbazine in patients with advanced NRAS-mutant melanoma (NEMO): A multicentre, open-label, randomised, phase 3 trial. Lancet Oncol..

[327] Johnson D.B., Lovly C., Flavin M., Panageas K., Ayers G., Zhao Z., Iams W., Colgan M., De Noble S., Terry C. (2015). Impact of NRAS mutations for patients with advanced melanoma treated with immune therapies. Cancer Immnol. Res..

[328] Kirchberger M.C., Ugurel S., Mangana J., Heppt M., Eigentler T., Berking C. (2017). NRAS-mutated melanoma patients have similar response rates to therapy with checkpoint inhibitors as other cohorts. J. Clin. Oncol..

[329] Ascierto P.A., McArthur G., Dreno B., Atkinson V., Lizskay V., Di Giacomo A.M., Mandalà M., Demidov L., Stoyakovsky D., Thomas L. (2017). Cobimetinib combined with vemurafenibin davanced BRAF^V600^-mutant melanoma (coBRIM): Updated efficacy results from a randomised, double-blind, phase 3 trial. Lancet Oncol..

[330] Long G.V., Hauschild A., Santinami M., Atkinson V., Mandalà M., Chiarion-Sileni V., Larkin J., Nyakas M., Dutriaux C., Haydon A. (2017). Ajuvant dabrafenib plus trametinib in stage III BRAF-mutated melanoma. N. Engl. J. Med..

[331] Long G.V., Eraglu Z., Infante T., Patel S., Dand A., Johnson D.B., Gonzalez R., Kefford R., Hamid O., Schuchter L. (2017). Long-term outcomes in patients with BRAF V600-mutant metastatic melanoma who received dabrafebib combined with trametinib. J. Clin. Oncol..

[332] Davies M.A., Saiag P., Robert C., Grob J.J., Flaherty K.T., Arance A., Chiarion-Sileni V., Thomas L., Lesimple T., Mortier L. (2017). Dabrafenib plus trametinib in patients with BRAF^V600^-mutant melanoma brain metastases (COMB-MB): A multicenter, multicohort, open-label, phase 2 trial. Lancet Oncol..

[333] Schreuer M., Jansen Y., Pauken S., Chevrolet I., Seremet T., Krurse V., Neyns B. (2017). Combination of Dabrafenib plus Trametinib for BRAF and MEK inhibitor treated patients with advanced BRAF V600-mutant melanoma: An open òlabel, single arm, dual-centre, phase 2 clinical trial. Lancet Oncol..

[334] Kong X., Kuilman T., Shahrabi A., Boshuizen J., Kemper K., Song Y., Niessen H., Rozeman E., Foppen M., Blank C., Peeper D. (2017). Cancer drug addiction is relayed by an ERK2-dependent phenotype switch. Nature.

[335] Hong A., Moriceau G., Sun L., Lomely S., Piva M., Daimoseaux R., Holmen S.L., Sharpless N.E., Hugo W., Lo R.S. (2017). Exploiting drug addiction mechanisms to select against MAPKi-resistant melanoma. Cancer Discov..

[336] Smith M.P., Brunton H., Rowling E., Ferguson J., Arozarena I., Miscolczi Z., Lee J., Girotti M., Marais R., Levesque M. (2016). Inhibiting drivers of non-mutational drug tolerance is a salvage strategy for targeted melanoma therapy. Cancer Cell.

[337] Smith M., Rowling E., Miskolczi Z., Ferguson J., Spoerri L., Haas N., Sloss O., McEntegart S., Arozarena I., von Kriegsheim A. (2017). Targeting endothelin receptor signalling overcomes heterogeneity driven therapy failure. EMBO J..

[338] Esciocak B., McMillan E., Mendiratta S., Kollipara R., Zhang H., Humpries C., Wang C., Rodriguez J.G., Ding M., Zaman A. (2017). Biomarker accessible and chemically addressable mechanistic subtypes of BRAF melanoma. Cancer Discov..

[339] Piskounova E., Agathocleous M., Murphy M.M., Hu Z., Huddlestun S.E., Zhao Z., Leitch A.M., Johnson T.M., DeBerardinis R.J., Morrison S.J. (2015). Oxidative stress inhibits distant metastasis by human melanoma cells. Nature.

